# Multicenter Phase II Study Evaluating Two Cycles of Docetaxel, Cisplatin and Cetuximab as Induction Regimen Prior to Surgery in Chemotherapy-Naive Patients with NSCLC Stage IB-IIIA (INN06-Study)

**DOI:** 10.1371/journal.pone.0125364

**Published:** 2015-05-28

**Authors:** Wolfgang Hilbe, Georg Pall, Florian Kocher, Andreas Pircher, August Zabernigg, Thomas Schmid, Michael Schumacher, Herbert Jamnig, Michael Fiegl, Anne Gächter, Martin Freund, Dorota Kendler, Claudia Manzl, Bettina Zelger, Helmut Popper, Ewald Wöll

**Affiliations:** 1 Medical University Innsbruck, Department of Internal Medicine V (Haematology and Oncology), Innsbruck, Austria; 2 Department of Internal Medicine, County Hospital Kufstein, Kufstein, Austria; 3 Department of Surgery, Medical University Innsbruck, Innsbruck, Austria; 4 Department of Pneumology, General Hospital, Elisabethinen Linz, Linz, Austria; 5 Department of Pneumology, County Hospital Natters, Natters, Austria; 6 Department of Radiology, Medical University Innsbruck, Innsbruck, Austria; 7 Nuclear Medicine, Medical University Innsbruck, Innsbruck, Austria; 8 Department of Pathology, Medical University Innsbruck, Innsbruck, Austria; 9 Department of Pathology, Medical University Graz, Graz, Austria; 10 Department of Internal Medicine, Saint Vincent Hospital Zams, Zams, Austria; 11 Tyrolean Cancer Research Institute, Innsbruck, Austria; University Campus Bio-Medico, ITALY

## Abstract

**Background:**

Different strategies for neoadjuvant chemotherapy in patients with early stage NSCLC have already been evaluated. The aim of this study was to evaluate the tolerability and efficacy of a chemoimmunotherapy when limited to two cycles.

**Methods:**

Between 01/2007 and 03/2010 41 patients with primarily resectable NSCLC stage IB to IIIA were included. Treatment consisted of two cycles cisplatin (40 mg/m^2^ d1+2) and docetaxel (75 mg/m^2^ d1) q3 weeks, accompanied by the administration of cetuximab (400 mg/m^2^ d1, then 250 mg weekly). The primary endpoint was radiological response according to RECIST.

**Results:**

40 patients were evaluable for toxicity, 39 for response. The main grade 3/4 toxicities were: neutropenia 25%, leucopenia 11%, febrile neutropenia 6%, nausea 8% and rash 8%. 20 patients achieved a partial response, 17 a stable disease, 2 were not evaluable. 37 patients (95%) underwent surgery and in three of them a complete pathological response was achieved. At a median follow-up of 44.2 months, 41% of the patients had died, median progression-free survival was 22.5 months.

**Conclusions:**

Two cycles of cisplatin/ docetaxel/ cetuximab showed promising efficacy in the neoadjuvant treatment of early-stage NSCLC and rapid operation was possible in 95% of patients. Toxicities were manageable and as expected.

**Trial Registration:**

EU Clinical Trials Register; Eudract-Nr: 2006-004639-31

## Introduction

Complete resection of non-small cell lung cancer (NSCLC) is the most important curative option available, but can only be offered to a minority of patients. However, even for very early stages of disease, a high percentage of patients will inevitably relapse. The 5-year survival rate for resected pathological stage I-IIA ranges from 73% to 46%, but decreases to 36% in stage IIB, 24% in IIIA and is less than 10% in patients with stage IIIB [1]. These poor long-term results in NSCLC have led to the evaluation of perioperative systemic treatment approaches like induction chemotherapy, chemoradiotherapy and adjuvant chemotherapy.

From a historical perspective one of the most relevant neoadjuvant studies was a phase III trial comparing mitomycin/ ifosfamid/ cisplatin for two cycles and subsequent surgery with immediate surgery. Differences seen in median survival favored the neoadjuvant chemotherapy [2]. Encouraged by these results the lung cancer community initiated a series of randomized trials testing modern antineoplastic agents in this setting [2–7].

The activity and toxicity of cisplatin/ docetaxel was evaluated in a neoadjuvant setting including IIIA (N2) patients [8]. Three cycles were administered and an overall response rate of 66% was achieved, 19% proved to have a complete pathological response and overall survival was 33 months. This very promising data justified further investigation of these neoadjuvant strategies. Since the addition of cetuximab to standard chemotherapy with cisplatin/ vinorelbine significantly improved efficacy in advanced stages [9], it would be reasonable to evaluate a similar chemo-combination with cetuximab in earlier stages.

Consequently, the first aim of the present study was to evaluate whether two cycles of neoadjuvant cisplatin/ docetaxel/ cetuximab are efficacious in NSCLC. The second aim was to define the relevance and feasibility of an intensified staging procedure including computed tomography (CT), 18-fluoro-2-deoxyglucose-positron emission tomography (PET) and cytological or histological evaluation of N2 lymph nodes by mediastinoscopy (MSC) or endobronchial ultrasound-guided transbronchial biopsy (EBUS).

## Patients and Methods

The protocol for this trial and supporting CONSORT checklist are available as supporting information; see S1 TREND Checklist and S1 Protocol.

Between 01/2007 and 03/2010 this multicenter, prospective phase II study was performed in five participating centers in Austria. Patients with anatomically and functionally resectable NSCLC stages IB to IIIA were eligible for inclusion. The protocol, patient information and consent were approved by the local ethics committee of Medical University Innsbruck and by the Austrian regulatory agency (Eudract Nr.:2006-004639-31). Written informed consent was obtained from all patients. NSCLC was confirmed by histologic verification and eligibility and operability were assessed by a multidisciplinary tumor board. TNM staging according to the 6^th^ edition [10] was performed on the basis of CT scans (chest, abdomen and brain), PET-scan, bronchoscopy and MSC (optionally EBUS). Other eligibility criteria included: age between 18–80 years, WHO performance status 0–2, adequate respiratory function (sufficient for necessary surgical treatment), adequate hematological function (Hb >10 g/dl, ANC >2.0 x 10^9^/L, platelets > 100 x 10^9^/L), adequate renal and hepatic functions (total bilirubin ≤ 1.5 x UNL, serum creatinine within normal limits, creatinine clearance >60 ml/min, ASAT and ALAT <2.5 x UNL, alkaline phosphatase <5 x UNL). Main exclusion criteria were: history of prior malignancies, serious concomitant illnesses or medical conditions (e.g. congestive heart failure, angina pectoris, significant neurologic or psychiatric disorders, uncontrolled diabetes mellitus), and prior therapy for NSCLC. Imaging, bronchoscopy, MSC and pulmonary function testing were performed within three weeks and laboratory investigations were done within seven days prior to first neoadjuvant treatment.

Treatment consisted of two cycles of the combination of cisplatin (40 mg/m², day 1 and 2), docetaxel (75 mg/m², day 1) every 3 weeks and cetuximab (400 mg/m², day 1 followed by 250 mg/m² weekly thereafter).

The primary efficacy variable, defined as ORR (overall response rate, complete plus partial response) was determined by the percentage of patients achieving objective responses according to RECIST [11]. All patients starting with at least one cycle of neoadjuvant chemotherapy as induction were considered as evaluable for efficacy and tolerability.

The median of progression free survival (PFS) and overall survival (OS) were estimated using the Kaplan-Meier method. PFS and OS according to the different parameters were compared using a log rank test (univariate analysis). PFS was defined as time from first dose of neoadjuvant treatment to disease progression or death, whichever occurred first. OS was measured from study inclusion until death by any cause. A value of p<0.05 was considered to be statistically significant. P-values were not adjusted for multiple comparisons. All analyses were performed using SPSS software version 21 (Armonk, NY: IBM Corp.).

“Clinical staging” included CT-scans to characterize the T-, N- and M-status. PET-scan, MSC or EBUS were added for the exact definition of the nodal status. At the interdisciplinary board diagnostic results were discussed and the final stage was defined as “comprehensive tumor stage”.

## Results

### Patients´ demographics and baseline characteristics (n = 39)

**Fig 1:**
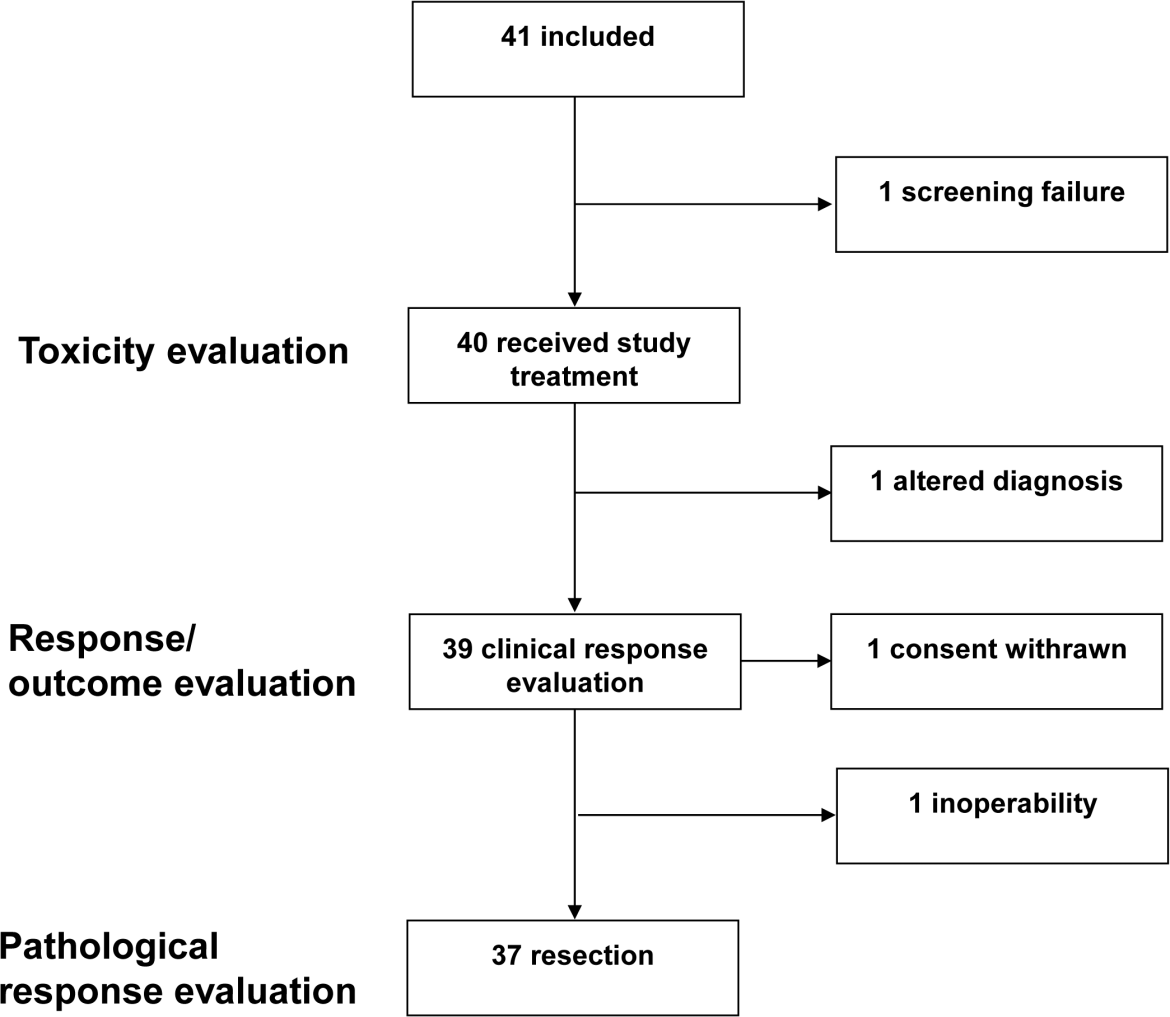
Participant diagram. Paricipant diagram describing patients analyzed in the INN-06 study.

Overall, 41 patients were included into the study. However, one patient did not fit the inclusion criteria (elevated liver parameters) and was excluded. Thus, 40 patients were evaluable for toxicity and 39 patients were evaluable for efficacy because one patient had to be excluded due to revision of diagnosis with definitive classification on the surgical specimen as a melanoma **(Fig 1)**.

Detailed characteristics of patients included in efficacy evaluation are presented in **Table 1.** Interestingly, the additional use of MSC, EBUS and PET-scans led to a stage shift in 12 cases (31%): Eight patients (21%) shifted to a lower and four patients (10%) to a higher stage.

**Table 1:** Baseline demographics and clinical characteristics (n = 39).

	n	%
**Mean age (range), years**	57.5 (34–78) years	
**Gender**		
*Male*	24	61
*Female*	15	39
**WHO**		
*0*	29	74
*1*	10	26
**Histologic subtypes**		
*Adenocarcinoma*	25	64
*Squamous-cell carcinoma*	12	31
*Large cell carcinoma*	1	3
*Adenosquamous carcinoma*	1	3
**Clinical Stage**		
*IB*	5	13
*IIA*	2	5
*IIB*	10	26
*IIIA*	20	51
*IIIB*	2	5
Comprehensive Stage*		
*IB*	3	8
*IIA*	2	5
*IIB*	16	41
*IIIA*	18	46
*IIIB*	-	-

“Clinical staging” included CT only.

*“Comprehensive staging” included CT, PET, MSC or EBUS.

### Toxicities of neoadjuvant therapy (n = 40)

40 patients were evaluable for toxicities, including the one patient with the revised diagnosis of melanoma in the definitive surgical specimen. Adverse Events (AE) are detailed in **Table 2**. Altogether 336 AEs were documented. The majority of AE were mild, with grade I and grade II in 180 (53%) and 114 (34%) of events. 31 (9%) and 10 (3%) events were grade III or grade IV respectively. Within the 78 applied chemo-immunotherapy cycles, a mean of 4.3 AEs per treatment cycle was reported (mean AE per cycle applied: grade I: 2.3; grade II: 1.5; grade III: 0.4; grad IV 0.1). A single patient developed a grade IV-hypersensitivity reaction (anaphylactic shock) immediately after the first application of cetuximab and was therefore excluded from further neoadjuvant treatment. Infectious complications were mainly mild, one patient experienced MRSA sepsis and esophagitis grade III. Alongside, two events with urinary tract infection (grade I: n = 1; grade II: n = 1) and four events with infection of the respiratory tract (grade I: n = 3; grade II: n = 1) were reported.

**Table 2:** Adverse Events (n = 40).

	Grade I-II		Grade III		Grade IV	
	n	%	n	%	n	%
**Hematolgic Toxicities**		** **	** **	** **	** **	
*Anemia*	8	20	1	3	-	-
*Leukopenia*	8	20	3	8	1	3
*Neutropenia*	5	13	6	15	4	10
*Febrile neutropenia*	-		1	3	1	3
*Thrombopenia*	1	3	-	-	-	-
**Non-Hematologic Toxicities**						
*Allergic reactions*	3	8	-	-	1	3
*Exanthema/Rash*	32	80	3	8	-	-
*Nausea/Emesis*	26	75	3	8	-	-
*Diarrhea*	14	35	1	3	-	-
*Obstipation*	10	25	-	-	-	-
*Mucositis/Stomatitis*	22	55	-	-	-	-
*Polyneuropathy*	6	15	-	-	-	-
*Depression/Sleeping disorders*	14	35	-	-	-	-
*Fatigue*	18	45	1	3	-	-
*Appetite loss*	13	33	-	-	-	-
*Urinary Tract infection*	2	5	-	-	-	-
*Paresis recurrent laryngeal nerve*	-	-	1	3	-	-
*Esophagitis*	-	-	1	3	-	-
*Sepsis*	-	-	1	3	-	-
*Acetabulum fracture*	-	-	1	3	-	-
*Dyspnea*	3	8	1	3	-	-
*Laboratory disturbancies*	18	45	3	8	3	8
*Arthralgia*	2	5	1	3	-	-
*Chest pain*	3	8	1	3	-	-
*Reflux*	5	13	1	3	-	-
*Alopecia*	9	23	1	3	-	-
*Respiratory infection*	4	10	-	-	-	-
*Other toxicities*	68	-	-	-	-	-

### Response to neoadjuvant treatment (n = 39)

39 patients were evaluable in regard to clinical response. According to RECIST 20 patients (51%) revealed a partial response (PR), 17 patients (44%) were classified as stable disease (SD) and no patient experienced disease progression (PD). Two patients (5%) were not evaluable due to premature withdrawal of consent and the patient with anaphylactic shock following cetuximab infusion due to immediate surgical treatment thereafter. Of the 17 patients with SD all showed some tumor regression, eight (47%) among those even found a 20–30% regression according to RECIST. In 2012, Pirker et al. published a immunohistochemistry score for EGFR expression that proved to be predictive in NSCLC patients receiving cetuximab [12]. Utilizing this score in 22 evaluable patients, we compared the EGFR expression (low vs. high) to the respective response according to RECIST. No significant correlation between response and EGFR expression was observed (p = 0.746).

The pathological staging following surgery (ypTNM) was then compared with the original comprehensive staging, which included histological or cytological proven nodal evaluation, and was defined as “pathological response”. Of 37 evaluable patients, 29 (78%) showed a stage-shift between initial evaluation and pathological staging and eight patients remained stable (22%). In 22/37 patients (59%) a pathological down-staging was achieved, in seven patients (19%) the pathological stage was higher as originally defined by comprehensive staging. Differences of the latter group were based on following changes: T2 or T1 were then classified as T4 (n = 3); one case proved to have pulmonary metastases (M1) and in three cases the nodal status had increased (N0 to N1 n = 1; N1 to N2 n = 2).

### Surgical Treatment and adjuvant therapy (n = 37)

Of 39 NSCLC patients who underwent neoadjuvant treatment, surgical resection was performed in 37 patients (95%). The patient with severe hypersensitivity reaction during cycle one was operated immediately thereafter and is included in further analysis. In one patient the thoracic surgeons diagnosed inoperability during surgical intervention and one patient withdrew informed consent. Median time between first day of neoadjuvant therapy and surgery was 2.2 months (range, 0.9–3.6 months). Most importantly, in three patients pathological CR was observed (3/37; 8%). Pathological stages were classified as stage I, II, IIIA, IIIB and IV in 12 (32%), 13 (35%), 5 (14%), 3 (8%) and 1 (3%) patients, respectively. Major perioperative complications occurred in two patients (5%). One patient had a protracted leakage of bronchogenic anastomosis, the other patient suffered from recurrent laryngeal nerve paralysis due to surgery.

Two cycles of adjuvant treatment were applied in 26 cases (70% of surgically treated patients) according to the physicians´ discretion: cisplatin/ docetaxel (n = 19), platin/ etoposide (n = 6), carboplatin/ gemcitabine (n = 1).

### Survival

At time of final analysis (12/2013) 16 of 39 evaluable patients (41%) had deceased. Median time of follow-up in the cohort is 44.2 months. Median PFS was 22.5 months (95% CI 10.3–34.7) months. Noteworthy, in 17 patients (44%) no tumor progression has been detected until last follow-up. Median OS has not been reached and 5-year OS was 58%.

**Fig 2:**
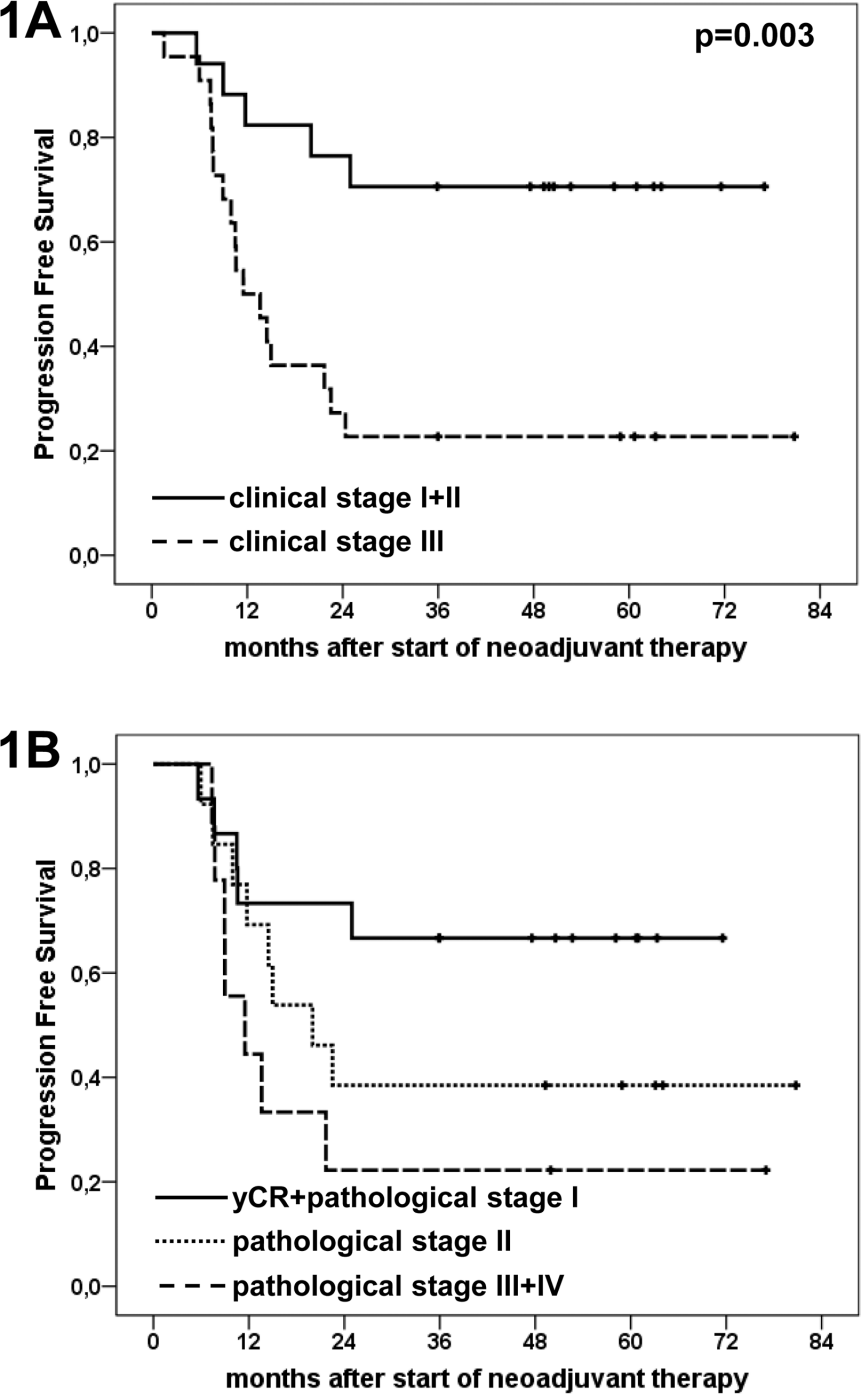
Kaplan-Meier plots illustrating progression free survival according to clinical and pathological stages. **Fig 2A**: median PFS according to clinical stages (n = 39): stages I+II: not reached, stage III 11.5 months; p = 0.003. **Fig 2B**: median PFS according to pathological stages (n = 37): yCR [complete pathological response] + stage I not reached, stage II 20.0 months, stage III+IV 11.5 months. In pairwise comparison of the respective groups a significant difference was found between yCR+stage I and stage III+IV with a p-value of 0.037.

Univariate analysis was performed in order to delineate factors with prognostic impact. The following baseline (pre-therapy) parameters were subjected to analysis: gender, WHO- score clinical and comprehensive stage. Only clinical stage was significantly prognostic in regard to PFS (for stages I/II, median PFS not reached; stage III, 11.5 months; p = 0.003) **(Fig 2A)**. In regard to OS, clinical and comprehensive stages at initial diagnosis were significantly prognostic (I/II vs. III). In detail, median OS in clinical stages I/II was not reached, whereas in clinical stage III, median OS was 29.9 months (p = 0.05). Likewise, median OS in comprehensive stages I/II was not reached and in stage III it was 29.9 months (p = 0.037).

For the next step, therapy associated parameters were evaluated (RECIST, ypTNM stage, pathological downstaging). Interestingly, only pathological stage yCR+stage I vs. stage III/IV, as treatment related parameter was significantly associated with OS and PFS **(Fig 2B)** (median OS, yCR/stage I: n.r., stage II: n.r., stage III/IV: 28.9 months, p = 0.028; median PFS, yCR/stage I: n.r., stage II: 20.0 months, stage III/IV: 11.5 months,p = 0.037).

## Discussion

This study clearly demonstrates substantial activity of two cycles of cisplatin/ docetaxel combined with cetuximab in the neoadjuvant treatment of patients with primary resectable NSCLC. Based on RECIST 20 of 39 patients (51%) revealed a partial response and 44% were classified as stable disease. None of the patients showed tumor progression at radiographical examinations prior surgical treatment. Thus, no patient missed his/her therapeutic window of surgical intervention. Only in one patient, who initially was regarded as a borderline stage IIIA, inoperability had to be affirmed during thoracotomy. Other neoadjuvant trials had administered three cycles of chemotherapy and achieved a PR rate between 35% and 66%, respectively [5–8]. At a median follow-up of 44.2 months, the present cohort proved a median PFS of 22.5 months. Other neoadjuvant trials reported a PFS of 26 [7], 33 [6] and 40 months [5]. The excellent PFS data of the latter study might have been achieved, as the majority of patients were classified as stage I and II and only 5% were stage III patients. The MRC trial included 8% and the present trial classified 46% of the patients to be in stage III [7]. Therefore, concerning efficacy, the two cycle combination compares well with previous results seen after the administration of three neoadjuvant cycles.

In the palliative setting the addition of cetuximab resulted in an improved survival when added to chemotherapy with cisplatin/ vinorelbin [9]. However, based on the single arm phase 2 concept of this trial, the suggested additive effect of cetuximab to the cisplatin/ docetaxel chemotherapy cannot be answered. The high rate of responses seen without any refractory cases at least supports our assumption.

For the present trial a comprehensive staging was mandatory. Therefore CT was combined with FDG-PET analysis (PET-CT optional) and every patient had to be staged by MSC or EBUS for optimal N2/3 lymph node evaluation. Standard staging with CT alone was then compared with “comprehensive staging”, which included PET and MSC/EBUS and found that eight patients (21%) shifted to a lower and four patients (10%) to a higher stage. Hence, our trial clearly showed that intensified staging procedures might change the clinical stage in about one third of the cases. In other trials pretreatment evaluation did not routinely include PET [6,7,13]. In our opinion standard staging with CT was clearly limited in defining the correct nodal involvement and frequently led to an over-interpretation with false-positive N2 nodes. In our trial, for example CT staging classified 22 cases as having a stage III, including all other diagnostic parameters only 18 cases remained. The importance of accurate nodal staging was also pointed out at ASCO 2013 [14] and the authors indicated, that “the risk of clinical over-staging cannot be exaggerated as false positive rates of nearly 50% have been seen with CT alone compared to 16% with CT-PET evaluation”. They stated that every patient with N2 involvement, who would otherwise be a candidate for surgery, should undergo nodal biopsies.

Designing the study, we hypothesized that two cycles of chemotherapy would prove acceptable tolerability. Comparing two cycles of cisplatin/ docetaxel/ cetuximab in the present trial with a three cycle neoadjuvant regimen containing cisplatin/ docetaxel as reported by Betticher et al. [8] and six cycles treatment with cisplatin/ docetaxel as administered in the palliative approval trial [13], a lower rate of nausea (grade 3: 8%, 12%, 18%), fatigue (grade 3: 3%, n.r., 12%) and leucopenia (grade 3–4: 25%, 33%, 43%) was observed. The carboplatin/ paclitaxel neoadjuvant SWOG combination resulted in a grade 3/4 neutropenia of 48% [6] and the cisplatin/ gemcitabine combination in a grade 3/4 neutropenia of 26% and thrombocytopenia of 11% [5]. However, the addition of cetuximab induced other toxicities like allergic reactions, exanthema/rash and diarrhea. In one patient even a severe allergic reaction occurred. Nevertheless, the cisplatin/ docetaxel/ cetuximab combination was feasible with an acceptable toxicity profile, if administered for two cycles.

Regarding the fact that two large prospective trials failed to complete their recruitment, it might be speculated that most patients prefer tumor resection as soon as possible [5,6]. The authors of these trials argued that the upcoming results of the adjuvant trials had changed treatment preferences. The presented two-cycle treatment strategy met both, immediate start of systemic anticancer treatment and no substantial delay of surgery. At the best the minimal interval between start of chemotherapy and surgery was as short as six weeks (median 2.2 months). Taking into account that surgical interventions are not immediately available, a “preoperative” delay of two months with at least disease stabilization seems to be justifiable. Moreover, the low toxicity experienced in our trial enabled subsequent surgery in 95% of the cases. Nevertheless, in singular cases complications like infection, pulmonary embolism or hip fracture led to a delay of the planned surgical intervention.

The optimal number of chemotherapeutic cycles however is still discussed controversially. For the adjuvant setting four cycles are considered to be the standard approach [15]. Concerning neoadjuvant chemotherapy, most trials administered three cycles [5–8]. Recently, Steger et al. [16] compared two different neoadjuvant protocols in a single center retrospective study and concluded that the more aggressive protocol showed a better downstaging effect but survival difference did not reach significance. A two cycle strategy has been evaluated by a French group comparing mitomycin/ ifosfamid/ cisplatin with surgery alone [2]. This trial was recently updated proving an 8% survival benefit with a 10- year survival rate of 2% vs. 21% [17].

Whether additional cycles in the adjuvant setting after two cycles of neoadjuvant therapy are beneficial, remains unclear. Based on this ambiguity the treating physicians of our group frequently decided to administer two cycles of adjuvant treatment (n = 26; 70%) within the follow-up period. In this context results of PFS and OS might have been influenced by the high rate of additional adjuvant treatment.

In conclusion, this multicenter phase 2 trial shows that two cycles of neoadjuvant cisplatin/ docetaxel/ cetuximab are effective and well tolerated in stage IB-IIIA patients. This short term treatment fits perfectly into the multimodal therapy concept which aims to optimize long term results for the patients after complete resection of NSCLC. In our opinion neoadjuvant strategies should further be developed and must include the most effective drugs available.

## Supporting Information

S1 TREND Checklist TREND Checklist. (PDF)

S1 Protocol Trial Protocol. (RTF)
