# Supplementary material for: Multicenter Phase II Study Evaluating Two Cycles of Docetaxel, Cisplatin and Cetuximab as Induction Regimen Prior to Surgery in Chemotherapy-Naive Patients with NSCLC Stage IB-IIIA (INN06-Study)
Source: PLoS One. 2015 May 28;10(5):e0125364. doi: 10.1371/journal.pone.0125364 (PMC4447267; doi:10.1371/journal.pone.0125364)
Supplement: S1 Protocol — (RTF) [file pone.0125364.s002.rtf]

CLINICAL STUDY PROTOCOL

MULTICENTER PHASE II STUDY EVALUATING DOCETAXEl, CDDP, AND CETUXIMAB AS INDUCTION REGIMEN PRIOR TO SURGERY IN CHEMONAIVE PATIENTS WITH NSCLC STAGE IB, II, AND IIIA   

Running Title: “INN 06 – Study (Immune Response on Neoadjuvant Therapy in NSCLC)”


SPONSOR: 	TAKO (Tiroler Arbeitskreis Onkologie), 
Chairman: Univ. Prof. Dr. Peter Lukas
www.tako.or.at

STUDY CENTERS:	Medical University Innsbruck
			LKH Natters
			BKH Zams
			BKH Kufstein
			KH der Elisabethinen Linz
			AKH Linz
			Klinikum Wels-Grieskirchen

Supported by:	Fa. SANOFI-AVENTIS
                         	Fa. MERCK

FINAL VERSION: 	21.08.06
AMENDMENT 1	02.07.07
AMENDMENT 2	30.04.08
AMENDMENT 3	10.10.08


EudraCT Number:	2006-004639-31


PRINCIPAL INVESTIGATOR		HILBE, Univ.-Prof. Dr. W.
INNSBRUCK				Medical University Innsbruck
Coordinator:			Dept. Haematology and Oncology
Anichstr. 35, A-6020 Innsbruck
Tel.: 0512-504-81151
Fax: 0512-504-23431
Wolfgang.Hilbe@i-med.ac.at

PRINCIPAL INVESTIGATOR		Jamnig, Prim. Dr. H.
LKH NATTERS:			

PRINCIPAL INVESTIGATOR		Zabernigg, OA Dr. A.
BKH Kufstein			

PRINCIPAL INVESTIGATOR		WÖLL, Prim. Univ.-Doz. Dr. E.
BKH Zams:				

PRINCIPAL INVESTIGATOR		AIGNER, Prim. MR Dr. K.
KH der Elisabethinen, Linz:	
			
PRINCIPAL INVESTIGATOR		SCHINKO, Prim. Dr. H.
LKH Linz:
				
PRINCIPAL INVESTIGATOR		KOLB, OA Dr. R.
Klinikum Wels-grieskirchen:				

CO-INVESTIGATORS:		

Medical University Innsbruck		Medical University Innsbruck
Dept. of General Internal Medicine  	Dept. Hematology and Oncology
BECHTER, Univ.-Doz. Dr. O.		GASTL, Univ.-Prof. Dr. G.
EISTERER, Univ.-Prof. Dr. W.		WOLF, Dr. D.
PALL, OA Dr. G.			
ZWIERZINA, Univ.-Prof. Dr. H.

Medical University Innsbruck  		Medical University Innsbruck   	
Dept. of Surgery			Dept. Nuclear Medicine
LUCCIARINI, OA Dr. P.			VIRGOLINI, Univ.-Prof. Dr. I.
SCHMID, Univ.-Prof. Dr. T. 		KENDLER, OA Dr. D.
					
Medical University Innsbruck		LKH Natters    
Dept. Pathology   			Dept. Internal Medicine				
STERLACCI, Dr. W.	                      	DENZ , Prim. Univ.-Doz. Dr. H.
ZELGER, Univ. Prof. Dr. B.		FIEGL, Univ.-Doz. Dr. M.

LKH Natters				BKH Zams				  
Dept. Pulmonology			Dept. Internal Medicine			
HACKL, OA Dr. M.			SCHEIBER, Dr. R.
					SCHOENHERR, OA Dr. H-R
		
BKH Kufstein				Medical University Innsbruck
Dept. Internal Medicine			Dept. of Radiology	 
SCHRANZHOFER, Dr. R.		FREUND, OA Dr. M.
GATTRINGER,  Prim.Univ.-Prof.Dr.K	FRANK, OA Dr. R.

KH d. Elisabethinen Linz		LKH Linz
Dept. Pulmonology			Dept. Pulmonology	 
FORSTNER, OA Dr. B.			KROPFMÜLLER, OA, Dr. R.
DULLER, OA Dr. W.

Klinikum Wels-Grieskirchen
Dept. Pulmonoloy
Eckmayr, Prim. Dr. J.
KOLB, OA Dr. 
		
LABORATORY
COLLABORATORS:			WOLF, Dr. A.-M.
					RUMPOLD, Dr. H.
					Medical University Innsbruck
					Dept. Hematology and Oncology

					DLASKA, Dr. M.
					Pircher, Dr. A.
					LÖFFLER, Dr. J.
					Medical University Innsbruck
					Dept. Internal Medicine

Contact: “Labor für molekulare Zellbiologie”, Dr. Pircher, Dr. Dlaska
Phone: 0512-504-25611
Fax.: 0512-504-25612

MONITORING:				HOSCHEK, Dr. S.
					KH Hochzirl

					FRAIDL, Mag. G.
					GAECHTER, A.
					Medical University Innsbruck
					Dept. Internal Medicine

STATISTICS:				HILBE, Univ.-Prof. Dr. W.
					ULMER, Univ.-Prof. Mag. Dr. H.

STUDY- COORDINATION:		FRAIDL, Mag. G.
					GAECHTER, A.
					Medical University Innsbruck
					Dept. Internal Medicine
Phone: 0512-504-23333
Fax.: 0512-504-23431

Assessment	Baseline
Prior to Inclusion	During  Induction Therapy	End of Induction Therapy	Surgery  	End of Treatment
>30  <45 days
Post Surgery	Follow-up (6)	

History / Physical Examination/Vital Signs (weight, blood pressure, heart rate, body temperature) 	

within 7 days 	

every cycle	

X	

X	

X	
	
Hematology
WBC, neutrophils, hemoglobin, platelets	
within 7 days 	
weekly	
X	
end of surgery	
X	
(2)	
Immunology (3)	S, H (40 mL)	S weekly
H weekl (5 mL)
per cycle 20 mL	S, H (20 mL)		S, H (20 mL)		
Proteomics (4)	S	S per cycle (7)	S	(8)	S		
Skin Biopsy (optional)		(7)	(7)	(7)			
Immunohistochemistry (5)				Tissue Probe			
Biochemistry
protein, S-creatinine, crea. cl. (if indicated), S-electrolytes (K, Na, Cl, Ca, Mg), alk. phos, ASAT, ALAT, total bili., GT, LDH, CRP, and  tumor marker (optional)	
within 7 days 	
every cycle	
X	
end of surgery 	X 		
Radiology (1):

Chest-CT
Abdominal CT-scan
PET-scan 

Brain CT-scan (optional)	
within 21 days
X
X
X

if indicated	


	

X
X
X

if indicated	
} if indicated	
 } if indicated		
Bronchoscopy	within 21 days	 	optional		 		
Mediastinoscopy/selective puncture of mediastinal lymphnodes	within 21 days  			 			
Lung-function-tests	within 21 days 	 	X	if indicated 	if indicated		
ECG	within 7 days 	if  indicated	X	if indicated 	if indicated		
Neurological Examination
(may be done by internist)	within 7 days 	every cycle	X	end of surgery  	X		
Symptoms / Adverse Events	within 7 days 	every cycle	X	end of surgery 	X		
Concomitant Therapy	within 7 days 	every cycle	X	end of surgery 	X		
Informed Consent	x						
Pregnancy Test	x	every month	every month	every month	X	(2)	
(1)	To ensure comparability for assessment of response, the baseline and subsequent radiology must be performed using identical techniques and methodology
(2)	According to local standards. Last follow up and death will be documented. 
(3)	S (Serum, 5 mL), H (heparinized blood, 40 mL baselline, otherwise 20 mL), Cx = Chemotherapy
(4)	S (Serum, 5 mL) 
(5)	At least 0.3 x 0.3 x 0.3 cm, in PBS or NaCl , no formalin  send to “Labor für molekulare Zellbiologie”.
(6)	Follow-up will be perfomed acording to local recommendations (out of study)
(7)	In  ca	se of skin toxicities NCI CTC ≥2  biopsies of EGFR mediated skin toxicity and control recommended
(8)	During surgery normal skin biopsy recommended	
Abbreviations and definitions
AE 	Adverse Event	
ALAT	Alanine aminotransferase (also referred to as SGPT)	
AMG	Arzneimittelgesetz (German Medicines Act)	
ASAT	Aspartate aminotransferase (also referred to as SGOT)	
AUC	Area under the curve	
Cmax	Maximum concentration	
CT	Computed Tomography	
DNA	Deoxyribonucleic acid	
ECG	Electrocardiogram	
EDTA	Ethylene-diaminetetraacetic acid	
EOS	End of study	
EOT	End of therapy	
ESR	Erythrocyte Sedimentation Rate (after 1 hour)	
FDA	Food and Drug Administration (USA)	
FEV1	Fractional expiratory volume in the first second	
GCP	Good Clinical Practice	
GGT	Gamma-glutamyl-transferase	
ICH	International Conference on Harmonization of technical requirements for registration of pharmaceuticals for human use	
ICU	Intensive Care Unit	
IEC	Independent Ethics Committee	
INR	International Normalised Ratio of Prothrombin Time/Control Prothrombin Time	
ITT	Intent-to-Treat (analysis)	
IV	Intravenous	
lat	Lateral (chest X-ray view)	
LDH	Lactate dehydrogenase 	
LPCA	Last predefined change abnormal 	
LRT	Lower Respiratory Tract 	
NCCLS	National Committee for Clinical Laboratory Standards of the US 	
pa	Posterior-anterior (Xray view)	
PC	Predefined change	
PCA	Predefined change abnormal 	
PMN	Polymorphonuclear cell 	
PTT 	Partial thromboplastin time	
RBC
RR	Red blood cell (count) Erythrocytes
Response Rate	
SAE	Serious adverse event	
SI	Standard international (units)	
SAPS	Simplified acute physiological score	
 	 	
t½	Elimination half-life	
ULN	upper limit of normal	
WBC	White blood cell (count)	
 	 	
		
		
		
		
		
 	 	
		
		
		
		
 	 	


Index

1.	Introduction and study rationale	11
1.1.	INTRODUCTION	11
1.2.	INDUCTION / NEOADJUVANT CHEMOTHERAPY IN NSCLC	11
1.3.	SEQUENTIAL CHEMOTHERAPY AND RADIOTHERAPY	12
1.4.	Concurrent chemoradiotherapy	13
1.5.	Conclusion	13
1.6.	Docetaxel	14
1.7.	CISPLATIN / CDDP	19
1.8.	Cetuximab	20
1.9.	RATIONALE OF THE STUDY	23
2.	Study objectives	25
2.1.	Primary objective	25
2.2.	Secondary objectives	25
3.	Study design	26
4.	Selection of patients	27
4.1.	Number of patients	27
4.2.	Inclusion criteria	27
4.3.	Exclusion criteria	27
5.	Study treatments	29
5.1.	Details of study treatments	29
5.2.	Patients registration	30
5.3.	Blinding	30
5.4.	PREMEDICATION  / PROPHYLACTIC MEDICATION	31
5.5.	CONCOMITANT TREATMENTS	31
5.6.	DOSE MODIFICATIONS AND ACTIONS FOLLOWING TO TOXICITIES	31
6.	Prior and concomitant illnesses and treatments	36
6.1.	Prior and concomitant illnesses	36
6.2.	Prior and concomitant treatments	36
7.	Study procedures and schedule	37
7.1.	Overview of data collection	37
7.2.	Description of study visits	38
8.	Adverse events	41
8.1.	DEFINITIONS	41
8.2.	RECORDING OF ADVERSE EVENTS	41
8.3.	Reporting of serious adverse events	42
8.4.	ANNUAL SAFETY REPORTS	42
8.5.	Subject removal from study therapy due to adverse events	43
9.	Withdrawals	44
9.1.	Withdrawal of patients	44
10.	Statistical procedures	45
10.1.	POPULATION FOR ANALYSIS	45
10.2.	Interim analysis	47
10.3.	Sample size justification	47
11.	Ethical and legal aspects	48
11. 1.   	Good clinical practice	48
11.2.	Delegation of investigator responsibilities	48
11.3.	Patient information and informed consent	48
11.4.	Confidentiality	48
11.5.	Protocol amendments	49
11.6.	Approval of the study protocol and amendments	49
11.7.	Ongoing information for independent ethics committee/ institutional review board	49
11.8.	Premature closure of the study	49
11.9.	Record retention	50
11.10.	Liability and insurance	50
12.	Study monitoring and auditing	51
12.1.   	Study monitoring	51
12.2.	Source data verification and on-site audits	51
13.	Documentation and use of study findings	52
14.	Study duration and dates	52
15.	Authorship	52
16.	Declaration OF investigator	53
	Investigator	53
17.	Appendices	54
17.1.	APPENDIX I: RECIST GUIDELINES FOR EVALUATION OF RESPONSE	54
17.1.4. 	Baseline Documentation of “Target” and “Non-Target” Lesions	55
17.1.5.	Response Criteria	55
17.2.	APPENDIX II: GUIDELINES FOR EVALUATION OF RESECTABILITY ACCORDING TO “TAKO RECOMMENDATONS 2006” (homepage www.tako.or.at)	58
17.3.	APPENDIX III:  NCI CTC Toxicity scale Version 2.0	59
17.4.	APPENDIX VI: STUDY   INCLUSION FORM	77
17.5.	APPENDIX VII: SERIOUS ADVERSE EVENT REPORT FORM	78
17.6.	APPENDIX VIII: WORLD MEDICAL ASSOCIATION DECLARATION OF HELSINKI	79
17.7.	APPENDIX IX: PATIENT INFORMATION AND INFORMED CONSENT	83
17.8.	APPENDIX X: ECOG PERFORMANCE STATUS	88
17.9.	APPENDIX XI: Scientific Project: Definition of chemoresistance by pharmacogenomic testing	89
17.10.	APPENDIX XII: Project Proposal: Immune activation after application of Erbitux in combination with chemotherapy in NSCLC patients – correlation with CD4+CD25+ regulatory T-cells number  and functional activity	94


1.	Introduction and study rationale
1.1.	INTRODUCTION
Lung cancer is the most lethal cancer in both men and women in developed countries  [1]. Approximately 80 % of primary lung cancers are of the non-small variety, which includes squamous cell and undifferentiated large cell carcinomas as well as adenocarcinomas. Only 20% of these cases are suitable for potentially curative resection [2]. The 5-year survival rate for resected stage I-IIa disease ranges from 75% to 60%, but decreases to 59% to 33% in resected stage IIb (T3N0) disease and is less than 10% in patients with stage IIIa N2 disease who undergo resection [3].  In all tumor stages resection of the tumor should be aimed as therapy of choice.  
1.2.	INDUCTION / NEOADJUVANT CHEMOTHERAPY IN NSCLC
The relatively poor long-term results in patients with locally advanced (stage III) NSCLC have led to the evaluation of multimodality treatment approaches (eg., induction chemotherapy, sequential chemoradiotherapy, concurrent chemoradiotherapy). 

The term induction therapy or “neoadjuvant therapy” is defined as a cytoreductive therapy administered before a definitive locoregional treatment (summarized in: Johnson D.H., Turrisi A., Pass H.I., in Lung Cancer: Principles and Practice 1996, Chapter 59, p 863). The concept of induction therapy in locally advanced NSCLC is not new, having been tested in the late 1960s and early 1970s (LC No 14/56). Chemotherapy before surgery has several possible advantages, including: (1) an in vivo assessment of tumour responsiveness (which may have implications for the use of postoperative chemotherapy), (2) potential eradication of clinically occult extrathoracic micrometastases; (3) possible decrease in the incidence of hematogenous spread or local “seeding” of tumor cells caused by surgical manipulation of the primary lesion; (4) the possibility that technically unresectable lesions may become respectable; and (5) possible conservation of normal lung tissue , owing to the surgeon's ability to perform a less extensive resection. [4-6]. Disadvantages of induction chemotherapy include: 1) the morbidity and potential mortality related to the side effects of myelosuppression therapy; 2) increased surgical morbidity or mortality due to chemotherapy-induced or radiotherapy- induced anatomic effects and, perhaps most important, 3) loss of the surgical option if the tumor progresses during induction chemotherapy (Text aus “Lung Cancer, Principles and Practice”, Pass, 1996, pp 867 ff).
Although most trials have been limited to patients with stage III disease, some have included stage IB and II patients. The role of these approaches in early stage disease remains uncertain. A modest survival benefit has been reported in randomized trials when induction (neoadjuvant) chemotherapy is given prior to surgery compared to surgery alone [7-9]. Two randomized clinical trials, each including 60 patients, found a significant survival advantage associated with the use of induction chemotherapy prior to surgery, compared to surgery alone [7,8]. Postoperative RT to all patients in one of the trials [7], and approximately one-half in the other [8]. In both of these studies, the magnitude of the benefit diminished with longer follow-up. The role of induction chemotherapy prior to surgery without postoperative RT was evaluated in a larger French trial, in which 355 patients (188 with stage IB or II, 167 with stage IIIA NSCLC) were randomly assigned to surgery with or without two cycles of preoperative cisplatin-based combination chemotherapy [9]. Induction chemotherapy was associated with a trend toward a longer median disease-free survival (27 versus 13 months, p = 0.15). Survival rates one, two, three, and four years after treatment favoured the induction chemotherapy arm (77 versus 73, 59 versus 52, 52 versus 41, and 44 versus 35 percent, respectively). In subgroup analysis, the survival benefit appeared to be restricted to patients with stage IB or II disease.
Although concurrent chemoradiotherapy has largely replaced induction chemotherapy and surgery in patients with pathologically documented stage III NSCLC, induction chemotherapy may retain a role in selected patients with relatively low-volume mediastinal disease. Recently, preliminary results of two large randomized studies were reported (#7012, Pisters, ASCO 2005; Scagliotti, #7023, ASCO 2005). Both studies included early stages (T2N0 – T3N1) and in both studies 3 cycles of platin combination were applied before surgery. The SWOG trial (#7012) included 354 patients and treatment consisted of Paclitaxel 225 mg/m2 + Carboplatin AUC6 for three cycles. Neoadjuvant treatment resulted in an improvement of median survival (42 vs. 37 months) with a slightly increased 1 year survival probability (82% vs. 79%). The Italian trial (#7023) included 267 patients and therapy was cisplatin 75 mg/m2 + Gemcitabine 1250 mg d1+8 for three cycles. Again an increase of the progression free survival was noted (89 vs. 80 months). Both studies were closed before they reached the planned number of patients due to slow recruitment and therefore statistical analysis failed to prove statistical significant differences. A planned meta-analysis should help to find a clear answer to define the role of neoadjuvant therapy. Facing the actual data of adjuvant therapy [10,11] (#7019, CALGB, Strauss et al., ASCO 2004; #7013, ANITA Trial, ASCO 2005), which proved significant survival benefit, most centers and patients preferred to follow the actual recommendations.

Summing up, there is still a lack of evidence about the clinical impact and treatment modality (chemotherapy or radio-chemotherapy) of the induction therapy.
 
1.3.	SEQUENTIAL CHEMOTHERAPY AND RADIOTHERAPY
Two cooperative group trials and two meta-analyses established that sequential chemotherapy followed by RT improved survival compared with definitive RT alone [12-16]:
·	In Cancer and Leukemia Group B (CALGB) trial 8433, 155 patients with stage III NSCLC were randomly assigned to RT alone (60 Gy over six weeks), or the same RT preceded by two cycles of cisplatin-based chemotherapy [12]. With seven-year follow-up, the addition of chemotherapy was associated with significantly better median and five-year survival (14 versus 10 months and 17 versus 6 percent, respectively). 
·	These results were confirmed in a subsequent Intergroup trial, which randomly assigned 452 patients with stage III disease to conventional RT (60 Gy in 30 daily fractions), hyperfractionated RT (69.6 Gy in 58 fractions of 1.2 Gy each, given twice daily), or chemotherapy (vinblastine plus cisplatin) followed by conventional RT [13,14]. The median survival with sequential chemotherapy and RT was significantly better than with either conventional or hyperfractionated RT alone (13.8 versus 11.4 and 12.3 months, respectively). Although two-year survival rates were significantly higher with sequential treatment (32 versus 19 and 24 percent, respectively), it disappeared in later years. Five-year survival rates were 8, 5, and 6 percent, respectively with sequential chemoradiotherapy, conventional, and hyperfractionated RT, respectively. 
·	Two meta-analyses of trials comparing sequential chemoradiotherapy versus RT or chemotherapy alone also concluded that survival was better with the combined approach at one and two years, but that this did not translate into better long-term outcomes [15,16].

1.4.	Concurrent chemoradiotherapy 
Concurrent administration of chemotherapy and radiotherapy provides early treatment of micrometastatic disease, and exploits the synergistic effect of chemotherapy and RT to enhance local tumor eradication. Randomized trials have established the superiority of this approach compared with sequential chemotherapy and RT, and concurrent chemoradiotherapy has become the preferred approach for most unresected patients with pathologic stage III disease.
Initial concerns about the ability to give full doses of chemotherapy during RT led to the use of reduced doses (see "Low-dose chemotherapy" below). However, subsequent studies showed that full-dose chemotherapy could be given concurrently with manageable toxicity, and this has become the standard approach to concurrent chemoradiotherapy.
Standard-dose chemotherapy — The superiority of concurrent chemoradiotherapy compared with sequential chemotherapy and RT is illustrated by the results of two large multicenter trials [17-19]:
·	In a Japanese study, 320 patients with unresectable stage III NSCLC were randomly assigned to either concurrent chemotherapy (cisplatin, mitomycin, and vindesine) plus thoracic split-course RT (two courses of 28 Gy in 2 Gy daily fractions, separated by ten days) or to the same chemotherapy regimen followed by a single course of RT (56 Gy in 28 fractions) [17,18]. Despite the use of a split-course RT schedule, concurrent therapy was associated with significantly better response rate (84 versus 66 percent), median survival (17 versus 13 months), and two- and five-year survival (35 versus 17 percent and 16 versus 9 percent at two and five years, respectively). 
·	Radiation Therapy Oncology Group (RTOG) trial 9410, a three-arm trial which included 610 patients with unresected stage III disease, compared sequential vinblastine plus cisplatin followed by conventional RT (60 Gy in 30 fractions) with the same regimens administered concurrently [19]. A third study arm combined concurrent chemotherapy with hyperfractionated RT (administered twice daily to a total dose of 69.6 Gy). In a preliminary report, median survival was significantly better with standard RT and concurrent chemotherapy (17 versus 14.6 compared with sequential therapy) as was four-year survival (21 versus 12 percent). Results using hyperfractionated RT plus concurrent chemotherapy were intermediate between these two groups. Although toxicity was increased with the concurrent approach, treatment-related death rates were not increased. 
The optimal regimen for concurrent therapy is not known. The regimen utilized in two phase II studies by the Southwest Oncology Group (SWOG) of two cycles of concurrent cisplatin (50 mg/m2 on days 1, 8, 29, and 36) plus etoposide (50 mg/m2 daily on days 1 to 5, and 29 to 33) with concurrent once daily chest RT to approximately 60 Gy represents a reasonable choice. This was followed by additional cycles of either cisplatin plus etoposide [20] or docetaxel [21].
The efficacy of this regimen was shown in a multicenter phase II trial of 50 patients with pathologically-confirmed stage IIIB disease [22]. With an average follow-up of 52 months, the three and five-year survival rates were 17 and 15 percent, respectively. Treatment was complicated by grade 4 neutropenia in 32 percent, and grade 3 or 4 esophagitis in 12 and 8 percent of patients, respectively. Better results were seen with docetaxel consolidation, but comparative phase III trials will be required to validate those results [21].

1.5.	Conclusion
Concurrent use of chemotherapy and/or RT has become the preferred approach for patients with mediastinal lymph node involvement or unresectable T3 or T4 lesions.
Patients with relatively low-volume mediastinal disease (T2N0=nodal negativ – T3N1=mediastinal negativ; IB – IIIA) are routinely treated surgically and subsequently should be offered an adjuvant chemotherapy if they are fit. Since many patients suffer from a prolonged recovery after surgery those will not benefit from the adjuvant therapy. 
At the moment it is unclear which strategy should be followed: To treat a high number of patients in a neoadjuvant way or to give chemotherapy afterward in a lower number. Nevertheless, to our opinion the major point is, that the highest chance to cure NSCLC is by combining an optimal local with an optimal systemic treatment.
1.6.	Docetaxel
1.6.1.	Name and chemical information
Docetaxel is a semisynthetic taxane derived from a precursor extracted from the needles of the European yew, Taxus baccata.
-	Chemical name: 4-acetoxy-2-benzoyloxy-5, 20 -epoxy-1, 7,10-trihydroxy- 9-oxotax-11 -ene- 13-yl -(2R, 3S)-3-tert-butoxycarbonylamino-2-hydroxy-3-phenylpropionate, trihydrate
-	Structural formula :
		  

-	Molecular formula:	C43H53O14N, 3H2O
-	Molecular weight:	807.9 (anhydrous) - 861.9
-	Appearance :		White powder

-	Solubilities at 20°C:	*	dimethylformamide, acetic acid	 freely soluble
*	methanol	 soluble
*	dichlormethane	 sparingly soluble
*	water	 practically insoluble
1.6.2.	Mechanism of action
Docetaxel has a mechanism of action that is similar to (or may be identical to) paclitaxel. Docetaxel enhances microtubule assembly and inhibits the depolymerization of tubulin. As with paclitaxel, this can lead to bundles of microtubules in the cell, which by blocking cells in the M phase of the cell cycle results in the inability of the cells to divide. This contrasts with the action of other spindle poisons in clinical use such as colchicines or vinca-alkaloids which inhibit tubulin assembly in microtubules. 
Comparing paclitaxel and docetaxel using the "tubulin in vitro assay", the concentration required to provide 50% inhibition of microtubule disassembly (or IC50) for docetaxel is 0.2 µm and for paclitaxel is 0.4 µm.
1.6.3.	Experimental antitumour activity
Docetaxel has been tested against tumours representing a variety of tissue types and behavioral patterns. It is highly active against B16 melanoma. The total log cell kill is 2.5 times greater for docetaxel than for paclitaxel, at equitoxic dosages in this model. Docetaxel is active against three colon tumours: C38, C51 and C26, and causes complete regression of advanced stage colon adenocarcinoma C38. It also causes complete regression of advanced stage pancreatic adenocarcinoma P03.
Docetaxel exhibits cross-resistance to pleiotropic-resistant cell lines.
Docetaxel is considered as a schedule independent drug: Anti-tumour activity correlates with the total dosage that can be administered and dose-splitting does not appreciably change efficacy.
1.6.4.	Animal toxicology
Toxicology studies have been carried out in mice and in dogs, with dogs being the more sensitive species. The main toxic effects of docetaxel, as expected with an anti-mitotic agent which alters microtubule depolymerization, were most evident in tissues with high cell turnover (intestinal, hematopoietic, lymphatic, testicular and hair) or in those where microtubules play an important functional role (neuromotor).
Cumulative and reversible neurotoxicity was seen in mice; haemodynamic effects that might be also due to the solvent used (polysorbate 80) at least in part, and digestive tract lesions were seen in dogs. Both species experienced myelosuppression. Myelosuppression (both species) and digestive tract lesions (dogs) were dose-limiting toxicities.
1.6.5.	Human pharmacokinetic data
The docetaxel kinetic profile is consistent with a three-compartment pharmacokinetic model independent of administration schedule or dose, although the terminal elimination phase cannot always be observed i.e. at low doses and/or for some administration schedules because of the low plasma levels achieved. No evidence of dose-dependence of docetaxel clearance (CL) was observed following either 1-2 hour or 6 hours infusions.
Typical drug exposure following 1 hour infusion of 100 mg/m² is:
- Peak:	 3.67 µg/ml
- AUC:	 4.59 µg.h/ml
Mean pharmacokinetic parameter estimates are:
- t1/2:	 4 min
- t1/2: 	36 min
- t1/2:	 11.1 h
- CL:	 35.3 l/h (21.0 l/h/m²)
- Vss:	 113 l (67.3 l/m²)
1.6.6.	Protein binding
Docetaxel is extensively bound to plasma proteins. In vitro the plasma protein binding is 93-94 % whatever the drug concentration. The main proteins involved are albumin, 1-acid glycoprotein and lipoproteins. A binding of 97.8% was measured in vivo in 3 cancer patients. None of the anticancer drugs studied nor dexamethasone were found to displace docetaxel binding.
1.6.7.	Excretion and metabolism
After infusion of 14C-docetaxel (100 mg/m² in 1 hour), excretion occurs mainly in the feces (75% of the dose) mostly during the first 48 hours post dosing. Urinary excretion accounts for only 5% of the dose. Docetaxel is extensively metabolized and unchanged drug represents a small fraction of the radioactivity excreted. However, most of the circulating radioactivity is accounted for by unchanged docetaxel, and no circulating metabolites could be detected in plasma.
The main metabolic pathway for docetaxel metabolism in humans as in animal species consists of successive oxidations (alcohol, aldehyde, acid) of the tert-butyl ester group on the side chain.
1.6.8.	Safety profile
One thousand three hundred and sixty six patients have been treated at 100 mg/m² in completed North American and European phase II studies (6151 cycles). The data are presented in details in the Investigator's brochure V 8.0 [23] and are briefly summarized below.
1.6.8.1.	Hematological Adverse Events
Neutropenia was the principal toxicity at this dose (92.6% of cycles including 56.4% of grade IV). It was rarely complicated by febrile neutropenia (3% of cycles). The median day to nadir was 8 days and the median duration of grade IV was 7 days. First cycles and subsequent cycles had a similar profile of neutropenia.
Febrile neutropenia was observed in 11.8% of patients.
Anemia was reported in 79.6% of cycles, but was grade III or IV in very few instances (2.7%).
Thrombocytopenia was sporadic (7.8% of patients).
1.6.8.2.	Non-Hematological Adverse Events
Table 1 below gives a summary of the incidence and severity of non-hematological adverse events observed in patients receiving 100 mg/m² docetaxel in the phase II studies. The more clinically significant adverse events are described in greater detail following the table.

Table 1: Incidence and Severity of Main Non-Hematological Adverse Events in Patients receiving 100 mg/m² docetaxel in Phase II studies

Adverse event	Incidence (n=1312)	
	Overall (%)	Grade III-IV (%)	
Allergy	25.9	5.3	
Skin toxicity	56.6	5.9	
Gastro-intestinal			
·	Nausea	40.5	4.0	
·	Vomiting	24.5	3.0	
·	Diarrhea	40.6	4.0	
·	Stomatitis	41.8	5.3	
Neurologic			
·	Sensory	50.0	4.1	
·	Motor	13.8	4.0	
	Overall (%)	Moderate/Severe (%)	
Asthenia	79.0	-	
Nail disorder	27.9	5.9	
Arthralgia	8.6	0.5	
Myalgia	20.0	1.4	
Fluid retention	48.4	8.3	

Corticosteroid premedication, either a 5-day or 3-day regimen, was shown to reduce the incidence and severity of fluid retention, delay its onset and reduce the treatment discontinuation rate due to fluid retention.
A retrospective analysis has been performed to compare the impact of different corticosteroid premedications (no premedication, 5-day corticosteroid and 3-day corticosteroid) on both fluid retention and overall safety in breast cancer patients treated with docetaxel monotherapy 100 mg/m². Three-day and 5-day corticosteroid had similar efficacy in preventing fluid retention. Three-day corticosteroid was associated with less severe infection and stomatitis. Therefore the 3-day corticosteroid premedication is now recommended to all patients treated with docetaxel.
Allergy
Hypersensitivity reactions were generally mild to moderate. The most frequent symptoms observed by patients in decreasing order of frequency were: flushing, dyspnea, chest tightness, pain, facial flushing, hypertension and rash. Shortness of breath and pruritus were less frequent. Severe symptoms such as bronchospasm and hypotension were rare.

Skin reactions
The most frequent cutaneous events observed were, in decreasing order of frequency: erythema, pruritus, dry skin, eruption (macula), swelling, burning and desquamation.
1.6.9.	Human antitumour activity
Docetaxel (Taxotere®) has been approved for treatment of advanced breast cancer in 51 countries and for treatment of locally advanced or metastatic NSCLC after failure of previous chemotherapy. Additionally efficacy has also been demonstrated in the following tumour types: head and neck, ovary, pancreas, gastric, sarcoma and melanoma. Please refer to the Docetaxel Investigator's Brochure V 8.0 for full information on docetaxel efficacy data.
1.6.10.	 Antitumour activity of docetaxel in NSCLC
1.6.10.1.	Efficacy in NSCLC previously treated 
Docetaxel has shown in early phase II studies a relevant anti-tumor activity in patients with NSCLC resistant to cisplatin (CDDP). In the 88 patients treated with docetaxel 100 mg/m2 q. 3 weeks the response rate was 17% with a duration of response of 29 weeks (+17-46) and a 1 year survival of 40% [24,25]. A large phase III study was conducted to compare the antitumor activity and safety of docetaxel single agent either 100 mg/m2 or 75 mg/m2 q 3 weeks to standard regimens such as ifosfamide (I) 2g/m2 day 1, 2, 3, q 3 weeks or vinorelbine (V) 30 mg/m2 day 1, 8 q 3 weeks in patients with NSCLC failing cisplatin. Three hundred and seventy-three patients were randomized; 125 to each docetaxel arm, and 123 to V/I control arm. The selection of V or I treatment was performed according to physician's choice. The overall response rate was significantly higher in D arms with respect to the V/I group (D 100 mg/m2 10.5% p< 0.001; D 75 mg/m² 6.5%, p < 0.036) in which only one patient achieved a PR with an ORR of 0.8%. The overall time to progression (TTP) in D100 was significantly longer (8.4 months vs 7.9 months;p < 0.04) than in the V/I group. A positive trend was also observed for D75 (8.1 months vs 7.9 months) with respect to V/I arm but the difference was not significant. Although there was no advantage in the overall median survival of both docetaxel regimens with respect V/I group (5.6 months in all the treatment arms), the analysis of survival of patients followed up at least 1 year before subsequent chemotherapy was significantly longer for the combined docetaxel arm compared to I/V group (17% alive vs 5% alive p = 0.0012). The benefit achieved in favour of docetaxel arms in terms of objective responses and delayed progression was also evaluated with respect to the quality of life (QOL) using the LCSS questionnaire. This instrument is important since it gives a judgement of patient's subjective well-being while on treatment. There was a significant difference in QOL in favour of the D100 group compared to V/I treatment group (p<0.05) with respect the patient total score fatigue, lung cancer symptoms, and observed total score. Noteworthy, in responders or patients with stable disease while on treatment, there was a clear improvement with both D 100 and D 75 [26].
1.6.10.2.	Efficacy in NSCLC previously untreated
Single-agent docetaxel at 100 mg/m2 administered over 1 hour, once every three weeks has shown significant anti-tumor activity and tolerability in previously untreated advanced NSCLC patients. In four Phase II studies involving 160 such patients, the intent-to-treat analysis revealed a 27% major tumor response rate, median duration of response of 6 months (range 2 - 13+ months), median survival of 9.2 months and a 1-year survival of 39%. Single-agent docetaxel at 75 mg/m² has shown slightly lower response rates, equivalent survival, and a lower incidence of some toxicities [27]. In first-line NSCLC patients, Miller et al. [28] reported tumor response rates of 38% and 25% at 100 and 75 mg/m² respectively, with comparable survivals.
1.6.10.3.	Docetaxel in combination with cisplatin in first line setting
The rationale for the combination of docetaxel and cisplatin is based on the different mechanisms of action, lack of cross-resistance in vitro, and the survival benefit for each agent in this population of patients with minimal overlap of toxicities [29,30].
Phase I combination studies suggest that without growth factor support, 75 mg/m2 of docetaxel followed immediately by 75 mg/m2 of cisplatin, is a manageable regimen. The dose limiting toxicities were haematologic. Approximately half of the cycles were followed by Grade 4 (< 0.5 x 10^9^ cells/L) neutropenia, which was of brief duration, non-cumulative, and rarely complicated by febrile neutropenia. The most frequent non-haematologic toxicities were nausea/vomiting and diarrhea. Pharmacokinetics (PK) of docetaxel and cisplatin were performed during the first cycle of administration of this combination and were consistent with the published results from single-agent studies; suggesting no major pharmacokinetic interaction [31,32].
In a multicenter Phase II study, Belani et al. [32] treated 47 good performance advanced NSCLC patients with 75 mg/m2 of docetaxel (and 5 days of dexamethasone prophylaxis) followed immediately by 75 mg/m2 of cisplatin in three week cycles. The median age was 62 years (range 45-78) and the median number of metastatic sites was two. 229 cycles of chemotherapy were administered. The response rate (1 CR + 9 PRs) was 21.3% and median survival was 10 months. Toxicities included febrile neutropenia in 4 patients and Grade 4 pulmonary toxicity in 2 patients. Severe asthenia was reported in 6 patients, severe fluid retention in one patient and Grade 4 neuropathy in one patient. Other rare Grade 3 toxicities were: nausea, vomiting, neurosensory and neuromotor effects, diarrhea, and stomatitis.
Zalcberg et al used the identical regimen in a multicenter Phase II study of advanced NSCLC patients (except 4 days of dexamethasone prophylaxis). Hydration was provided for 24 hours. Forty-seven patients, two-thirds of whom had metastatic disease, were treated. The median number of cycles received was 4 (range 1-13), with 19 of 180 (11%) of cycles delayed and 30 of 180 (17%) of cycles dose reduced. Thirty-six patients were evaluable for response (evaluability for response required completing two cycles of chemotherapy). The response rate (1 CR + 13 PRs) was 38.9% [95% CI 23.1%, 56.5%], and 36% had stable disease. Median survival was 9.6 months and estimated one year survival was 33%. Significant (Grade 3/4) toxicities included: febrile neutropenia in 13% of patients (two deaths); nausea in 26% of patients, hypotension in 15% of patients, diarrhea in 13% of patients; dyspnea (mainly related to chest infection) in 13% of patients, and abnormalities of magnesium levels in 24% of patients. There was one Grade 4 hypersensitivity reaction. Reasons for discontinuation from trial included cardiac dysrhythmias in 2 patients and fluid retention in 2 patients [33]. A large randomized study proved the high efficacy of the docetaxel/cisplatin combination to be supperior when compared with the control arm vinorelbine/cisplatin. Overall response rate and survival rates were significantly improved, whereas side effects were similar. [34] This study was the basis for the first-line approval of that combination.
1.6.10.4.	Docetaxel with cisplatin in stage IIIA N2 NSCLC
A feasibility study with the combination docetaxel and cisplatin was performed in patients with locally advanced NSCLC [35].
Thirty-four patients with stage IIIA, N2 detected by CT scan in 20 patients and with mediastinoscopy only in 14 patients, received 3 courses of docetaxel 85 mg/m² day 1 and cisplatin 40 mg/m² day 1 and 2 every 21 days. Patients who achieved an objective response or had stable disease after the 3 cycles of chemotherapy, underwent to surgery. 
Sixty four percent of the patients achieved an objective response and a pathological CR was achieved in 12% of the cases. A progression of disease occurred only in 6% of the patients. Downstaging with negative lymph nodes at surgery occurred in 60% of the patients.
The regimen was well tolerated only few adverse events were observed and in general were mild or moderate. Main toxicities included grade 3-4 neutropenia which occurred in 36% and 20% of the cycles respectively, grade 3 diarrhea, grade 3 paresthesia, grade 3 pulmonary toxicitiy and fatigue complicated 4 cycles/patients.
One patient died of gastric bleeding, however it was not possible to state if this SAE was fully related or not to the treatment since the patient was concomitantly treated with steroids. Complete surgical resection was possible in 70% of the patients. No post-operative pulmonary complications were observed. One patient died of heart attack 4 days after surgery.
 
  
1.7.	CISPLATIN / CDDP
1.7.1.	Name and chemical information
-	Cisplatinum/CDDP
-	Chemical name: cis-Diammindichloroplatin 
-	Molecular formula: Cl2H6N2Pt
-	Structural formula:


The biologic activity of platinum compounds was first recognized in 1965. Cisplatinum subsequently entered clinical trials in 1971 [36] and since then has become established as a highly effective drug for treating testicular tumors, ovarian, bladder, head and neck carcinomas and non-small-cell lung cancer.
It acts by binding the monaquo and diaquo derivatives (substitution of chlorine) to the hydroxyl or amine groups of nucleosides and macromolecules and to the thiol groups of proteins. It also forms inter-chain and intra-chain bonds in DNA molecules (acting like a bifunctional alkylating and an intercalating agent) and it has probably an action on DNA repair phenomena.
It binds intensively and irreversibly to plasma proteins (95% at 24 hr), the bound product is inactive [37].
The plasma distribution shows a biphasic decrease in the total plasma levels of Cisplatinum:

t 1/2 (a) = 30 min
t 1/2 (b) = 60 hours

Free Cisplatinum also has a biphasic decay:

t 1/2 (a) = 30 min
t 1/2 (b) = 50 hours

The infusion with mannitol do not induce any pharmacokinetic modifications. 
The elimination is mainly urinary. The circadian rhythm has an effect on the elimination and the toxicity of Cisplatinum in man. The biliary elimination is very small.

1.7.2.	Safety profile
The known complications are [38]:
Acute renal failure related to the platinum concentration in the glomerular ultrafiltrate (mannitol induced osmotic diuresis will decrease the risk of acute renal damage)
Electrolyte disturbances: hyponatremia, hypomagnesemia, hypokalemia.
Neurotoxicity subclinically  present after repeated courses in all patients, and becoming important with high dose regimens.
Ototoxicity related to neuronal damage in the Corti organ.
Nausea and vomiting necessitating antiemetic management.
Haematologic toxicity which is dose-related.
Allergy, hemolytic anemia, rash, CNS leucoencephalopathy.
1.7.3.	Antitumor activity
CDDP is used and registered in Austria for the treatment of several tumor types including NSCLC since a number of years. The compound is well suited for use in combination with docetaxel because of different mechanisms of action and different toxicity profiles. However, as both drugs may cause neurological toxicity, this will need to be carefully monitored.

1.8.	Cetuximab 
1.8.1.	Summary
Cetuximab [39] is a chimeric mouse/human monoclonal antibody of the IgG1 subclass that targets the human epidermal growth factor receptor (EGFR). Cetuximab binds to the extracellular region of the human EGFR and functions as a competitive antagonist that inhibits ligand binding. It can be used to block the EGFR signaling network that is involved in triggering and regulating the malignant growth of many EGFR-positive epithelial tumors.
1.8.2.	Physical, Chemical and Pharmaceutical Properties
Cetuximab is a chimeric mouse/human monoclonal antibody of the IgG1 subclass that targets the human epidermal growth factor receptor (EGFR). It is composed of four polypeptide chains, two identical heavy (gamma, ã)chains consisting of 449 amino acids each, and two identical light (kappa, ê) chains consisting of 214 amino acids each, held together by disulfide and nonconvalent bonds. The light chain and heavy chain subunits contain one and two consensus sequences for N-linked glycosylation, respectively.
Cetuximab is produced by cell culture from a transfected Sp2/0 Ag 14 hybridoma designated as SdER.6.25. Cetuximab is purified from the culture supernatant by a series of affinity and ion exchange chromatographic steps, low pH viral inactivation, and virus filtration. Concentration and buffer exchange is performed by tangential flow filtration. 
Cetuximab drug product (Cetuximab Injection) is a sterile, clear colorless liquid for intravenous (i.v.) administration, which may contain a small amount of easily visible, white amorphous cetuximab particulates. The drug product is formulated as a 5-mg/ml solution. Cetuximab Injection is supplied for clinical use in 100-mg and 500-mg preservative free, single use vials to deliver 20 mL and 100 mL of cetuximab (5 mg/mL). It is possible to dilute Cetuximab up to a maximum value of 0.45 mg/ml with isotonic NaCl solution.

The recommended storage condition for cetuximab is 2 °C to 8 °C with refrigeration. The recommended storage condition is based on data from long-term stability studies conducted with cetuximab. The expiry period in the US is 36 months based on data from completed long-term (36 months) stability studies. Currently, the expiry period in the European Union (EU) is 24 months based on data from on-going stability studies. 

1.8.3.	Toxicology
The potential systemic toxicity after chronic i.v. administration of cetuximab was investigated in primates. Skin toxicity was the major finding observed in a 39-week repeat-dose toxicity study in cynomolgus monkeys at clinically relevant levels. Cetuximab induced severe skin toxicity and lethal complications in monkeys, which exhibited blood levels of approximately 17-fold of those achieved under the standard human treatment regimen. Furthermore, a tendency for impairment of menstrual cycling in treated female monkeys was observed, including increased incidences of irregularity or absence of cycles, when compared to control animals. Preclinical data on genotoxicity and local tolerance after accidental routes of administration revealed no special hazard for humans. 
No formal animal studies have been performed to establish the carcinogenic potential of cetuximab or to determine its effects on male and female fertility. A range-finding study investigating the developmental toxicity of cetuximab has been completed and a definitive study is underway.

1.8.4.	Pharmacokinetics
The pharmacokinetics of cetuximab were studied in single-dose and repeat-dose toxicity studies in rats (14 to 28 days) and monkeys (single-dose pharmacokinetic study and 39 weeks). In single-dose and 4-week toxicology studies in rats, dose-related trends in serum concentration of cetuximab were observed. No significant cumulative effects occurred on circulating levels seen over the 24-day assay period of the 4-week study.

In cynomolgus monkeys, mean peak serum levels across treatment groups were dose dependent, without significant gender differences. In the 39-week toxicology study, steady state levels of cetuximab were reached in Study Week 4. The apparent half-life was similar at the low- (12 mg/kg initial followed by 7.5 mg/kg/week) and mid-dose (38 mg/kg initial followed by 24 mg/kg/week) levels (between 50 and 94 hours), suggesting linear pharmacokinetics within this dose range. No significant gender differences and no accumulation of cetuximab were observed.
In humans, cetuximab administered as monotherapy or in combination with concomitant chemotherapy or radiotherapy exhibits nonlinear pharmacokinetics. The area under the concentration time curve (AUC) increased in a greater than dose proportional manner as the dose increased from 20 to 400 mg/m². Cetuximab clearance (CL) decreased from 0.08 to 0.02 L/h/m² as the dose increased from 20 to 200 mg/m², and at doses > 200 mg/m², it appeared to plateau. The volume of the distribution (Vd) for cetuximab appeared to be independent of dose and approximated the vascular space of 2-3 L/m².
Following a 2-hour infusion of 400 mg/m² of cetuximab, the maximum mean serum concentration (Cmax) was 184 µg/ml (range: 92-327 µg/mL) and the mean elimination half-life was 97 hours (range 41-213 hours). A 1-hour infusion of 250 mg/m² produced a mean Cmax of 140 µg/mL (range 120-170 µg/mL). Following the recommended dose regimen (400 mg/m² initial dose/250 mg/m² weekly dose), cetuximab concentrations reached steady-state levels by the third weekly infusion with mean peak and trough concentrations across studies ranging from 168 to 235 and 41 to 85 µg/mL, respectively. The mean half-life was 114 hours (range 75-188 hours).
A population pharmacokinetic analysis was performed to explore the potential effects of selected covariates including race, gender, age, and hepatic and renal function on cetuximab pharmacokinetics. 
Female patients had a 25% lower intrinsic cetuximab clearance than male patients. Similar efficacy and safety were observed for female and male patients in the clinical trials; therefore, dose modification based on gender is not necessary. None of the other covariates explored appeared to have an impact on cetuximab pharmacokinetics.
Cetuximab has not been studied in pediatric populations.

1.8.5.	Clinical Efficacy 
Cetuximab clinical trials began in 1994. Phase I through III studies have been conducted in the United States, Europe, Asia, Australia, Japan, Brazil, Mexico, and Argentina. Cetuximab has been administered as a single agent or in combination with chemotherapy and/or radiation therapy in clinical trials. Cetuximab's antitumor activity has been observed in patients with colorectal, head and neck, lung, pancreatic, and renal carcinomas, As of the data cut-off date (30-Nov-04) for this IB update, Cetuximab has been approved in the United States, Switzerland, the EU member states, Argentina, Mexico, Chile, and Iceland for the treatment of metastatic colorectal cancer. 
In a multicenter, randomized, controlled Phase II trial in patients with irinotecan-refractory EGFR-positive colorectal cancer (EMR 62 202-007), objective response rates were determined per independent review. For patients treated with cetuximab and irinotecan, the RR was 22.9%. For patients treated with cetuximab monotherapy, the RR was 10.8%. The median duration of response of responders was 5.7 months in the combination arm and 4.2 months in the monotherapy arm.
Cetuximab in combination with irinotecan was studied in a multicenter, single arm, open-label study in patients with EGFR-expressing metastatic colorectal cancer who have progressed following an irinotecan-containing regimen (IMCL CP02-9923). The overall response rate was 15% for the overall population and 12% for the irinotecan-failure population. The median durations of response were 6.5 and 6.7 months, respectively.
Cetuximab studied as a single agent in patients with EGFR-expressing metastatic colorectal cancer who progressed following an irinotecan-containing regimen (IMCL CP02-0141) resulted in an overall response rate of 9% and 14% in the irinotecan-failure group. The median duration of response was 4.2 months for both groups.
In a  completed combination study with cisplatin in patients diagnosed with squamous cell carcinoma of the head and neck (SCCHN) who had progressive disease after cisplatin-containing therapy, a response rate (RR) of 10% was noted (N = 79) (IMCL CP02-9816). In platinum-refractory patients treated with cisplatin or carboplatin in combination with cetuximab, a RR of 10.4% was found according to IRC in EMR 62 202-001 (N = 96). In platinum-refractory patients treated with cetuximab alone, a RR of 12.6% was found according to IRC. In a completed combination study in patients with advanced, newly diagnosed, or locally-regionally recurrent SCCHN (IMCL CP02-9813), a RR of 48% was observed (N = 21). In a Phase III study of cisplatin and cetuximab or placebo in newly diagnosed or recurrent SCCHN incurable with surgery or radiation, a RR of 26.3% was observed in the cisplatin/cetuximab arm (N = 57 patients) versus 9.8% in the control arm (N = 58 patients) (p = 0.0508) (ECOG E5397).
Four combination studies were conducted with cetuximab in patients with non-small cell lung carcinoma (NSCLC). The RR in study IMCL CP02-9925 and IMCL CP02-9932 was 29% in both studies (N = 35; N = 31, respectively). In study IMCL CP02-0036, a RR of 22% was observed with 1 CR (N = 54). Lastly, in EMR 62 202-011, the confirmed RR was 35% (N = 43) in patients treated with combination cetuximab, and 28% (N = 43) in patients treated with only cisplatin/vinorelbine.
The RR in patients with pancreatic cancer treated with cetuximab and gemcitabine (IMCL CP02-9814) was 12.2% (N = 41) and 0% (N = 54) in patients with renal cell carcinoma treated with cetuximab alone (IMCL CP02-9710).

1.8.6. Cetuximab in NSCLC  
Several studies evaluated safety profile, anti-tumor activity and pharmacokinetic effects of cetuximab as first line therapy in advanced NSCLC. In phase I/II studies cetuximab was combined with different platin combinations in advanced NSCLC with evidence of EGFR expression [40,41]. Responses were seen in about 30%, disease stabilization in up to 60%. The most frequent adverse events were acne-like rash, dry skin and fatigue/malaise. Other treatment related adverse effects were anemia, leukopenia, thrombocytopenia and infection. 
A randomized phase II study (LUCAS study) in advanced NSCLC enrolled 86 patients, who received either vinorelbine/cisplatin plus cetuximab or chemotherapy alone [42]. Cetuximab was given in an initial dose of 400 mg/m2, followed by a weekly application of 250 mg/m2. The addition of cetuximab seemed to improve efficacy of cisplatin/vinorelbine with confirmed response rates of 35% vs. 28% and disease control rates of 84% vs. 67%. Typical chemo-associated toxicities were not aggravated by the addition of cetuximab. 
Therefore, based on the LUCAS trial, a randomized phase III study in stage IIIB/IV NSCLC has been initiated using the same combinations (FLEX study (EMR 62202-046) for first-line treatment. In the second-line two further studies were conducted. Testing cetuximab as a monotherapy in 29 patients resulted in a 7% response rate and a disease control rate of 21% [43]. Cetuximab combined with docetaxel increased efficacy and achieved response rates of 22% and a disease control rate of 56% [44].
Summarizing, cetuximab shows considerable activity in advanced NSCLC in the first line setting combined with platinum-based regimens as well as in the second-line setting combined with docetaxel.


1.8.7.	Clinical Safety 
Pooled adverse event (AE) data (preliminary or final) are available for 2315 patients treated with cetuximab alone or in combination with chemotherapy and/or radiation therapy in 21 ImClone, 12 Merck KGaA, 2 BMS, and 1 ECOG studies [39]. 
A total of 99.2% of the patients reported adverse events (AEs). Approximately two-thirds (70.3%) of patients reported at least one Grade 3 or 4 event. Cetuximab-related AEs were observed in 2155 patients (93.1%). The most common composite groupings of adverse events (all grades) related to cetuximab reported in trials include acneiform rash (83.5%), acne like rash (78.5%), and infusion related symptoms (19.7%). Moreover, cetuximab in combination with chemotherapy showed that development of the acneiforme rash was significantly correlated with response to treatment; grade 3 rash may be especially predictive of response [45]. In 2315 patients in the safety database receiving cetuximab on study protocols, 203 patients (8.8%) discontinued treatment because of AEs and another 178 patients /7.7%) because of death on study. A total of 991 (42.8%) patients had serious adverse events (SAEs); of which 279 (12.1%) patients had SAEs deemed possibly, probably or definitely related to cetuximab. 

1.9.	RATIONALE OF THE STUDY
Induction chemotherapy prior to surgery or radiotherapy has proven to be effective. The poor long term results of surgically treated patients with NSCLC have been the reason to test neoadjuvant and adjuvant strategies. Among the aims of neoadjuvant therapy is the early eradication of distant micrometastasis. By delivering cytotoxic drugs there is also the possibility of decreasing the size of tumors, making them more operable in certain patients, an in vivo assessment of tumor responsiveness.
Docetaxel is one of the most active drugs in advanced NSCLC with a one year survival rate following docetaxel monotherapy comparable to that achieved using combinations of older agents. Additionally the results of Betticher et al. with the combination of docetaxel and CDDP report an overall response rate of 66%. Such data justify the further investigation of the neoadjuvant setting with docetaxel and CDDP.  
Cetuximab as a monotherapy has shown limited efficacy in NSCLC. However, the addition of cetuximab seemed to improve efficacy of cisplatin/vinorelbine with confirmed response rates of 35% vs. 28% and disease control rates of 84%vs. 67%. Typical chemo-associated toxicities were not aggravated by the addition of cetuximab.  

The rationale of the study is to combine one of the most effective chemotherapy (DXL/CDDP) with a monoclonal antibody (cetuximab), which could possibly increase the efficacy without increase of major toxicity.

In addition, due to the lack of any immunological surrogate marker for response to antibody-therapy (including cetuximab), a detailed immunological monitoring with special focus on immosuppressive regulatory T-cells /Treg) will be included to identify potential immunological predictors for response to chemo-immunotherapy in NSCLC- patients (for details see Appendix XII).
    


2.	Study objectives
2.1.	 Primary objective
To assess the overall  response rate (ORR) after induction therapy with docetaxel in combination with CDDP and cetuximab in patients with NSCLC stage IB, II, and IIIa. ORR will be determined by the percentage of patients achieving objective response rates (CR + PR) according to the RECIST guidelines (see appendix I). (According to RECIST criteria, confirmation of CR/PR should be performed after 4 weeks. Due to study protocol and to subsequent surgery of target lesions this point can not be fulfilled). 
2.2.	Secondary objectives
·	To evaluate pathological response determined by histological work up of the surgical specimens according to TNM stages.
·	To evaluate the metabolic response determined by PET analysis.
·	To assess overall survival (OS) (median survival time and percentage of 1-year survival). OS is defined as time elapsed from the date of patient inclusion until recorded date of death.
·	To characterize and quantify toxic effects of the scheduled therapy. Safety profile and tolerability will be assessed by recording adverse events, clinically significant laboratory abnormalities, physical examination and vital signs. Toxicities will be evaluated according to the NCI-CTC Toxicity Criteria (see appendix III) and adverse events which are not reported in NCI-CTC will be graded as mild, moderate, severe or life-threatening. All patients who received any of the scheduled therapy will be included in the overall toxicity analysis.
·	To evaluate the immunological response determined by regulatory T-cells and immune activation markers (see Appendix 17.10), to define chemoresistance by pharmacogenomic testing (see Appendix 17.9). 

 


3.	Study design
This is an open label, multicenter, non-randomized phase II study.  
The following diagram summarises the study design: 

Initial staging  (CT/PET/Biopsy/Immunology)
Only resectable NSCLC are included


INDUCTION THERAPY
2 cycles chemotherapy: 
Docetaxel + CDDP + Cetuximab


RESTAGING for operability and evaluation of clinical, metabolic and immunological response
(CT/PET/ /IMMUNOLOGY)


surgery  and correlation of pathological response
(PATHOLOGY)


END OF STUDY  (d  +30 post surgery)


FOLLOW UP: Survival
Further treatment (adjuvant chemotherapy, adjuvant radiotherapy) are at the discretion of the local medical team but are not part of the study
4.	Selection of patients
4.1.	Number of patients
Altogether 40 evaluable patients will be enrolled. 
4.2.	Inclusion criteria
·	Histology and staging of the disease
-	Histological confirmed NSCLC; histology may include: large cell, squamous cell or adenocarcinoma but no SCLC.
-	Anatomically and functionally resectable NSCLC stage IB (T2N0) stage II (T1-2 N1, T3 N0) or stage IIIA (T3 N1)  (see TAKO guidelines 2006, www.tako.or.at). Patients with low tumor volume (this means that patient has to qualify for primary surgical treatment!) and PET-positive N2 and CT negative N2 may also be included after consultation of the local tumor board.
-	Measurable disease according to RECIST criteria
·	General conditions
-	18-80 years.
-	WHO 0-2; life expectancy of more than 3 months
-	Effective contraception for both male and female patients if the risk of conception exists
-	Adequate respiratory function, sufficient for necessary surgical treatment
-	Adequate hematological function (Hb > 10 g/dl, ANC > 2.0 x 10^9^/L, platelets > 100 x 109/L).
-	Adequate renal and hepatic functions: total bilirubin  1.5 x UNL, serum creatinine within normal limits, in case of limit value the creatinine clearance should be > 60 ml/min, ASAT and ALAT < 2.5 x UNL, alkaline phosphatase < 5 x UNL.
·	Initial work-up
-	Complete initial work-up within three weeks prior to first infusion includes chest CT scan, abdominal CT-scan, brain CT scan if indicated, PET-scan, bronchoscopy and mediastinoscopy, pulmonary function. Within 7 days prior to inclusion laboratory investigations and biological work  up.
-	Signed initial consent prior to protocol specific procedures.

4.3.	Exclusion criteria
·	Diagnosis
-	Evidence of brain metastases or other distant metastasis equivalent to stage IV disease
-	History of prior malignancies, except for curatively treated non-melanoma skin cancer or in situ carcinoma of the cervix or other  curatively treated cancer with no evidence of disease for at least five years
-	Other serious concomitant illness or medical condition:
-	Congestive heart failure or angina pectoris, except if medically controlled, history of myocardial infarction within 1 year from study entry, uncontrolled hypertension or arrhythmia
-	History of significant neurological or psychiatric disorders, including dementia or seizure
-	Active infection requiring i.v. antibiotics
-	Active ulcer, unstable diabetes mellitus or other contraindications to corticotherapy
-	Current peripheral neuropathy WHO grade > 2
·	Prior or concurrent therapy
-	Prior chemotherapy or immunotherapy for NSCLC 
-	Prior surgery or radiotherapy for NSCLC
-	Concurrent treatment with other experimental drugs, unapproved medical procedures or other anticancer therapy
-	Concurrent continuous treatment with systemic steroids for antiemetic use, intermittent application is allowed
·	General conditions
-	Pregnant (absence to be confirmed by ß-HCG-test) or lactating patients
-	Patients (M/F) with reproductive potential not implementing adequate contraceptive measurements
-	Participation in other clinical trials with experimental agents or non approved medical procedures during study and within 30 days prior to study entry
-	Psychological, familial, sociological or geographical conditions which do not permit medical follow-up and compliance with the study protocol.
5.	
Study treatments
5.1.	Details of study treatments
5.1.1.	  Induction Therapy
Day 1:		Start of cycle 1: 
Cetuximab 400 mg/m² as a 2-hours i.v. infusion. 1 hour observation time.
Docetaxel (Taxotere®) 75 mg/m² as an 1-hour i.v. infusion, followed immediately by CDDP 
		40 mg/m² as an 1-hour i.v. infusion.
Day 2:		CDDP 40 mg/m² as above.
Day 8:		Cetuximab 250 mg/m² as an 1-hour i.v. infusion. 1 hour observation time.
Day 15:	Cetuximab 250 mg/m² as an 1-hour i.v. infusion. 1 hour observation time.

Day 22:	Start of cylce 2:
Cetuximab 250 mg/m² as an 1-hour i.v. infusion. 1 hour observation time.
Docetaxel (Taxotere®) 75 mg/m² as an 1-hour i.v. infusion, followed immediately by CDDP 
		40 mg/m² as an 1-hour i.v. infusion.
Day 23:	CDDP 40 mg/m² as above. 
Day 29:	Cetuximab 250 mg/m² as an 1-hour i.v. infusion. 1 hour observation time.
Day 36:	Cetuximab 250 mg/m² as an 1-hour i.v. infusion. 1 hour observation time.

Day 43:	End of induction therapy

Within 28 days surgery.


5.1.1.1.	Administration of Cetuximab

The cetuximab dose must always be based on the BSA.
The dosage and administration procedure for cetuximab is as follows:
The first infusion is 400 mg/m². As prophylaxis to reduce the risk of an allergic/hypersensitivity reaction, it is mandatory to pretreat the patient with an appropriate antihistamine.

It is administered as follows: The volume is administered over 120 minutes (maximum rate of 5mL/min). Saline solution (0.9%) is used to flush the line at the end of the infusion. Vital signs should be checked before, during, immediately after and 1 hour after the end of the infusion. 

The subsequent dose given every 7 days is 250 mg/m². It is recommended to pretreat the patient with an appropriate antihistamine. For treatment modifications due to adverse events, see section 5.6.

It is administered as follows: The volume is administered over 60 minutes (maximum rate of 5 mL/min). Saline solution (0.9%) is used to flush the line at the end of the infusion. Careful observation of the patient (including monitoring of vital signs as above) is required until 1 hour after the infusion to monitor the possible occurrence of AEs (specifically allergic/hypersensitivity reactions).  

A physician must be present until the complete infusion has been given and 1 hour after the end of the infusion has elapsed.

If chemotherapy is delayed, administration of cetuximab every 7 days is continued. 

5.1.2.	  Surgery
Surgery should be performed within 28 days after end of induction therapy and/ or complete recovery of toxic side effects.
Lobectomy or pneumectomy with lymphnode dissection should be performed according to TAKO guidelines. Tissue samples for subsequent scientific tests (see appendices 17.9. and 17.10.) should be obtained (see 7.1.1.3). But it has to be guaranteed that there is sufficient tissue for pathohistological diagnosis. Furthermore cutaneous biopsies should be taken during surgery (see 5.6.1.4.).

If surgery cannot be performed, patient has to be taken off study. In that case further treatment, mostly radiochemotherapy will be performed according to the recommendation of the interdisciplinary team.

5.1.3.	  End of Study – Follow up
The clinical study ends with the “end of study visit” which should be performed between day 30 and 45 post surgery and should include all the tests listed in point 7.2.5.
Follow-up will be documented. Further treatment (adjuvant chemotherapy, adjuvant radiotherapy) are at the discretion of the local medical team but are not part of the study.
 		
5.2.	Patients registration
All patients, who gave informed consent have to be announced to the study centre immediately. There, patients will be numbered consecutively in the order in which they enter the study.    
Patients withdrawn from the study retain their patient number, if already given.  New patients must always be allotted a new patient number. This applies also for patients who, after consenting to the study, decide not to participate before administration of the first dose of study (i.e. the next patient enrolled is given the next number). 
5.3.	Blinding 
According to the study design, randomization or blinding is not applicable.  
  


5.4.	PREMEDICATION  / PROPHYLACTIC MEDICATION

5.4.1.	Antihistamines
Dibondrin (1 ampule) in 100 ml 0,9% NaCl prior to cetuximab to prevent the onset of hypersensitive reactions. 
5.4.2.	Corticosteroids	 
The following premedication will be given to all patients starting prior to each docetaxel infusion in order to prevent the onset of hypersensitivity reactions and to reduce and delay the occurrence of skin toxicity and fluid retention related to docetaxel: Dexamethasone 8 mg p.o. will be given day –1, day 0, day +1, and dexamethasone 8 mg i.v. or p.o. 1 hour prior to docetaxel infusion.

Prior to first cetuximab infusion: SoluDac 150-250mg (or equivalent, e.g. Dexabene 20-35mg) to prevent an anaphylactic shock.

5.4.3.	Antiemetics 
All patients should receive a prophylactic antiemetic premedication. 5-HT3 antagonist prior to start of each docetaxel infusion is mandatory. Additional antiemetic treatment is allowed.
5.4.4.	Antibiotics
Prophylactic antibiotic therapy is allowed, but not recommended.
5.4.5.	Recombinant Granulocyte Colony Stimulating Factor (GCSF)
Prophylactic GCSF is mandatory in case of febrile neutropenia, neutropenia grade 4, documented infection or delayed recovery of absolute neutrophil count at day 21 during the previous cycle.
5.4.6.	Hyperhydration
During induction chemotherapy with docetaxel/CDDP hyperhydration as prophylactic treatment for CDDP - nephrotoxicity can be given according to the standard regimen of the institution (a minimum of 2000 ml of hydration is required.
   
5.5.	CONCOMITANT TREATMENTS
Ancillary treatments for toxicities or adverse events will be given as medically indicated (antiemetics, antiallergic, etc.). All concomitant treatments and interventions will be documented in the case report form. Additional use of St. John's wort (“Johanniskraut”) is not possible. 

5.6.	DOSE MODIFICATIONS AND ACTIONS FOLLOWING TO TOXICITIES
Doses will be modified in case of severe hematological and/or non-hematological toxicities. Dose adjustments are to be made according to the system showing the greatest degree of toxicity. Toxicities will be graded using the NCI-CTC  toxicity criteria (see appendix III).
When used as single agent, the major toxic effect of docetaxel which limits dose is neutropenia. Other toxic effects which may be seen include anaphylactoid type reactions and cutaneous reactions, hypersensitivity reactions, digestive tract toxicities (nausea, vomiting, oral mucositis, diarrhea), reversible paresthesias, alopecia, asthenia, and mild local venous reactions (phlebitis) at site of injection and fluid retention/edema. The guidelines which follow outline dose adjustments for several of these toxic effects.

The principal dose-limiting toxicity of CDDP is acute renal failure related to the platinum concentration in the glomerular ultrafiltrate (mannitol induced osmotic diuresis will decrease the risk of acute renal damage). Other toxic effects include electrolyte disturbances: hyponatremia, hypomagnesemia, hypokalemia; furthermore neurotoxicity subclinically present after repeated courses in all patients, and becoming important with high dose regimens, ototoxicity related to neuronal damage in the Corti organ, nausea and vomiting necessitating antiemetic management, hematological toxicity which is dose-related, allergy, hemolytic anemia, rash and CNS leucoencephalopathy.

The most common adverse events associated with cetuximab administration are skin reactions, particularly rash and fatigue. No specific therapy is recommended. General recommendations for prophylaxis  and therapy of skin toxicity are summarized by Perez-Soler (Oncologist 2005: 10:345-356): prevent dehydration of skin, prevent intensive sun exposure, use creams and emollients to prevent and alleviate skin dryness, avoid acne medication, topical steroids if indicated by the dermatologist may be used. In case of  secondary infection, topical and systemic antibiotic therapy might be considered.
Dose adjustments for toxicity should be made according to guidelines which follow. If a patient experiences several toxicities and there are conflicting recommendations, please follow the most conservative dose adjustment recommended. Treatment may be delayed no more than 2 weeks to allow recovery from toxicity. If treatment must be delayed longer than 2 weeks, patient should be removed from protocol treatment. In case of chemotherapy associated toxicities cetuximab should be continued. 

5.6.1.	Toxicities from Docetaxel and Cisplatin
In case of severe toxicity throughout the first cycle the start of the second cycle may be delayed up to two weeks. After side effects have resolved dose reduction for the second cycle is recommended. 

5.6.1.1.	Myelosuppression and its complications

Toxicity	Action to be taken for second cycle 	
Febrile Neutropenia (Grade 4 neutropenia – ANC < 0.5 x10^9^/l – and grade 2 fever – temperature > 38.1°C) and/or Documented Infection	The first episode will result in the addition of prophylactic GCSF to the second cycle
  	
Neutropenia grade 4 (ANC < 0.5 x10^9^/l) > 7 days	Docetaxel (and CDDP) dose reduced one level as outlined above and addition of prophylactic GCSF to the second cycle	
Neutropenia grade 4 (ANC < 0.5 x10^9^/l) or delayed recovery on day 21 (ANC 1,0 – 1,5 x10^9^/l )	Addition of prophylactic GCSF to the second cycle	
Thrombocytopenia grade 4 (platelets < 25 x109/l)	Docetaxel (and CDDP) dose reduced one level    	

Delayed recovery of blood counts on day 21 of cycle:
Neutrophils < 1.0 x10^9^/l  and/or
Platelets < 100 x109/l 	
Delay of next cycle by a maximum of 2 weeks. If recovery within 2 weeks, prophylactic GCSF should be given during the second cycle. Docetaxel (and CDDP) dose of the next cycle should be reduced to 75%. If no recovery within 2 weeks, patient will go off protocol therapy. 	
5.6.1.2.	Peripheral neuropathy

Toxicity	Action to be taken for subsequent cycles	
> Grade 3	Patient will go off protocol therapy	
Grade 2	Docetaxel (and CDDP) dose reduced one level for next cycle as outlined above	
Grade 1	No action	
5.6.1.3.	Hypersensitivity reactions

Toxicity	Action to be taken for subsequent cycles	
Grade 4	Patient will go off protocol	
Grade 2, 3	Interrupt docetaxel (and CDDP) infusion
Give diphenhydramine  50 mg i.v. with or without dexamethasone 10 mg i.v.
Restart infusion after recovery of symptoms at a slower infusion rate
Depending on the intensity of the reaction additional premedication with antihistamines may be given for the next cycle	
Grade 1	Consider decreasing infusion rate	

5.6.1.4.	Cutaneous reactions
To better understand the skin toxicity as a potential predictive marker, specific skin analysis using proteomics are planned. In the case of grade 2 or more, two skin biopsies should be taken, one from an area with macroscopic normal signs (control) and one with significant acneiforme rash. Samples should be sent immediately in PBS or NaCl 0.9% to the laboratory for “Molekulare Zellbiologie” – Innere Medizin, Innsbruck (Dr. Pircher: Tel. 0512-504-25612).

Toxicity	Action to be taken for subsequent cycles	
> Grade 3	Delay next cycle until grade < 2 for a maximum of 2 weeks, then reduce dose of docetaxel (and CDDP) by 1 level.
If no recovery until grade < 2 within 2 weeks, patient will go off protocol therapy	
Grade 1, 2	No action	
5.6.1.5.	Fluid retention
Patients developing edema or other signs of fluid retention should be treated with  diuretics.  


5.6.1.6.	Diarrhea

Toxicity	Action to be taken for subsequent cycles	
> Grade 3	Docetaxel (and CDDP) dose reduced one level for next cycle as outlined above
For next cycle add loperamide as prophylactic treatment
If diarrhoea cannot be reduced by prophylactic treatment patient should go off protocol therapy	
Grade 2	For next cycle add loperamide as prophylactic treatment	

5.6.1.7.	Bilirubin and impaired liver function
 

Toxicity

ALAT / ASAT   and	

Alkaline phosphatase 	
Action to be taken for subsequent cycles	
> 2.5 x UNL to
 < 5 x UNL	<  5 x UNL	Docetaxel (and CDDP) dose reduced one level for next cycle  	
> 5 x UNL	> 5 x UNL	Delay next cycle until recovery to above figures for a maximum of 2 weeks, then reduce dose of docetaxel (and CDDP) 1 level. 
If no recovery patient should go off protocol therapy 	

Bilirubin			
> 1.5 x UNL		Delay next cycle for a maximum of 2 weeks. If no recovery, the patient will go off protocol therapy.
	
5.6.1.8.	Other toxicities
Other toxic effects caused by docetaxel (and/or CDDP) should be managed symptomatically. For grade 3 toxicities next cycle should be delayed by a maximum of 2 weeks until recovery < grade 1. If there is no recovery and in case of grade 4 toxicities, patient should go off protocol therapy.
5.6.1.9.	Nail changes
Nail changes will not motivate any dose modification.
5.6.1.10.	Treatment adjustment for cetuximab related allergic/hypersensitivity reactions

CTC Grade Allergic/hypersensitivity reaction	Treatment	
Grade 1: 
Transient rash, drug fever <38°C	Decrease the cetuximab infusion rate by 50% and monitor closely for any worsening.
The total infusion time for cetuximab should not exceed 240 minutes	
Grade 2: 
Urticaria, drug fever >38°C and/or asymptomatic bronchospasm        	Stop cetuximab infusion
Administer bronchodilators, oxygen, etc. as medically indicated
Resume infusion at 50% of previous rate once allergic/hypersensitivity reaction has resolved or decreased to grade 1 in severity, and monitor closely for any worsening. 	
Grade 3 or Grade 4
Grade 3: Symptomatic bronchospasm, requiring parenteral medication, with or without urticaria; hypersensitivity-related edema, angioedema


Grade 4: Anaphylaxis	Stop cetuximab infusion immediately and disconnect infusion tubing from the patient.
Administer epinephrine, bronchodilators, antihistamines, glucocorticoids, intravenous fluids, vasopressor agents, oxygen, etc., as medically indicated.

Patients have to be withdrawn immediately from treatment and must not receive any further cetuximab treatment	

Resumption of treatment following allergic/hypersensitivity reactions:
Once the cetuximab infusion rate has been decreased due to an allergic/hypersensitivity reaction, it must remain decreased for all subsequent infusions. If the patient has a second allergic/hypersensitivity reaction on the slower infusion rate, the infusion should be stopped and the patient should be removed from the study. If a patient experiences a grade 3 or 4 allergic/hypersensitivity reaction at any time, cetuximab must be discontinued. 
5.7.1.11 Skin toxicity 
If a patient experiences grade 3 skin toxicity, cetuximab therapy may be delayed for up to 14 days without changing the dose level. The investigator should also consider concomitant treatment with topical and oral antibiotics; topical corticosteroids are not recommended. If the toxicity resolves to grade 2 or less by the following treatment period, treatment may be resumed. If grade 3 skin toxicity occurs for a second  time, cetuximab therapy may again be delayed for up to 14 days with concomitant dose reductions to 200 mg/m² and then 150 mg/m². Cetuximab dose reductions are permanent. Patients must discontinue cetuximab if more than 2 consecutive infusions are withheld or grade 3 skin toxicity occurs for a third time despite appropriate dose reduction. 
 
 
 

6.	Prior and concomitant illnesses and treatments
6.1.	Prior and concomitant illnesses
Additional illnesses present at the time informed consent is given are regarded as concomitant illnesses and will be documented on the appropriate pages of the case report form.
Illnesses first occurring or detected during the study, and worsening of a concomitant illness during the study, are to be regarded as adverse events and must be documented as such in the case report form (see Section 8.   Adverse events).
6.2.	Prior and concomitant treatments
All additional treatments being taken by the patients on entry to the study or at any time during the study are regarded as concomitant treatments and must be documented on the appropriate pages of the case report form.


7.	Study procedures and schedule
7.1.	Overview of data collection
A flow-chart of the examinations is provided on page on page 4 of  this clinical study report.

7.1.1.	Criteria for evaluation of efficacy
7.1.1.1.	Overall Response Rate	(ORR)
The primary efficacy variable, defined as the ORR (complete plus partial response) to docetaxel in combination with CDDP and cetuximab, will be determined by the percentage of patients achieving objective response rates according to the RECIST guidelines (see appendix I). ). All patients receiving at least one cycle of chemotherapy as induction will be considered as evaluable for efficacy. Response will be evaluated according to the RECIST criteria except for the criterium “4 weeks confirmation”, since no confirmation of response after 4 weeks will be available. The date of response is defined as the date when the first determination showing tumor response is made.

7.1.1.2.	Pathological response
The pathological response will be defined by histological examination of the surgical specimens based on the TNM classification.
7.1.1.3.	Immunological  response – scientific analysis
The immunological response will be defined by subsequent analysis of regulatory T-cells. Therefore serum probes (5 mL) are taken at baseline, weekly during chemotherapy and at the end of induction. Heparinized blood is taken at baseline (40 mL), every cycle (20 mL), at the end of induction (20 mL) and at the end of treatment (20 mL) (see Appendix 17.10.). This only applies to centers in Tyrol (Innsbruck, Zams, Natters, Kufstein)
Immunohistochemical analysis of fresh frozen tumour probes. This analysis is optional and should be performed, if sufficient tumor tissue can be assessed by surgery (pathological diagnosis should not be negatively influenced). For internal control, a macroscopic normal tissue probe is assessed by the surgeon out  of the surgical specimen. Immediately after surgery the samples should be sent in PBS/NaCl 0.9% to the laboratory for “Molekulare Zellbiologie”, Innere Medizin, Innsbruck (Dr. Pircher: Tel. 0512-504-25612, oder Prof. Hilbe).
For testing of chemoresistance and genetic alterations, serum probes are analysed at baseline, week three (= start of cycle 2), end of incuction, end of treatment (see Appendix 17.10.). This only applies to centers in Tyrol (Innsbruck, Zams, Natters, Kufstein)

7.1.1.4.	Metabolic response
The metabolic response will be defined by PET scans at baseline and at the end of induction treatment. 
7.1.1.5.	Overall Survival (OS)
OS is defined as time elapsed from the date of patient inclusion until recorded date of death and will be assessed for all patients, who received at least 1 cycle of induction chemotherapy. 

7.1.2.	Criteria for evaluation of safety
All adverse events, toxicities and laboratory abnormal values will be documented in the case report form and evaluated for assessment of safety. All clinical signs and symptoms and laboratory toxicities will be assessed at baseline, after each cycle of induction chemotherapy, at end of surgery, postoperative radiation or radio-chemotherapy and after each cycle of adjuvant chemotherapy . They will be graded according to the NCI-CTC Toxicity Criteria (see appendix III) .
 
7.1.3.	Definitions for evaluability
7.1.3.1.	Eligibility
All patients that fulfill the inclusion and exclusion criteria are defined as eligible. 
7.1.3.2.	Evaluability for safety
All eligible and ineligible patients that received one dose of study medication and having at least one post baseline safety evaluation will be evaluable for safety and toxicity.
7.1.3.3.	Evaluability for response and efficacy
Patients must fulfill following criteria to be analyzed and evaluated for response and efficacy:
For primary efficacy parameter (ORR):
·	Intent-to-treat (ITT)-population: please refer to chapter 11.1.2.
·	Per protocol (PP)-population: please refer to chapter 11.1.2.
For secondary efficacy parameters (TTP, pathological response, metabolic response, immunological response):
·	Patients must be eligible.
·	Patients must have received a minimum of 1 cycle  of treatment with at least one follow-up tumour assessment (all lesions assessed with the same method of measurement as baseline).  
·	Patients must not have experienced any major protocol deviation on study (in particular must not have received any concomitant anticancer therapy).
7.2.	Description of study visits
7.2.1.	Baseline Examination (Study Entry)
·	Inclusion/exclusion criteria.
·	Informed consent.
·	Demographics and baseline information:
Date of birth, gender, weight, height, BSA, date of diagnosis
·	Tumor staging and assessment
·	Vital Signs: Weight, blood pressure, heart rate, body temperature
·	ECG
·	Preexisting medical conditions, signs and symptoms
·	Concomitant therapy
·	Medical/surgical history.
·	Physical and neurological examination 
·	Radiology: Chest CT scan and 
abdominal CT scan  
·	Brain CT-scan: if indicated
·	PET-scan 
·	Bronchoscopy
·	Mediastinoscopy, optional selective puncture of mediastinal lymphnodes (≥ 10mm) according to local standards (should be performed after PET and  CT examinations.).
·	Lung-function-tests
·	Pregnancy testing (for women of childbearing potential only).
·	Hematology: WBC, neutrophils, lymphocytes, hemoglobin, platelets. 
·	Biochemistry: total protein, serum creatinine, creatinine clearance (if indicated), serum electrolytes (K, Na, Cl, Mg), alkaline phosphatase, ASAT (GOT), ALAT (GPT), total bilirubin, GT, LDH, CRP, tumor marker (optional) (CA 19.9, Cyfra, NSE)
·	Serum and plasma samples for immunological testing: This only applies to centers in Tyrol (Innsbruck, Zams, Natters, Kufstein)

7.2.2.	During induction therapy  
·	Physical and neurological examination (every cycle). 
·	Vital sign measurements (every cycle).
·	Hematology (every week) 
·	Biochemistry (every cycle, tumour marker optional)
·	Serum and plasma samples for immunologic testing: This only applies to centers in Tyrol (Innsbruck, Zams, Natters, Kufstein)
·	ECG (if indicated). 
·	Symptoms / adverse events: every cycle
·	Concomitant therapy: every cycle
·	Pregnancy test: every month
·	In case of skin toxicities NCI CTC ≥2 biopsies of acneiforme rash and control recommended (for details see 5.6.1.4.)


7.2.3.	End of induction therapy/before surgery
·	Physical and neurological examination  
·	Vital sign measurements  
·	Hematology  
·	Biochemistry (tumour marker optional).
·	Serum and plasma samples for immunologic testing: This only applies to centers in Tyrol (Innsbruck, Zams, Natters, Kufstein)
·	ECG 
·	Chest CT scan and
·	abdominal CT scan  
·	Brain CT-scan: optional
·	PET-scan 
·	Bronchoscopy: optional
·	Lung-function-tests 
·	Symptoms / adverse events: every cycle
·	Concomitant therapy 
·	Pregnancy test: every month
·	During surgery: 
1.	Tissue samples of tumour and macroscopic normal lung  see 7.1.1.3
2.	Samples of normal skin from surgical cut (see 5.6.1.4.)
      
7.2.4.	End of surgery  
·	Physical examination. 
·	Vital sign measurements.
·	Haematology  
·	Biochemistry ( tumor marker optional)
·	Serum and plasma samples for immunological testing: This only applies to centers in Tyrol (Innsbruck, Zams, Natters, Kufstein)
·	ECG (if indicated).
·	Lung-function-tests: if indicated
·	Symptoms / adverse events 
·	Concomitant therapy 
·	Pregnancy test: every month

7.2.5.	End of treatment (day +30 to day +45 post surgery)
·	Physical and neurological examination. 
·	Vital sign measurements 
·	Hematology 
·	Biochemistry (tumour marker optional) 
·	Serum and plasma samples for immunological testing: This only applies to centers in Tyrol (Innsbruck, Zams, Natters, Kufstein)
·	ECG (if indicated).
·	Radiology
·	Lung-function-tests: if indicated
·	Symptoms / adverse events
·	Concomitant therapy
·	Pregnancy test 

7.2.6.	Follow up
Follow up examinations will be done according to local standards.


8.	Adverse events
8.1.	DEFINITIONS 
An ADVERSE EVENT is any symptom, sign, illness or experience which develops or worsens in severity during the course of the study. Intercurrent illnesses or injuries should be regarded as adverse events.  Abnormal results of diagnostic procedures are considered to be adverse events if the abnormality:
	- results in study withdrawal
	- is associated with a serious adverse event
	- is associated with clinical signs or symptoms
	- leads to treatment or to further diagnostic tests
	- is considered by the investigator to be of clinical significance
Adverse events are classified as either serious or non-serious. 
A SERIOUS ADVERSE EVENT is defined as follows:
	- Subject died
	- Life-threatening
	- Hospitalization/prolongation
	- Persistent/significant disability
	- Congenital abnormality
	- Important medical event
"Life-threatening" means that the patient was at immediate risk of death from the event as it occurred. It does not include an event that, had it occurred in a more serious form, might have caused death.
"Requires inpatient hospitalization" should be defined as hospital admission required for treatment of the adverse event. 
No serious Adverse Events are:
­	Hospital admission for scheduled elective surgery (e.g. port-a-cath)
­	Admission for routine chemotherapy (outpatient ward not in all county hospitals available)
­	Prolongation of hospitalization due to administrative/ social reasons (discharge not possible during the weekend) or patients wish. 	

“Important medical events” are those which may not be immediately life-threatening, but are clearly of major clinical significance. They may jeopardize the subject, and may require intervention to prevent one of the other serious outcomes. Cancer and drug overdose or abuse will normally be considered as serious.
All adverse events which do not meet any of the criteria for serious should be regarded as non-serious adverse events. A non-serious adverse event can though be severe.
8.2.	RECORDING OF ADVERSE EVENTS
At each contact with the subject, the investigator must seek information on adverse events by specific questioning and, as appropriate, by examination.  Information on all adverse events should be recorded immediately in the case report form. All clearly related signs, symptoms and abnormal diagnostic procedures should be grouped together and recorded as a single diagnosis in the CRFs. The component parts of the diagnosis may be listed for verification.
All adverse events occurring during the study therapy must be recorded.  The clinical course of each event should be followed until resolution, stabilization or until it has been determined that study treatment or participation is not the cause.  Serious adverse events which are still ongoing at the end of the treatment period must be followed up to determine the final outcome.
Any serious adverse event which occurs after the study period and is considered to be possibly related to study treatment or study participation should be recorded and reported immediately.

8.3.	Reporting of serious adverse events
The following forms have to be filled in:
1.	Serious Adverse Report Form
2.	“Meldung von schwerwiegenden unerwünschten Ereignissen im Rahmen einer klinischen Prüfung (Meldebogen des Bundesministeriums)”

Both reports should be faxed to the STUDY CENTER:
Univ.-Prof. Dr. Wolfgang HILBE
 Fax:	0512 -504 -23431

All serious adverse events during the study treatment period, whether or not considered to be related to study treatment, must be reported to the study center within 24 hours or, at the latest, on the following working day using the Serious Adverse Event Report Form (see appendix VI).  
According to legislation the official form of Serious Adverse Events (“Meldung von schwerwiegenden unerwünschten Ereignissen im Rahmen einer klinischen Prüfung) also has to be faxed immediately (within 24 hours) by the treating physician (investigator or designees) to the study center, which will inform the Ministery of Health (“Bundesministerium für Soziale Sicherheit und Generationen”) and the ethics committee.
At the time of the initial report, the following information should be provided if possible: study, centre and subject number; the study phase during which the event occurred; a description of the event, date of onset and current status; the start date of treatment, whether treatment has been discontinued, the reason why the event is classified as serious; the investigator's current assessment of the association between the event and study treatment.
Within the following 48 hours, the investigator must provide further information on each serious adverse event.  This should include copies of the completed adverse event and Supplementary Serious Adverse Event forms, and any other diagnostic information which will assist the understanding of the event.  Significant new information on ongoing serious adverse events should be provided promptly to the Study Center. 
The Study Center will then forward all reports to the national health authorities and to the ethics committee.
For safety reasons, all the subinvestigators and the companies Sanofi-Aventis and Merck will be informed on all the SAEs and SUSARS (suspected unexpected serious adverse reactions) by fax or email.

8.4.	ANNUAL SAFETY REPORTS
According to the guidelines of the European Commission (SAE reports) annual safety reports will be sent to the responsible Ministry of Health and the Ethics Committee throughout the clinical trial. 
The report will include:
Analysis of the subjects' safety
A line listing of all suspected serious adverse reactions (including all suspected unexpected serious adverse reactions = SUSARs).

8.5.	Subject removal from study therapy due to adverse events
Any subject who experiences an adverse event may be withdrawn at any time from the study at the discretion of the investigator. 
If a subject is withdrawn because of an adverse event the STUDY CENTER should be informed without delay and the case report form should be completed. Nevertheless the patient should be followed up until death or lost-to-follow-up.


9.	Withdrawals
9.1.	Withdrawal of patients
As far as possible, all examinations scheduled for the “end of induction therapy” check-up examination should be performed on patients who are withdrawn from study medication prematurely. All efforts will be made to perform these examinations within 72 hours of withdrawal.  With respect to additional check-up examinations, patients should continue - as far as possible - with the study schedule as planned. 

The patient may be withdrawn from study medication for the following reasons:
	At his own request or at the request of their legally authorised representative
	If, in the investigator's opinion, continuation in the study would be detrimental to the patient's well-being
	At the specific request of the sponsor
The patient must be withdrawn from study medication under the following circumstances:
	Pregnancy (Every attempt must be made to follow up patients who become pregnant to determine the outcome of the pregnancy.)
	Deterioration of the clinical condition or delayed response. 
	The occurrence of life threatening adverse events that may be related to study medication
In all cases, the reason for withdrawal must be recorded in the case report form and in the patient's medical records.  The patient must be followed up to establish whether the reason was an adverse event, and, if so, this must be reported in accordance with the procedures in Section 8.   Adverse events.
As far as possible, all examinations scheduled for the end of induction chemotherapy must be performed on all patients who receive study medication but do not complete the study according to protocol.
The investigator must make every effort to contact patients lost to follow-up or to get detailed information why they were lost.


10.	Statistical procedures
10.1.	 POPULATION FOR ANALYSIS
10.1.1.	Safety analysis
All patients included in the study, receiving at least one dose of study medication, and having at least one post baseline safety evaluation, will be evaluated for safety/tolerability of study medication. 
10.1.2.	Efficacy analysis (for primary efficacy parameter ORR)
·	Intent-to-treat (ITT)
All patients included in the study, receiving at least one cycle of induction chemotherapy, with a baseline and at least one post baseline evaluation measurement of the primary efficacy parameter, will enter intention to treat analysis. Patients with progressive disease during the first cycle will be classified as early progressors.
·	Per protocol (PP)
All eligible patients available for intention to treat analysis, who completed the planned duration of treatment with induction chemotherapy, were compliant to the treatment regimen, who had a valid final efficacy evaluation, and did not violate the protocol in any way liable to influence efficacy outcome, are valid for evaluation according to the primary efficacy parameter.
10.1.3.	 Analysis of demographic data and patient baseline data
Demographic and background information will be summarized and displayed using descriptive statistical techniques. For categorical variables frequency tables will be presented, for continuous variables descriptive statistics such as mean, median, standard deviation, minimum, maximum etc. will be tabulated.
10.1.4.	Descriptive analysis
All CRF data will be listed case wise and separately for each variable. For categorical variables frequency tables will be presented, for continuous variables descriptive statistics such as mean, standard deviation, minimum, maximum etc. will be tabulated.
10.1.5.	Efficacy analysis
The primary efficacy parameter is defined as the overall response rate (complete plus partial response) to docetaxel in combination with cisplatin and cetuximab after induction therapy and will be determined by the percentage of patients achieving objective response rates according to the RECIST guidelines except for the criteria “4 weeks confirmation”, since no confirmation of response after 4 weeks will be available.
Efficacy analysis will be performed using the ITT population and for the PP population.
The secondary efficacy parameters to be analyzed are:
Pathological response
Metabolic response
Immunological response
overall survival (median survival time and percentage of 1-year survival)
Safety profile and tolerability
These parameters will be analyzed in a descriptive way, seperated for drop-out patients after induction therapy and patients who underwent all scheduled therapies, including adjuvant chemotherapy. 

10.1.6.	Assessment of safety and tolerance
Safety assessments will be performed at baseline, after each cycle of induction chemotherapy, at end of surgery and at the end of treatment. They will be graded according to the NCI-CTC Toxicity Criteria (see appendix III).
Safety will be evaluated in terms of physical examinations findings, vital signs, clinical laboratory analysis and adverse events findings. For all safety parameters the results at each time point will be compared with the results at baseline. Differences will be examined. Descriptive statistics of the vital sign parameters and their differences from baseline will be tabulated for each time point.
10.1.6.1.	Physical examinations
All abnormalities discovered during a physical examination will be listed for each treatment group, by subject. The incidence of newly-occurring abnormalities will be tabulated.
A contingency table summarizing the status of baseline physical examination findings will be presented.
10.1.6.2.	Vital signs
Vital sign data will be listed by subject and treatment group, and any newly occurring changes outside the expanded reference range from baseline will be flagged.
Mean changes from baseline for vital sign data will be summarized and presented graphically for each treatment.
Shift tables will be presented, utilizing the expanded reference ranges. These tables will summarize the change from baseline status for a subject's worst value, defined as that of greatest pathological significance, observed during the study for a given variable.
Subjects with notable abnormal values will be identified and listed separately along with their values. 
10.1.6.3.	Laboratory analyses
Laboratory values outside normal ranges will be flagged and their frequency tabulated. Systematic changes from baseline values will be investigated using frequency tables. Moreover, any vital signs or laboratory parameter showing an abnormal behavior will be analyzed graphically as a function of time with specific emphasis on outlier identification.
10.1.6.4.	Adverse events
All information pertaining to adverse events (whether reported, elicited or observed) noted during the study will be displayed per treatment group, sorted according to patient and type of effect, using body system and preferred term. The resulting list will include:

	Patient ID
	Type of adverse event
	Day of occurrence
	Duration
	Intensity
	Relation to test medication
	Consequences
	Outcome
10.1.7.	Stopping rule
If a total number of 20 non- responding patients is reached in this clinical trial an early termination of the study should be considered since the anticipated efficacy can not be reached any more.
10.2.	 Interim analysis
No interim analysis is planned for this study.
10.3.	 Sample size justification
The primary endpoint of this study is to assess the response rate following induction chemotherapy with docetaxel in combination with CDDP and Cetuximab. The sample size is 40, a two-sided 95,0% confidence interval for a single proportion using the large sample normal approximation will extend 0.147 from the observed proportion for an expected proportion of 0.660.


11.	   Ethical and legal aspects
11. 1.   Good clinical practice
The procedures set out in this study protocol, pertaining to the conduct, evaluation, and documentation of this study, are designed to ensure that the sponsor and investigator abide by good clinical practice (GCP) as described in 21 US Code of Federal Regulations, Parts 50, 56, and 312, as well as in the ICH Harmonised Tripartite Guidelines Topic E 6:  “Guideline for Good Clinical Practice.”  Compliance with these regulations also constitutes compliance with the ethical principles described in the current revision of the Declaration of Helsinki.  The study will also be carried out in keeping with local legal and regulatory requirements (Austrian Drug Law).
11.2.	 Delegation of investigator responsibilities
The investigator should ensure that all persons assisting with the trial are adequately informed about the protocol, any amendments to the protocol, the study treatments, and their trial-related duties and functions.
The investigator should maintain a list of sub-investigators and other appropriately qualified persons to whom he or she has delegated significant trial-related duties.
11.3.	 Patient information and informed consent
Before being admitted to the clinical study, the patient must consent to participate after the nature, scope, and possible consequences of the clinical study have been explained in a form understandable to him or her.
An informed consent document that includes both information about the study and the consent form will be prepared and given to the patient. The document must be in a language understandable to the patient and must specify who informed the patient.  Where required by local law, the person who informs the patient must be a physician. 
After reading the informed consent document, the patient must give consent in writing. The patient's consent must be confirmed at the time of consent by the personally dated signature of the patient and the personally dated signature of the person conducting the informed consent discussions.
A copy of the signed consent document must be given to the patient. The original signed consent document will be retained by the investigator.
The investigator will not undertake any measures specifically required only for the clinical study until valid consent has been obtained.
It is recommended that the investigator inform the patient's primary physician about the patient's participation in the trial if the patient has a primary physician and if the patient agrees to the primary physician being informed.  
11.4.	 Confidentiality
Patient names will not be supplied to the sponsor.  Only the patient number and patient initials will be recorded in the case report form, and if the patient name appears on any other document (e.g., pathologist report), it must be obliterated before a copy of the document is supplied to the sponsor.  Study findings stored on a computer will be stored in accordance with local data protection laws.  The patients will be told that representatives of the sponsor, IEC/IRB, or regulatory authorities may inspect their medical records to verify the information collected, and that all personal information made available for inspection will be handled in strictest confidence and in accordance with local data protection laws.
The investigator will maintain a personal patient identification list (patient numbers with the corresponding patient names) to enable records to be identified.
11.5.	 Protocol amendments
Neither the investigator nor the sponsor will alter this study protocol without obtaining the written agreement of the other.  Once the study has started, amendments should be made only in exceptional cases. The changes then become part of the study protocol.
11.6.	 Approval of the study protocol and amendments
Before the start of the study, the study protocol, informed consent document, and any other appropriate documents will be submitted to the independent ethics committee(IEC)/institutional review board (IRB) with a cover letter or a form listing the documents submitted, their dates of issue, and the site for which approval is sought.  If applicable, the documents will also be submitted to the authorities, in accordance with local legal requirements.
Study medication can only be supplied to the investigator after documentation on all ethical and legal requirements for starting the study has been received by the sponsor.  This documentation must also include a list of the members of the IEC/IRB and their occupation and qualifications.  If the IEC/IRB will not disclose the names of the committee members, it should be asked to issue a statement confirming that the composition of the committee is in accordance with GCP.  Formal approval by the IEC/IRB should preferably mention the study title, study code, study site (or region or area of jurisdiction, as applicable), and any other documents reviewed.  It must mention the date on which the decision was made and must be officially signed by a committee member.
Before the first patient is enrolled in the study, all ethical and legal requirements must be met.
The IEC/IRB and, if applicable, the authorities must be informed of all subsequent protocol amendments, in accordance with local legal requirements.  Amendments must be evaluated to determine whether formal approval must be sought and whether the informed consent document should also be revised.
The investigator must keep a record of all communication with the IEC/IRB and, if applicable, between a coordinating investigator and the IEC/IRB.  This also applies to any communication between the investigator (or coordinating investigator, if applicable) and the authorities.
11.7.	 Ongoing information for independent ethics committee/ institutional review board
If required by legislation or the IEC/IRB, the investigator must submit to the IEC/IRB:
	Information on serious or unexpected adverse events as soon as possible
	Periodic reports on the progress of the study
11.8.	 Premature closure of the study
The sponsor or the investigator has the right to close this study at any time.  As far as possible, this should occur after mutual consultation.  The IEC/IRB must be informed, if required by legislation.
Should the study be closed prematurely, all study materials (completed, partially completed, and blank case report forms, study medication, etc.) must be returned to the sponsor, as if the study had been completed.
11.9.	 Record retention
-	The following records must be retained by the investigator for a minimum of 15 years after the completion or termination of the study: 
-	Signed informed consent documents for all patients
-	Patient identification code list, screening log (if applicable), and enrolment log
-	Record of all communications between the investigator and the EC 
-	Composition of the EC  (or other applicable statement as described in Section  Approval of the study protocol and amendments, page 49) 
-	List of sub-investigators and other appropriately qualified persons to whom the investigator has delegated significant trial-related duties, together with their roles in the study and their signatures
-	Copies of case report forms and of documentation of corrections for all patients
-	Record of any body fluids or tissue samples retained
-	All other source documents (patient records, hospital records, laboratory records, etc.)
-	All other documents as listed in Section 8 of the ICH consolidated guideline on GCP (Essential Documents for the Conduct of a Clinical Trial)
  
11.10.	 Liability and insurance
The investigator has subscribed to an insurance policy covering, in its terms and provisions, its legal liability for injuries caused to participating persons and arising out of this research performed strictly in accordance with the scientific protocol as well as with applicable law and professional standards. 


12.	  Study monitoring and auditing
Monitoring and auditing procedures developed by the sponsor will be followed, in order to comply with GCP guidelines.  On-site checking of the case report forms for completeness and clarity, cross-checking with source documents, and clarification of administrative matters will be performed.
12.1.   Study monitoring
The study will be monitored by the monitoring team defined by the Principal Investigator (see page 3).  Monitoring will be done by personal visits reviewing the case report forms and source documents.  By frequent communications (letter, telephone, and fax), the site monitor will ensure that the investigation is conducted according to protocol design and regulatory requirements..
12.2.	 Source data verification and on-site audits
Regulatory authorities may request access to all source documents, case report forms, and other study documentation for on-site audit or inspection.  Direct access to these documents must be guaranteed by the investigator, who must provide support at all times for these activities.


13.	    Documentation and use of study findings
A case report form will be provided by the principal investigator for each patient.
All protocol-required information collected during the study must be entered by the investigator, or designated representative, in the case report form.  Details of case report form completion and correction will be explained to the investigator.  If the investigator authorizes other persons to make entries in the case report form, the names, positions, signatures, and initials of these persons must be supplied to the sponsor.
The investigator, or designated representative, should complete the case report form pages as soon as possible after information is collected, preferably on the same day that a study patient is seen for an examination, treatment, or any other study procedure.  Any outstanding entries must be completed immediately after the final examination.  An explanation should be given for all missing data.
A source data location list will be prepared and updated during the study.  This list will be filed in both the trial master file and the investigator study file.
The completed case report form must be reviewed and signed by the investigator named in the study protocol or by a designated sub-investigator.
The study center will retain the originals of all case report forms.  The investigator will retain a copy of all completed case report form pages.
 
14.	   Study duration and dates

Start enrollment date:		 01.10.2006
Stop enrollment date:		 30.09.2009
Planned duration of study:	 40 months
Planned enrollment duration:	 36 months
First study report:		 3 months after last patient
Final study report:		12 months after last patient

15.	Authorship
All persons designated as authors should qualify for authorship. Each author should have participated sufficiently in the work to take public responsibility for the content.
Authorship credit should be based on substantial contributions to
1.	conception and design, or analysis and interpretation of data; and to
2.	drafting the article or revising it critically for important intellectual content; and
3.	on final approval of the version to be published.
Conditions 1, 2 and 3 must all be met.


16.	 Declaration OF investigator
I have read this study protocol and agree that it contains all the information required to conduct the study.  I agree to conduct the study as set out in this protocol.
I will not enroll the first patient in the study until I have received approval from the EC and until all legal requirements in my country have been fulfilled.
The study will be conducted in accordance with the moral, ethical, and scientific principles governing clinical research as set out in the Declaration of Helsinki and the ICH Harmonised Tripartite Guideline Topic E 6:  “Guideline for Good Clinical Practice” as well as in the applicable local guidelines.
I agree to obtain, in the manner described in this study protocol, written informed consent or witnessed verbal informed consent to participate for all patients enrolled in this study.
I am aware of the requirements for the correct reporting of serious adverse events, and I undertake to document and to report such events as requested.
I agree with the use of results of the study for the purposes of national and international registration, publication, and information for medical and pharmaceutical professionals.
I agree to keep all source documents and case report forms as specified in Section 11.9   Record retention.
I will provide a curriculum vitae before the study starts, which may be submitted to regulatory authorities.
Investigator
Date:		Signature:	
			Name (block letters):  Univ.-Prof. Dr. Wolfgang HILBE 	


17.	       Appendices
17.1.	       APPENDIX I: RECIST GUIDELINES FOR EVALUATION OF RESPONSE
17.1.1.	Response evaluation criteria in solid tumors (RECIST) Quick Reference
17.1.2.	Eligibility
·	Only patients with measurable disease at baseline should be included in protocols where objective tumor response is the primary endpoint. 
o	Measurable disease - the presence of at least one measurable lesion. If the measurable disease is restricted to a solitary lesion, its neoplastic nature should be confirmed by cytology/histology. 
o	Measurable lesions - lesions that can be accurately measured in at least one dimension with longest diameter  20 mm using conventional techniques or 10 mm with spiral CT scan.
o	Non-measurable lesions - all other lesions, including small lesions (longest diameter <20 mm with conventional techniques or <10 mm with spiral CT scan), i.e., bone lesions, leptomeningeal disease, ascites, pleural/pericardial effusion, inflammatory breast disease, lymphangitis cutis/pulmonis, cystic lesions, and also abdominal masses that are not confirmed and followed by imaging techniques; and.
·	All measurements should be taken and recorded in metric notation, using a ruler or calipers. All baseline evaluations should be performed as closely as possible to the beginning of treatment and never more than 4 weeks before the beginning of the treatment. 
·	The same method of assessment and the same technique should be used to characterize each identified and reported lesion at baseline and during follow-up. 
·	Clinical lesions will only be considered measurable when they are superficial (e.g., skin nodules and palpable lymph nodes). For the case of skin lesions, documentation by color photography, including a ruler to estimate the size of the lesion, is recommended. 

17.1.3.	Methods of Measurement 

·	CT and MRI are the best currently available and reproducible methods to measure target lesions selected for response assessment. Conventional CT and MRI should be performed with cuts of 10 mm or less in slice thickness contiguously. Spiral CT should be performed using a 5 mm contiguous reconstruction algorithm.  This applies to tumors of the chest, abdomen and pelvis. Head and neck tumors and those of extremities usually require specific protocols.

·	Lesions on chest X-ray are acceptable as measurable lesions when they are clearly defined and surrounded by aerated lung. However, CT is preferable. 

·	When the primary endpoint of the study is objective response evaluation, ultrasound (US) should not be used to measure tumor lesions. It is, however, a possible alternative to clinical measurements of superficial palpable lymph nodes, subcutaneous lesions and thyroid nodules. US might also be useful to confirm the complete disappearance of superficial lesions usually assessed by clinical examination.

·	The utilization of endoscopy and laparoscopy for objective tumor evaluation has not yet been fully and widely validated. Their uses in this specific context require sophisticated equipment and a high level of expertise that may only be available in some centers. Therefore, the utilization of such techniques for objective tumor response should be restricted to validation purposes in specialized centers. However, such techniques can be useful in confirming complete pathological response when biopsies are obtained.

·	Tumour markers alone cannot be used to assess response.  If markers are initially above the upper normal limit, they must normalize for a patient to be considered in complete clinical response when all lesions have disappeared.

·	Cytology and histology can be used to differentiate between PR and CR in rare cases (e.g., after treatment to differentiate between residual benign lesions and residual malignant lesions in tumor types such as germ cell tumors).


17.1.4.	  Baseline Documentation of “Target” and “Non-Target” Lesions
·	All measurable lesions up to a maximum of five lesions per organ and 10 lesions in total, representative of all involved organs should be identified as target lesions and should be  recorded and measured at baseline. 
·	Target lesions should be selected on the basis of their size (lesions with the longest diameter) and their suitability for accurate repeated measurements (either by imaging techniques or clinically). 
·	A sum of the longest diameter (LD) for all target lesions will be calculated and reported as the baseline sum LD. The baseline sum LD will be used as reference by which to characterize the objective tumor.
·	All other lesions (or sites of disease) should be identified as non-target lesions and should also be recorded at baseline. Measurements of these lesions are not required, but the presence or absence of each should be noted throughout follow-up. 

17.1.5.	  Response Criteria
17.1.5.1.	Evaluation of target lesions (modified RECIST criteria)
·	Complete Response
       (CR):  
	Disappearance of all target lesions	
·	Partial Response (PR):
	At least a 30% decrease in the sum of the LD of target lesions, taking        as reference the baseline sum LD	
·	Progressive Disease (PD):
	At least a 20% increase in the sum of the LD of target lesions, taking as reference the smallest sum LD recorded since the treatment started or the appearance of one or more new lesions	
·	Stable Disease
      (SD):	Neither sufficient shrinkage to qualify for PR nor sufficient increase to qualify for PD, taking as reference the smallest sum LD since the treatment started	
17.1.5.2.	 Evaluation of non-target lesions
·	   Complete Response(CR):	Disappearance of all non-target lesions and normalization of tumor marker level	
·	Incomplete Response/          Stable Disease (SD): 	Persistence of one or more non-target lesion(s) or/and maintenance of tumor marker level above the normal limits	
·	Progressive Disease (PD):	Appearance of one or more new lesions and/or unequivocal progression of existing non-target lesions (1) 	
(1)	Although a clear progression of “non target” lesions only is exceptional, in such circumstances, the opinion of the treating physician should prevail and the progression status should be confirmed later on by the review panel (or study chair). 

All tumour lesion defined by PET, bronchoscopy and medianstinoscopy/selective puncture of mediastinal lymph-nodes are non-target lesion.

17.1.6.	Evaluation of best overall response
The best overall response is the best response recorded from the start of the treatment until disease progression/recurrence (taking as reference for PD the smallest measurements recorded since the treatment started). In general, the patient's best response assignment will depend on the achievement of both measurement and confirmation criteria 

Target lesions	Non-Target lesions	New Lesions	Overall response	
CR	CR	No	CR	
CR	Incomplete response/SD	No	PR	
PR	Non-PD	No	PR	
SD	Non-PD	No	SD	
PD	Any	Yes or No	PD	
Any	PD	Yes or No	PD	
Any	Any	Yes	PD	

·	Patients with a global deterioration of health status requiring discontinuation of treatment without objective evidence of disease progression at that time should be classified as having “symptomatic deterioration”. Every effort should be made to document the objective progression even after discontinuation of treatment. 
·	In some circumstances it may be difficult to distinguish residual disease from normal tissue. When the evaluation of complete response depends on this determination, it is recommended that the residual lesion be investigated (fine needle aspirate/biopsy) to confirm the complete response status.

17.1.7.	Confirmation
Due to the study protocol, confirmation of response is not feasible.

17.1.7.1.	Duration of overall response
The duration of overall response is measured from the time measurement criteria are met for CR or PR (whichever status is recorded first) until the first date that recurrence or PD is objectively documented, taking as reference for PD the smallest measurements recorded since the treatment started.

17.1.7.2.	Duration of stable disease
·	SD is measured from the start of the treatment until the criteria for disease progression are met, taking as reference the smallest measurements recorded since the treatment started. 
·	The clinical relevance of the duration of SD varies for different tumor types and grades. Therefore, it is highly recommended that the protocol specify the minimal time interval required between two measurements for determination of SD. This time interval should take into account the expected clinical benefit that such a status may bring to the population under study. 

17.1.8.	Response review
For trials where the response rate is the primary endpoint it is strongly recommended that all responses be reviewed by an expert(s) independent of the study at the study's completion.  Simultaneous review of the patients' files and radiological images is the best approach. 

17.1.9.	Reporting of results
·	All patients included in the study must be assessed for response to treatment, even if there are major protocol treatment deviations or if they are ineligible.  Each patient will be assigned one of the following categories: 1) complete response, 2) partial response, 3) stable disease, 4) progressive disease, 5) early death from malignant disease, 6) early death from toxicity, 7) early death because of other cause, or 9) unknown (not assessable, insufficient data).
·	All of the patients who met the eligibility criteria should be included in the main analysis of the response rate.  Patients in response categories 4-9 should be considered as failing to respond to treatment (disease progression).  Thus, an incorrect treatment schedule or drug administration does not result in exclusion from the analysis of the response rate.  Precise definitions for categories 4-9 will be protocol specific.
·	All conclusions should be based on all eligible patients.
·	Sub-analyses may then be performed on the basis of a subset of patients, excluding those for whom major protocol deviations have been identified (e.g., early death due to other reasons, early discontinuation of treatment, major protocol violations, etc.).  However, these sub-analyses may not serve as the basis for drawing conclusions concerning treatment efficacy, and the reasons for excluding patients from the analysis should be clearly reported.  
·	The 95% confidence intervals should be provided.
 
17.2.	APPENDIX II: GUIDELINES FOR EVALUATION OF RESECTABILITY ACCORDING TO “TAKO RECOMMENDATONS 2006” (homepage www.tako.or.at)


17.3.	APPENDIX III:  NCI CTC Toxicity scale Version 2.0 

              COMMON TOXICITY CRITERIA (NCI CTC)

Grade	
Toxicity	0	1	2	3	4	
ALLERGY/IMMUNOLOGY	
Allergic reaction/ hypersensitivity 
(including drug fever)	none	transient rash, drug fever < 38°C (<100.4°F)	urticaria, drug fever  38°C (100.4°F), and/or asymptomatic bronchospasm	symptomatic bronchospasm, requiring parenteral medication(s), with or without urticaria; allergy-related edema/angioedema	anaphylaxis	
Note: Isolated urticaria, in the absence of other manifestations of an allergic or hypersensitivity reaction, is graded in the DERMATOLOGY/SKIN category.	
Allergic rhinitis 
(including sneezing, nasal stuffiness, postnasal drip)	none	mild, not requiring treatment	moderate, requiring treatment	-	-	
Autoimmune reaction	none	serologic or other evidence of autoimmune reaction but patient is asymptomatic (e.g., vitiligo), all organ function is normal and no treatment is required	evidence of autoimmune reaction involving a non-essential organ or function (e.g., hypothyroidism), requiring treatment other than immunosuppressive drugs	reversible autoimmune reaction involving function of a major organ or other toxicity (e.g., transient colitis or anemia), requiring short-term immunosuppressive treatment	autoimmune reaction causing major grade 4 organ dysfunction; progressive and irreversible reaction; long-term administration of high-dose immuno-suppressive therapy required	
Also consider Hypothyroidism, Colitis, Hemoglobin, Hemolysis.	
Serum sickness	none	-	-	present	-	
Urticaria is graded in the DERMATOLOGY/SKIN category if it occurs as an isolated symptom. If it occurs with other manifestations of allergic or hypersensitivity reaction, grade as Allergic reaction/hypersensitivity above.	
Vasculitis	none	mild, not requiring treatment	symptomatic, requiring medication	requiring steroids	ischemic changes or requiring amputation	
Allergy/Immunology-Other
(Specify, __________)	none	mild	moderate	severe	life-threatening or disabling	
AUDITORY/HEARING	
Conductive hearing loss is graded as Middle ear/hearing in the AUDITORY/HEARING category.	
Earache is graded in the PAIN category.	

External auditory canal	normal	external otitis with erythema or dry desquamation	external otitis with moist desquamation	external otitis with discharge, mastoiditis	necrosis of the canal soft tissue or bone	
Note: Changes associated with radiation to external ear (pinnae) are graded under Radiation dermatitis in the DERMATOLOGY/SKIN category.	
Inner ear/hearing	normal	hearing loss on audiometry only	tinnitus or hearing loss, not requiring hearing aid or treatment	tinnitus or hearing loss, correctable with hearing aid or treatment	severe unilateral or bilateral hearing loss (deafness), not correctable	
Middle ear/hearing	normal	serous otitis without subjective decrease in hearing	serous otitis or infection requiring medical intervention; subjective decrease in hearing; rupture of tympanic membrane with discharge	otitis with discharge, mastoiditis or conductive hearing loss	necrosis of the canal soft tissue or bone	
Auditory/Hearing-Other
(Specify, __________)	normal	mild	moderate	severe	life-threatening or disabling	
BLOOD/BONE MARROW	
Bone marrow cellularity	normal for age	mildly hypocellular or 25% reduction from normal cellularity for age	moderately hypocellular or >25 -  50% reduction from normal cellularity for age or >2 but <4 weeks to recovery of normal bone marrow cellularity	severely hypocellular or >50 -  75% reduction in cellularity for age or 4 - 6 weeks to recovery of normal bone marrow cellularity	aplasia or >6 weeks to recovery of normal bone marrow cellularity	
Normal ranges:						
children ( 18 years)	90% cellularity average					
younger adults (19-59)	60-70% cellularity average					
older adults ( 60 years)	50% cellularity average					
Note: Grade Bone marrow cellularity only for changes related to treatment not disease.	
CD4 count	WNL	< LLN - 500/mm3	200 - < 500/mm3	50 - < 200/mm3	< 50/mm3	
Haptoglobin	normal	decreased	-	absent	-	
Hemoglobin (Hgb)	WNL	< LLN - 10.0 g/dl
< LLN - 100 g/L
< LLN - 6.2 mmol/L	8.0 - < 10.0 g/dl
80 - < 100 g/L
4.9 - < 6.2 mmol/L	6.5 - < 8.0 g/dl
65 - 80 g/L
4.0 - < 4.9 mmol/L	< 6.5 g/dl
< 65 g/L
< 4.0 mmol/L	
Note: The following criteria may be used for leukemia studies or bone marrow infiltrative/myelophthisic process if the protocol so specifies.	
For leukemia studies or bone marrow infiltrative/ myelophthisic processes	WNL	10 - <25% decrease from pretreatment	25 - <50% decrease from pretreatment	50 - <75% decrease from pretreatment	75% decrease from pretreatment	
Hemolysis (e.g., immune hemolytic anemia, drug-related hemolysis, other)	none	only laboratory evidence of hemolysis [e.g., direct antiglobulin test (DAT, Coombs') schistocytes]	evidence of red cell destruction and  2gm decrease in hemoglobin, no transfusion	requiring transfusion and/or medical intervention (e.g., steroids)	catastrophic consequences of hemolysis (e.g., renal failure, hypotension, bronchospasm, emergency splenectomy)	
Also consider Haptoglobin, Hgb.	
Leukocytes (total WBC)	WNL	< LLN - 3.0 x 10^9^ /L
< LLN - 3000/mm3	2.0 - < 3.0 x 10^9^ /L
2000 - < 3000/mm3	1.0 - < 2.0 x 10^9^ /L
1000 - < 2000/mm3	< 1.0 x 10^9^ /L
< 1000/mm3	
For BMT studies:	WNL	2.0 - <3.0 X 109/L
2000 - <3000/mm3	1.0 - <2.0 x 10^9^ /L 1000 - <2000/mm3	0.5 - <1.0 x 10^9^ /L 500 - <1000/mm3	<0.5 x 10^9^ /L
<500/mm3	
Note: The following criteria using age, race and sex normal values may be used for pediatric studies if the protocol so specifies.	
		75 - <100% LLN	50 - <75% LLN	25 - 50% LLN	<25% LLN	
Lymphopenia	WNL	<LLN - 1.0 x 10^9^ /L
<LLN - 1000/mm3	0.5 - <1.0 x 10^9^ /L
500 - <1000/mm3	<0.5 x 10^9^ /L
<500/mm3	-	
Note: The following criteria using age, race, and sex normal values may be used for pediatric studies if the protocol so specifies.	
		75-<100%LLN	50-<75%LLN	25-<50%LLN	<25%LLN	
Neutrophils/granulocytes
(ANC/AGC)	WNL	1.5 - <2.0 x 10^9^ /L
1500 - <2000/mm3	1.0 - <1.5 x 10^9^ /L
1000 - <1500/mm3	0.5 - <1.0 x 10^9^ /L
500 - <1000/mm3	< 0.5 x 10^9^ /L
< 500/mm3	
For BMT:	WNL	1.0 - <1.5 x 10^9^ /L
1000 - <1500/mm3	0.5 - <1.0 x 10^9^ /L
500 - <1000/mm3	0.1 - <0.5 x 10^9^ /L
100 - <500/mm3	<0.1 x 10^9^ /L
<100/mm3	
Note: The following criteria may be used for leukemia studies or bone marrow infiltrative/myelophthisic process if the protocol so specifies.	
For leukemia studies or bone marrow infiltrative/
myelophthisic process	WNL	10 - <25% decrease from baseline	25 - <50% decrease from baseline	50 - <75% decrease from baseline	75% decrease from baseline	
Platelets	WNL	< LLN - <75.0 x 109 /L
< LLN - 75000/mm3	50.0 - < 75.0 x 109 /L
50000 - < 75000/mm3	10.0 - < 50.0 x 109 /L
10000 - < 50000/mm3	< 10.0 x 109 /L
< 10000/mm3	
For BMT:	WNL	50.0 - <75.0 x 109 /L
50000 - <75000/mm3 	20.0 - <50.0 x 109 /L
20000 - <50000/mm3	10.0 - <20.0 x 109 /L
10000 - <20000/mm3	<10.0 x 109 /L
<10000/mm3	
Note: The following criteria may be used for leukemia studies or bone marrow infiltrative/myelophthisic process if the protocol so specifies.	
For leukemia studies or bone marrow infiltrative/
myelophthisic process	WNL	10 - <25% decrease from baseline	25 - <50% decrease from baseline	50 - <75% decrease from baseline	75% decrease from baseline	
Transfusion: Platelets	none	-	-	yes	platelet transfusions and other measures required to improve platelet increment; platelet transfusion refractoriness associated with life-threatening bleeding. (e.g., HLA or cross matched platelet transfusions)	
For BMT:	none	1 platelet transfusion in 24 hours	2 platelet transfusions in 24 hours	3 platelet transfusions in 24 hours	platelet transfusions and other measures required to improve platelet increment; platelet transfusion refractoriness associated with life-threatening bleeding. (e.g., HLA or cross matched platelet transfusions)	
Also consider Platelets.	
Transfusion: pRBCs	none	-	-	Yes	-	
For BMT:	none	2 u pRBC (15cc/kg) in 24 hours elective or planned

	3 u pRBC (>15 30cc/kg) in 24 hours elective or planned	4 u pRBC (>30cc/kg) in 24 hours	hemorrhage or hemolysis associated with life-threatening anemia; medical intervention required to improve hemoglobin	
Also consider Hemoglobin.	
Blood/Bone Marrow-Other
(Specify, __________)	none	mild	moderate	severe	life-threatening or disabling	
CARDIOVASCULAR (ARRHYTHMIA)	
Conduction abnormality/ Atrioventricular heart block	none	asymptomatic, not requiring treatment (e.g., Mobitz type I second-degree AV block, Wenckebach)	symptomatic, but not requiring treatment	symptomatic and requiring treatment (e.g., Mobitz type II second-degree AV block, third-degree AV block)	life-threatening (e.g., arrhythmia associated with CHF, hypotension, syncope, shock)	
Nodal/junctional arrhythmia/dysrhythmia	none	asymptomatic, not requiring treatment	symptomatic, but not requiring treatment	symptomatic and requiring treatment	life-threatening (e.g., arrhythmia associated with CHF, hypotension, syncope, shock)	
Palpitations	none	present	-	-	-	
Note: Grade palpitations only in the absence of a documented arrhythmia.	
Prolonged QTc interval (QTc > 0.48 seconds)	none	asymptomatic, not requiring treatment	symptomatic, but not requiring treatment	symptomatic and requiring treatment	life-threatening (e.g., arrhythmia associated with CHF, hypotension, syncope, shock)	
Sinus bradycardia	none	asymptomatic, not requiring treatment	symptomatic, but not requiring treatment	symptomatic and requiring treatment	life-threatening (e.g., arrhythmia associated with CHF, hypotension, syncope, shock)	
Sinus tachycardia	none	asymptomatic, not requiring treatment	symptomatic, but not requiring treatment	symptomatic and requiring treatment of underlying cause	-	
Supraventricular arrhythmias (SVT/atrial fibrillation/ flutter)	none	asymptomatic, not requiring treatment	symptomatic, but not requiring treatment	symptomatic and requiring treatment	life-threatening (e.g., arrhythmia associated with CHF, hypotension, syncope, shock)	
Syncope (fainting) is graded in the NEUROLOGY category.	
Vasovagal episode	none	-	present without loss of consciousness	present with loss of consciousness	-	
Ventricular arrhythmia (PVCs/bigeminy/trigeminy/
ventricular tachycardia)	none	asymptomatic, not requiring treatment	symptomatic, but not requiring treatment	symptomatic and requiring treatment	life-threatening (e.g., arrhythmia associated with CHF, hypotension, syncope, shock)	
Cardiovascular/
Arrhythmia-Other 
(Specify, ___________)	none	asymptomatic, not requiring treatment	symptomatic, but not requiring treatment	symptomatic, and requiring treatment of underlying cause	life-threatening (e.g., arrhythmia associated with CHF, hypotension, syncope, shock)	
CARDIOVASCULAR (GENERAL)	
Acute vascular leak syndrome	absent	-	symptomatic, but not requiring fluid support	respiratory compromise or requiring fluids	life-threatening; requiring pressor support and/or ventilatory support	
Cardiac- ischemia/infarction	none	non-specific T-wave flattening or changes	asymptomatic, ST- and T- wave changes suggesting ischemia	angina without evidence of infarction	acute myocardial infarction	
Cardiac left ventricular function	normal	asymptomatic decline of resting ejection fraction of  10% but < 20% of baseline value; shortening fraction  24% but < 30%	asymptomatic but resting ejection fraction below LLN for laboratory or decline of resting ejection fraction  20% of baseline value; < 24% shortening fraction	CHF responsive to treatment	severe or refractory CHF or requiring intubation	
CNS cerebrovascular ischemia is graded in the NEUROLOGY category.	
Cardiac troponin I (cTnI)	normal	-	-	levels consistent with unstable angina as defined by the manufacturer	levels consistent with myocardial infarction as defined by the manufacturer	
Cardiac troponin T (cTnT)	normal	 0.03 - < 0.05 ng/ml	 0.05 - < 0.1 ng/ml	 0.1 - < 0.2 ng/ml	 0.2 ng/ml	
Edema	none	asymptomatic, not requiring therapy	symptomatic, requiring therapy	symptomatic edema limiting function and unresponsive to therapy or requiring drug discontinuation	anasarca (severe generalized edema)	
Hypertension	none	asymptomatic, transient increase by >20 mmHg (diastolic) or to > 150/100* if previously WNL; not requiring treatment	recurrent or persistent or symptomatic increase by > 20 mmHg (diastolic) or to > 150/100* if previously WNL; not requiring treatment	requiring therapy or more intensive therapy than previously	hypertensive crisis	
*Note: For pediatric patients, use age and sex appropriate normal values > 95th percentile ULN.	
Hypotension	none	changes, but not requiring therapy (including transient orthostatic hypotension)	requiring brief fluid replacement or other therapy but not hospitalization; no physiologic consequences	requiring therapy and sustained medical attention, but resolves without persisting physiologic consequences	shock (associated with acidemia and impairing vital organ function due to tissue hypoperfusion)	
Also consider Syncope (fainting).	
Note:	Angina or MI is graded as Cardiac- ischemia/infarction in the CARDIOVASCULAR (GENERAL) category.	
For pediatric patients, systolic BP 65 mmHg or less in infants up to 1 year old and 70 mmHg or less in children older than 1 year of age, use two successive or three measurements in 24 hours.	
Myocarditis	none	-	-	CHF responsive to treatment	severe or refractory CHF	
Operative injury of vein/artery	none	primary suture repair for injury, but not requiring transfusion	primary suture repair for injury, requiring transfusion	vascular occlusion requiring surgery or bypass for injury	myocardial infarction; resection of organ (e.g., bowel, limb)	
Pericardial effusion/ pericarditis	none	asymptomatic effusion, not requiring treatment	pericarditis (rub, ECG changes, and/or chest pain)	physiologic consequences resulting from symptoms	tamponade (drainage or pericardial window required)	
Peripheral arterial ischemia	none	-	brief episode of ischemia managed non-surgically and without permanent deficit	requiring surgical intervention	life-threatening or with permanent functional deficit (e.g., amputation)	
Phlebitis (superficial)	none	-	present	-	-	
Note:	Injection site reaction is graded in the DERMATOLOGY/SKIN category.	
	Thrombosis/embolism is graded in the CARDIOVASCULAR (GENERAL) category.	
Syncope (fainting) is graded in the NEUROLOGY category.	
Thrombosis/embolism	none	-	deep vein thrombosis, not requiring anticoagulant	deep vein thrombosis, requiring anticoagulant therapy	embolic event including pulmonary embolism	
Vein/artery operative injury is graded as Operative injury of vein/artery in the CARDIOVASCULAR (GENERAL) category.	
Visceral arterial ischemia (non-myocardial)	none	-	brief episode of ischemia managed non-surgically and without permanent deficit	requiring surgical intervention	life-threatening or with permanent functional deficit (e.g., resection of ileum)	
Cardiovascular/
General-Other 
(Specify, ______________)	none	mild	moderate	severe	life-threatening or disabling	
COAGULATION	
Note: See the HEMORRHAGE category for grading the severity of bleeding events.	
DIC 
(disseminated intravascular coagulation)	absent	-	-	laboratory findings present with no bleeding	laboratory findings and bleeding	
Also grade Platelets.	
Note: Must have increased fibrin split products or D-dimer in order to grade as DIC.	
Fibrinogen	WNL	0.75 - <1.0 x LLN	0.5 - <0.75 x LLN	0.25 - <0.5 x LLN	<0.25 x LLN	
Note: The following criteria may be used for leukemia studies or bone marrow infiltrative/myelophthisic process if the protocol so specifies.	
For leukemia studies:	WNL	<20% decrease from pretreatment value or LLN	20 - <40% decrease from pretreatment value or LLN	40 - <70% decrease from pretreatment value or LLN	<50 mg%	
Partial thromboplastin time (PTT)	WNL	> ULN -  1.5 x ULN	> 1.5 -  2 x ULN	>2 x ULN	-	
Phelbitis is graded in the CARDIOVASCULAR (GENERAL) category.	
Prothrombin time (PT)	WNL	> ULN -  1.5 x ULN	> 1.5 -  2 x ULN	>2 x ULN	-	
Thrombosis/embolism is graded in the CARDIOVASCULAR (GENERAL) category.	
Thrombotic microangiopathy (e.g., thrombotic thrombocytopenic purpura/TTP or hemolytic uremic syndrome/HUS)	absent	-	-	laboratory findings present without clinical consequences	laboratory findings and clinical consequences, (e.g., CNS hemorrhage/ bleeding or thrombosis/ embolism or renal failure) requiring therapeutic intervention	
For BMT:	-	evidence of RBC destruction (schistocytosis) without clinical consequences	evidence of RBC destruction with elevated creatinine (3 x ULN)	evidence of RBC destruction with creatinine (>3 x ULN) not requiring dialysis	evidence of RBC destruction with renal failure requiring dialysis and/or encephalopathy	
Also consider Hemoglobin (Hgb), Platelets, Creatinine.	
Note: Must have microangiopathic changes on blood smear (e.g., schistocytes, helmet cells, red cell fragments).	
Coagulation-Other
(Specify, __________)	none	mild	moderate	severe	life-threatening or disabling	
CONSTITUTIONAL SYMPTOMS	
Fatigue
(lethargy, malaise, asthenia)	none	increased fatigue over baseline, but not altering normal activities	moderate (e.g., decrease in performance status by 1 ECOG level or 20% Karnofsky or Lansky) or causing difficulty performing some activities	severe (e.g., decrease in performance status by 2 ECOG levels or 40% Karnofsky or Lansky) or loss of ability to perform some activities	bedridden or disabling	
Note: See Appendix III for performance status scales.	
Fever (in the absence of neutropenia, where neutropenia is defined as AGC < 1.0 x 10^9^/L)	none	38.0 - 39.0°C (100.4 - 102.2°F)	39.1 - 40.0°C (102.3 - 104.0°F )	> 40.0°C (>104.0°F ) for < 24hrs	> 40.0°C (>104.0°F ) for > 24hrs	
Also consider Allergic reaction/hypersensitivity.	
Note: The temperature measurements listed above are oral or tympanic.	
Hot flashes/flushes are graded in the ENDOCRINE category.	
Rigors, chills	none	mild, requiring symptomatic treatment (e.g., blanket) or non-narcotic medication	severe and/or prolonged, requiring narcotic medication	not responsive to narcotic medication	-	
Sweating 
(diaphoresis)	normal	mild and occasional	frequent or drenching	-	-	
Weight gain	< 5%	5 - <10%	10 - <20%	 20%	-	
Also consider Ascites, Edema, Pleural effusion.	
Weight gain - veno-occlusive disease (VOD)						
Note: The following criteria is to be used ONLY for weight gain associated with Veno-Occlusive Disease.	
	<2%	2 - <5%	5 - <10% 	10% or as ascities	10% or fluid retention resulting in pulmonary failure	
Weight loss	< 5%	5 - <10%	10 - <20%	20%	-	
Also consider Vomiting, Dehydration, Diarrhea.	
Constitutional Symptoms-Other
(Specify, __________)	none	mild	moderate	severe	life-threatening or disabling	
DERMATOLOGY/SKIN	
Alopecia	normal	mild hair loss	pronounced hair loss	-	-	
Bruising 
(in absence of grade 3 or 4 thrombocytopenia)	none	localized or in dependent area	generalized	-	-	
Note:	Bruising resulting from grade 3 or 4 thrombocytopenia is graded as Petechiae/purpura and Hemorrhage/bleeding with grade 3 or 4 thrombocytopenia in the HEMORRHAGE category, not in the DERMATOLOGY/SKIN category.	
Dermatitis, focal (associated with high-dose chemotherapy and bone marrow transplant)	none	faint erythema or dry desquamation	moderate to brisk erythema or a patchy moist desquamation, mostly confined to skin folds and creases; moderate edema	confluent moist desquamation, 1.5 cm diameter, not confined to skin folds; pitting edema	skin necrosis or ulceration of full thickness dermis; may include spontaneous bleeding not induced by minor trauma or abrasion	
Dry skin	normal	controlled with emollients	not controlled with emollients	-	-	
Erythema multiforme (e.g., Stevens-Johnson syndrome, toxic epidermal necrolysis)	absent	-	scattered, but not generalized eruption	severe or requiring IV fluids (e.g., generalized rash or painful stomatitis)	life-threatening (e.g., exfoliative or ulcerating dermatitis or requiring enteral or parenteral nutritional support)	
Flushing	absent	present	-	-	-	
Hand-foot skin reaction	none	skin changes or dermatitis without pain (e.g., erythema, peeling)	skin changes with pain, not interfering with function	skin changes with pain, interfering with function	-	
Injection site reaction	none	pain or itching or erythema	pain or swelling, with inflammation or phlebitis	ulceration or necrosis that is severe or prolonged, or requiring surgery	-	
Nail changes	normal	discoloration or ridging (koilonychia) or pitting	partial or complete loss of nail(s) or pain in nailbeds	-	-	
Petechiae is graded in the HEMORRHAGE category.	
Photosensitivity	none	painless erythema	painful erythema	erythema with desquamation	-	
Pigmentation changes (e.g., vitiligo)	none	localized pigmentation changes	generalized pigmentation changes	-	-	
Pruritus	none	mild or localized, relieved spontaneously or by local measures	intense or widespread, relieved spontaneously or by systemic measures	intense or widespread and poorly controlled despite treatment	-	
Purpura is graded in the HEMORRHAGE category.	
Radiation dermatitis	none	faint erythema or dry desquamation	moderate to brisk erythema or a patchy moist desquamation, mostly confined to skin folds and creases; moderate edema	confluent moist desquamation, 1.5 cm diameter, not confined to skin folds; pitting edema	skin necrosis or ulceration of full thickness dermis; may include bleeding not induced by minor trauma or abrasion	
Note: Pain associated with radiation dermatitis is graded separately in the PAIN category as Pain due to radiation.	
Radiation recall reaction (reaction following chemotherapy in the absence of additional radiation therapy that occurs in a previous radiation port)	none	faint erythema or dry desquamation	moderate to brisk erythema or a patchy moist desquamation, mostly confined to skin folds and creases; moderate edema	confluent moist desquamation, 1.5 cm diameter, not confined to skin folds; pitting edema	skin necrosis or ulceration of full thickness dermis; may include bleeding not induced by minor trauma or abrasion	
Rash/desquamation	none	macular or papular eruption or erythema without associated symptoms	macular or papular eruption or erythema with pruritus or other associated symptoms covering <50% of body surface or localized desquamation or other lesions covering <50% of body surface area	symptomatic generalized erythroderma or macular, papular or vesicular eruption or desquamation covering 50% of body surface area	generalized exfoliative dermatitis or ulcerative dermatitis	
For BMT:	none	macular or papular eruption or erythema covering <25% of body surface area without associated symptoms	macular or papular eruption or erythema with pruritis or other associated symptoms covering 25 - <50% of body surface or localized desquamation or other lesions covering 25 - <50% of body surface area	symptomatic generalized erythroderma or symptomatic macular, papular or vesicular eruption, with bullous formation, or desquamation covering 50% of body surface area 	generalized exfoliative dermatitis or ulcerative dermatitis or bullous formation	
Also consider Allergic reaction/hypersensitivity.	
Note: Erythema multiforme (Stevens-Johnson syndrome) is graded separately as Erythema multiforme.	
Urticaria 
(hives, welts, wheals)	none	requiring no medication	requiring PO or topical treatment or IV medication or steroids for <24 hours	requiring IV medication or steroids for 24 hours	-	
Wound- infectious	none	cellulitis	superficial infection	infection requiring IV antibiotics	necrotizing fascitis	
Wound- non-infectious	none	incisional separation	incisional hernia	fascial disruption without evisceration	fascial disruption with evisceration	
Dermatology/Skin-Other
(Specify, ________)	none	mild	moderate	severe	life-threatening or disabling	
ENDOCRINE	
Cushingoid appearance (e.g., moon face with or without buffalo hump, centripetal obesity, cutaneous striae)	absent	-	present	-	-	
Also consider Hyperglycemia, Hypokalemia.	
Feminization of male	absent	-	-	present	-	
Gynecomastia	none	mild	pronounced or painful	pronounced or painful and requiring surgery	-	
Hot flashes/flushes	none	mild or no more than 1 per day	moderate and greater than 1 per day	-	-	
Hypothyroidism	absent	asymptomatic,TSH elevated, no therapy given	symptomatic or thyroid replacement treatment given	patient hospitalized for manifestations of hypothyroidism	myxedema coma	
Masculinization of female	absent	-	-	present	-	
SIADH (syndrome of inappropriate antidiuretic hormone)	absent	-	-	present	-	
Endocrine-Other 
(Specify, __________)	none	mild	moderate	severe	life-threatening or disabling	
GASTROINTESTINAL	
Amylase is graded in the METABOLIC/LABORATORY category.	
Anorexia	none	loss of appetite	oral intake significantly decreased	requiring IV fluids	requiring feeding tube or parenteral nutrition	
Ascites (non-malignant)	none	asymptomatic	symptomatic, requiring diuretics	symptomatic, requiring therapeutic paracentesis	life-threatening physiologic consequences	
Colitis	none	-	abdominal pain with mucus and/or blood in stool	abdominal pain, fever, change in bowel habits with ileus or peritoneal signs, and radiographic or biopsy documentation	perforation or requiring surgery or toxic megacolon	
Also consider Hemorrhage/bleeding with grade 3 or 4 thrombocytopenia, Hemorrhage/bleeding without grade 3 or 4 thrombocytopenia, Melena/GI bleeding, Rectal bleeding/hematochezia, Hypotension.	
Constipation	none	requiring stool softener or dietary modification	requiring laxatives	obstipation requiring manual evacuation or enema	obstruction or toxic megacolon	
Dehydration	none	dry mucous membranes and/or diminished skin turgor	requiring IV fluid replacement (brief)	requiring IV fluid replacement (sustained)	physiologic consequences requiring intensive care; hemodynamic collapse	
Also consider Hypotension, Diarrhea, Vomiting, Stomatitis/pharyngitis (oral/pharyngeal mucositis).	
Diarrhea
Patients without colostomy:	none	increase of < 4 stools/day over pre-treatment	increase of 4-6 stools/day, or nocturnal stools	increase of 7 stools/day or incontinence; or need for parenteral support for dehydration	physiologic consequences requiring intensive care; or hemodynamic collapse	
Patients with a colostomy:	none	mild increase in loose, watery colostomy output compared with pretreatment	moderate increase in loose, watery colostomy output compared with pretreatment, but not interfering with normal activity	severe increase in loose, watery colostomy output compared with pretreatment, interfering with normal activity	physiologic consequences, requiring intensive care; or hemodynamic collapse	
For BMT	none	>500 - 1000ml of diarrhea/day	>1000 - 1500ml of diarrhea/day	>1500ml of diarrhea/day	severe abdominal pain with or without ileus	
For Pediatric BMT:		>5 - 10 ml/kg of diarrhea/day	>10 - 15 ml/kg of diarrhea/day	>15 ml/kg of diarrhea/day	-	
Also consider Hemorrhage/bleeding with grade 3 or 4 thrombocytopenia, Hemorrhage/bleeding without grade 3 or 4 thrombocytopenia, Pain, Dehydration, Hypotension.	
Duodenal ulcer (requires radiographic or endoscopic documentation)	none	-	requiring medical management or non-surgical treatment	uncontrolled by outpatient medical management; requiring hospitalization	perforation or bleeding, requiring emergency surgery	
Dyspepsia/heartburn	none	mild	moderate	severe	-	
Dysphagia, esophagitis, odynophagia (painful swallowing)	none	mild dysphagia, but can eat regular diet	dysphagia, requiring predominantly pureed, soft, or liquid diet	dysphagia, requiring IV hydration	complete obstruction (cannot swallow saliva) requiring enteral or parenteral nutritional support, or perforation	
Note: If toxicity is radiation-related, grade either under Dysphagia- esophageal related to radiation or Dysphagia- pharyngeal related to radiation.	
Dysphagia- esophageal related to radiation	none	mild dysphagia, but can eat regular diet	dysphagia, requiring predominantly liquid, pureed or soft diet	dysphagia requiring feeding tube, IV hydration or hyperalimentation	complete obstruction (cannot swallow saliva); ulceration with bleeding not induced by minor trauma or abrasion or perforation	
Also consider Pain due to radiation, Mucositis due to radiation.	
Note: Fistula is graded separately as Fistula- esophageal.	
Dysphagia - pharyngeal related to radiation	none	mild dysphagia, but can eat regular diet	dysphagia, requiring predominantly pureed, soft, or liquid diet	dysphagia, requiring feeding tube, IV hydration or hyperalimentation	complete obstruction (cannot swallow saliva); ulceration with bleeding not induced by minor trauma or abrasion or perforation	
Also consider Pain due to radiation, Mucositis due to radiation.	
Note: Fistula is graded separately as Fistula- pharyngeal.	
Fistula- esophageal	none	-	-	present	requiring surgery	
Fistula- intestinal	none	-	-	present	requiring surgery	
Fistula- pharyngeal	none	-	-	present	requiring surgery	
Fistula- rectal/anal	none	-	-	present	requiring surgery	
Flatulence	none	mild	moderate	-	-	
Gastric ulcer 
(requires radiographic or endoscopic documentation)	none	-	requiring medical management or non-surgical treatment	bleeding without perforation, uncontrolled by outpatient medical management; requiring hospitalization or surgery	perforation or bleeding, requiring emergency surgery	
Also consider Hemorrhage/bleeding with grade 3 or 4 thrombocytopenia, Hemorrhage/bleeding without grade 3 or 4 thrombocytopenia.	
Gastritis	none	-	requiring medical management or non-surgical treatment	uncontrolled by out-patient medical management; requiring hospitalization or surgery	life-threatening bleeding, requiring emergency surgery	
Also consider Hemorrhage/bleeding with grade 3 or 4 thrombocytopenia, Hemorrhage/bleeding without grade 3 or 4 thrombocytopenia.	
Hematemesis is graded in the HEMORRHAGE category.	
Hematochezia is graded in the HEMORRHAGE category as Rectal bleeding/hematochezia.	
Ileus (or neuroconstipation)	none	-	intermittent, not requiring intervention	requiring non-surgical intervention	requiring surgery	
Mouth dryness	normal	mild	moderate	-	-	
Mucositis						
Note:	Mucositis not due to radiation is graded in the GASTROINTESTINAL category for specific sites: Colitis, Esophagitis, Gastritis, Stomatitis/pharyngitis (oral/pharyngeal mucositis), and Typhlitis; or the RENAL/GENITOURINARY category for Vaginititis.	
Radiation-related mucositis is graded as Mucositis due to radiation.	
Mucositis due to radiation	none	erythema of the mucosa	patchy pseudomembranous reaction (patches generally  1.5 cm in diameter and non-contiguous)	confluent pseudomembranous reaction (contiguous patches generally > 1.5 cm in diameter)	necrosis or deep ulceration; may include bleeding not induced by minor trauma or abrasion	
Also consider Pain due to radiation.	
Note:	Grade radiation mucositis of the larynx here.	
Dysphagia related to radiation is also graded as either Dysphagia- esophageal related to radiation or Dysphagia- pharyngeal related to radiation, depending on the site of treatment.	
Nausea	none	able to eat	oral intake significantly decreased	no significant intake, requiring IV fluids	-	
Pancreatitis	none	-	-	abdominal pain with pancreatic enzyme elevation	complicated by shock (acute circulatory failure)	
Also consider Hypotension.	
Note: Asymptomatic amylase and Amylase are graded in the METABOLIC/LABORATORY category.	
Pharyngitis is graded in the GASTROINTESTINAL category as Stomatitis/pharyngitis (oral/pharyngeal mucositis).	
Proctitis	none	increased stool frequency, occasional blood-streaked stools, or rectal discomfort (including hemorrhoids), not requiring medication	increased stool frequency, bleeding, mucus discharge, or rectal discomfort requiring medication; anal fissure	increased stool frequency/diarrhea, requiring parenteral support; rectal bleeding, requiring transfusion; or persistent mucus discharge, necessitating pads	perforation, bleeding or necrosis or other life-threatening complication requiring surgical intervention (e.g., colostomy)	
Also consider Hemorrhage/bleeding with grade 3 or 4 thrombocytopenia, Hemorrhage/bleeding without grade 3 or 4 thrombocytopenia, and Pain due to radiation.	
Note:	Fistula is graded separately as Fistula- rectal/anal.	
Proctitis occurring more than 90 days after the start of radiation therapy is graded in the RTOG/EORTC Late Radiation Morbidity Scoring Scheme. (See Appendix IV)	
Salivary gland changes	none	slightly thickened saliva/may have slightly altered taste (e.g., metallic); additional fluids may be required	thick, ropy, sticky saliva; markedly altered taste; alteration in diet required	-	acute salivary gland necrosis	
Sense of smell	normal	slightly altered	markedly altered	-	-	
Stomatitis/pharyngitis (oral/pharyngeal mucositis)	none	painless ulcers, erythema, or mild soreness in the absence of lesions	painful erythema, edema, or ulcers, but can eat or swallow	painful erythema, edema, or ulcers requiring IV hydration	severe ulceration or requires parenteral or enteral nutritional support or prophylatic intubation	
For BMT:	none	painless ulcers, erythema, or mild soreness in the absence of lesions	painful erythema, edema or ulcers but can swallow	painful erythema, edema, or ulcers preventing swallowing or requiring hydration or parenteral (or enteral) nutritional support	severe ulceration requiring prophylactic intubation or resulting in documented aspiration pneumonia	
Note: Radiation-related mucositis is graded as Mucositis due to radiation.	
Taste disturbance (dysgeusia)	normal	slightly altered	markedly altered	-	-	
Typhlitis 
(inflammation of the cecum)	none	-	-	abdominal pain, diarrhea, fever, or radiographic documentation	perforation, bleeding or necrosis or other life-threatening complication requiring surgical intervention (e.g., colostomy)	
Also consider Hemorrhage/bleeding with grade 3 or 4 thrombocytopenia, Hemorrhage/bleeding without grade 3 or 4 thrombocytopenia, Hypotension, Febrile/neutropenia.	
Vomiting	none	1 episode in 24 hours over pretreatment	2-5 episodes in 24 hours over pretreatment	6 episodes in 24 hours over pretreatment; or need for IV fluids	Requiring parenteral nutrition; or physiologic consequences requiring intensive care; hemodynamic collapse	
Also consider Dehydration.	
Weight gain is graded in the CONSTITUTIONAL SYMPTOMS category.	
Weight loss is graded in the CONSTITUTIONAL SYMPTOMS category.	
Gastrointestinal-Other
(Specify, __________)	none	mild	moderate	severe	life-threatening or disabling	
HEMORRHAGE	
Note:	Transfusion in this section refers to pRBC infusion.	
For any bleeding with grade 3 or 4 platelets (< 50,000), always grade Hemorrhage/bleeding with grade 3 or 4 thrombocytopenia. Also consider platelets, transfusion- pRBCS, and transfusion-platelets in addition to the grade that incorporates the site or type of bleeding.	
If the site or type of hemorrhage/bleeding is listed, also use the grading that incorporates the site of bleeding: CNS hemorrhage/bleeding, Hematuria, Hematemesis, Hemoptysis, Hemorrhage/bleeding with surgery, Melena/lower GI bleeding, Petechiae/purpura (Hemorrhage/bleeding into skin), Rectal bleeding/hematochezia, Vaginal bleeding.	
If the platelet count is 50,000 and the site or type of bleeding is listed, grade the specific site. If the site or type is not listed and the platelet count is 50,000, grade Hemorrhage/bleeding without grade 3 or 4 thrombocytopenia and specify the site or type in the OTHER category.	
Hemorrhage/bleeding with grade 3 or 4 thrombocytopenia	none	mild without transfusion		requiring transfusion	catastrophic bleeding, requiring major non-elective intervention	
Also consider Platelets, Hemoglobin, Transfusion-platelet, Transfusion-pRBCs.	
Note:	This toxicity must be graded for any bleeding with grade 3 or 4 thrombocytopenia. Also grade the site or type of hemorrhage/bleeding. If the site is not listed, grade as Other in the HEMORRHAGE category.	
Hemorrhage/bleeding without grade 3 or 4 thrombocytopenia	none	mild without transfusion		requiring transfusion	catastrophic bleeding requiring major non-elective intervention	
Also consider Platelets, Hemoglobin, Transfusion-platelet, Transfusion-pRBCs.	
Note:	Bleeding in the absence of grade 3 or 4 thrombocytopenia is graded here only if the specific site or type of bleeding is not listed elsewhere in the HEMORRHAGE category. Also grade as Other in the HEMORRHAGE category.	
CNS hemorrhage/bleeding	none	-	-	bleeding noted on CT or other scan with no clinical consequences	hemorrhagic stroke or hemorrhagic vascular event (CVA) with neurologic signs and symptoms	
Epistaxis	none	mild without transfusion	-	requiring transfusion	catastrophic bleeding, requiring major non-elective intervention	
Hematemesis	none	mild without transfusion	-	requiring transfusion	catastrophic bleeding, requiring major non-elective intervention	
Hematuria 
(in the absence of vaginal bleeding)	none	microscopic only	intermittent gross bleeding, no clots	persistent gross bleeding or clots; may require catheterization or instrumentation, or transfusion	open surgery or necrosis or deep bladder ulceration	
Hemoptysis	none	mild without transfusion	-	requiring transfusion	catastrophic bleeding, requiring major non-elective intervention	
Hemorrhage/bleeding associated with surgery	none	mild without transfusion	-	requiring transfusion	catastrophic bleeding, requiring major non-elective intervention	
Note: Expected blood loss at the time of surgery is not graded as a toxicity.	
Melena/GI bleeding	none	mild without transfusion	-	requiring transfusion	catastrophic bleeding, requiring major non-elective intervention	
Petechiae/purpura (hemorrhage/bleeding into skin or mucosa)	none	rare petechiae of skin	petechiae or purpura in dependent areas of skin	generalized petechiae or purpura of skin or petechiae of any mucosal site	-	
Rectal bleeding/
hematochezia	none	mild without transfusion or medication	persistent, requiring medication (e.g., steroid suppositories) and/or break from radiation treatment	requiring transfusion	catastrophic bleeding, requiring major non-elective intervention	
Vaginal bleeding	none	spotting, requiring < 2 pads per day	requiring  2 pads per day, but not requiring transfusion	requiring transfusion	catastrophic bleeding, requiring major non-elective intervention	
Hemorrhage-Other 
(Specify site, ___________)	none	mild without transfusion	-	requiring transfusion	catastrophic bleeding, requiring major non-elective intervention	
HEPATIC	
Alkaline phosphatase	WNL	> ULN - 2.5 x ULN	> 2.5 - 5.0 x ULN	> 5.0 - 20.0 x ULN	> 20.0 x ULN	
Bilirubin	WNL	> ULN - 1.5 x ULN	> 1.5 - 3.0 x ULN	> 3.0 - 10.0 x ULN	> 10.0 x ULN	
Bilirubin- graft versus host disease (GVHD)	
Note: The following criteria are used only for bilirubin associated with graft versus host disease.	
	normal	2 - <3 mg/100 ml	3 - <6 mg/100 ml	6 - <15 mg/100 ml	15 mg/100 ml	
GGT
( - Glutamyl transpeptidase)	WNL	> ULN - 2.5 x ULN	> 2.5 - 5.0 x ULN	> 5.0 - 20.0 x ULN	> 20.0 x ULN	
Hepatic enlargement	absent	-	-	present	-	
Note: Grade Hepatic enlargement only for changes related to VOD or other treatment related toxicity.	
Hypoalbuminemia	WNL	<LLN - 3 g/dl	2 - <3 g/dl	<2 g/dl	-	
Liver dysfunction/failure (clinical)	normal	-	-	asterixis	encephalopathy or coma	
Note: Documented viral hepatitis is graded in the INFECTION category.	
Portal vein flow	normal	-	decreased portal vein flow	reversal/retrograde portal vein flow	-	
SGOT (AST) 
(serum glutamic oxaloacetic transaminase)	WNL	> ULN - 2.5 x ULN	> 2.5 - 5.0 x ULN	> 5.0 - 20.0 x ULN	> 20.0 x ULN	
SGPT (ALT) 
(serum glutamic pyruvic transaminase)	WNL	> ULN - 2.5 x ULN	> 2.5 - 5.0 x ULN	> 5.0 - 20.0 x ULN	> 20.0 x ULN	
Hepatic-Other 
(Specify, __________)	none	mild	moderate	severe	life-threatening or disabling	
INFECTION/FEBRILE NEUTROPENIA	
Catheter-related infection	none	mild, no active treatment	moderate, localized infection, requiring local or oral treatment	severe, systemic infection, requiring IV antibiotic or antifungal treatment or hospitalization	life-threatening sepsis (e.g., septic shock)	
Febrile neutropenia 
(fever of unknown origin without clinically or microbiologically documented infection)	none	-	-	Present	Life-threatening sepsis (e.g., septic shock)	
(ANC < 1.0 x 10^9^/L, fever 38.5°C)						
Note: Hypothermia instead of fever may be associated with neutropenia and is graded here.	
Infection (documented clinically or microbiologically) with grade 3 or 4 neutropenia	none	-	-	present	life-threatening sepsis (e.g., septic shock)	
(ANC < 1.0 x 10^9^/L)						
Note:	Hypothermia instead of fever may be associated with neutropenia and is graded here. In the absence of documented infection with grade 3 or 4 neutropenia, grade as Febrile neutropenia.	
Infection with unknown ANC	none	-	-	present	life-threatening sepsis (e.g., septic shock)	
Note: This toxicity criterion is used in the rare case when ANC is unknown.	
Infection without neutropenia	none	mild, no active treatment	moderate, localized infection, requiring local or oral treatment	severe, systemic infection, requiring IV antibiotic or antifungal treatment, or hospitalization	life-threatening sepsis (e.g., septic shock)	
Infection/Febrile Neutropenia-Other
(Specify, __________)	none	mild	moderate	severe	life-threatening or disabling	
Wound-infectious is graded in the DERMATOLOGY/SKIN category.	
LYMPHATICS	
Lymphatics	normal	mild lymphedema	moderate lymphedema requiring compression; lymphocyst	severe lymphedema limiting function; lymphocyst requiring surgery	severe lymphedema limiting function with ulceration	
Lymphatics-Other 
(Specify, __________)	none	mild	moderate	severe	life-threatening or disabling	
METABOLIC/LABORATORY	
Acidosis 
(metabolic or respiratory)	normal	pH < normal, but 7.3	-	pH < 7.3	pH < 7.3 with life-threatening physiologic consequences	
Alkalosis 
(metabolic or respiratory)	normal	pH > normal, but 7.5	-	pH > 7.5	pH > 7.5 with life-threatening physiologic consequences	
Amylase	WNL	> ULN - 1.5 x ULN	> 1.5 - 2.0 x ULN	> 2.0 - 5.0 x ULN	>5.0 x ULN	
Bicarbonate	WNL	< LLN - 16 mEq/dl	11 - 15 mEq/dl	8 - 10 mEq/dl	< 8 mEq/dl	
CPK
(creatine phosphokinase)	WNL	> ULN - 2.5 x ULN	> 2.5 - 5 x ULN	> 5 - 10 x ULN	> 10 x ULN	
Hypercalcemia	WNL	> ULN - 11.5 mg/dl
> ULN - 2.9 mmol/L	>11.5 - 12.5 mg/dl
> 2.9 - 3.1 mmol/L	>12.5 - 13.5 mg/dl
> 3.1 - 3.4 mmol/L	> 13.5 mg/dl
> 3.4 mmol/L	
Hypercholesterolemia	WNL	> ULN - 300 mg/dl
> ULN - 7.75 mmol/L	> 300 - 400 mg/dl
> 7.75 - 10.34 mmol/L	> 400 - 500 mg/dl
>10.34 - 12.92 mmol/L	> 500 mg/dl
> 12.92 mmol/L	
Hyperglycemia	WNL	> ULN - 160 mg/dl
> ULN - 8.9 mmol/L	> 160 - 250 mg/dl
> 8.9 - 13.9 mmol/L	> 250 - 500 mg/dl
> 13.9 - 27.8 mmol/L	> 500 mg/dl
> 27.8 mmol/L or ketoacidosis	
Hyperkalemia	WNL	> ULN - 5.5 mmol/L	> 5.5 - 6.0 mmol/L	> 6.0 - 7.0 mmol/L	> 7.0 mmol/L	
Hypermagnesemia	WNL	> ULN - 3.0 mg/dl
> ULN - 1.23 mmol/L	-	> 3.0 - 8.0 mg/dl
> 1.23 - 3.30 mmol/L	> 8.0 mg/dl
> 3.30 mmol/L	
Hypernatremia	WNL	> ULN - 150 mmol/L	>150 - 155 mmol/L	>155 - 160 mmol/L	>160 mmol/L	
Hypertriglyceridemia	WNL	> ULN - 2.5 x ULN	> 2.5 - 5.0 x ULN	> 5.0 - 10 x ULN	> 10 x ULN	
Hyperuricemia	WNL	> ULN -  10 mg/dl
 0.59 mmol/L without physiologic consequences	-	> ULN -  10 mg/dl
 0.59 mmol/L with physiologic consequences	> 10 mg/dl
> 0.59 mmol/L	
Also consider Tumor lysis syndrome, Renal failure, Creatinine, Potassium.	
Hypocalcemia	WNL	<LLN - 8.0 mg/dl
<LLN - 2.0 mmol/L	7.0 - < 8.0 mg/dl
1.75 - < 2.0 mmol/L	6.0 - < 7.0 mg/dl
1.5 - < 1.75 mmol/L	<6.0 mg/dl
< 1.5 mmol/L	
Hypoglycemia	WNL	<LLN - 55 mg/dl
<LLN - 3.0 mmol/L	40 - < 55 mg/dl
2.2 - < 3.0 mmol/L	30 - < 40 mg/dl
1.7 - < 2.2 mmol/L	< 30 mg/dl
< 1.7 mmol/L	
Hypokalemia	WNL	<LLN - 3.0 mmol/L	-	2.5 - <3.0 mmol/L	<2.5 mmol/L	
Hypomagnesemia	WNL	<LLN - 1.2 mg/dl
<LLN - 0.5 mmol/L	0.9 - <1.2 mg/dl
0.4 - < 0.5 mmol/L	0.7 - < 0.9 mg/dl
0.3 - < 0.4 mmol/L	< 0.7 mg/dl
< 0.3 mmol/L	
Hyponatremia	WNL	<LLN - 130 mmol/L	-	120 - <130 mmol/L	<120 mmol/L	
Hypophosphatemia	WNL	<LLN -2.5 mg/dl
<LLN - 0.8 mmol/L	2.0 - <2.5 mg/dl
0.6 - <0.8 mmol/L	1.0 - <2.0 mg/dl
0.3 - <0.6 mmol/L	< 1.0 mg/dl
<0.3 mmol/L	
Hypothyroidism is graded in the ENDOCRINE category.	
Lipase	WNL	> ULN - 1.5 x ULN	> 1.5 - 2.0 x ULN	> 2.0 - 5.0 x ULN	> 5.0 x ULN	
Metabolic/Laboratory-Other (Specify, __________)	none	mild	moderate	severe	life-threatening or disabling	
MUSCULOSKELETAL	
Arthralgia is graded in the PAIN category.	
Arthritis	none	mild pain with inflammation, erythema or joint swelling but not interfering with function	moderate pain with inflammation, erythema, or joint swelling interfering with function, but not interfering with activities of daily living	severe pain with inflammation, erythema, or joint swelling and interfering with activities of daily living	disabling	
Muscle weakness
(not due to neuropathy)	normal	asymptomatic with weakness on physical exam	symptomatic and interfering with function, but not interfering with activities of daily living	symptomatic and interfering with activities of daily living	bedridden or disabling	
Myalgia is graded in the PAIN category.	
Myositis (inflammation/damage of muscle)	none	mild pain, not interfering with function	pain interfering with function, but not interfering with activities of daily living	pain interfering with function and interfering with activities of daily living	bedridden or disabling	
Also consider CPK.	
Note: Myositis implies muscle damage (i.e., elevated CPK).	
Osteonecrosis 
(avascular necrosis)	none	asymptomatic and detected by imaging only	symptomatic and interfering with function, but not interfering with activities of daily living	symptomatic and interfering with activities of daily living	symptomatic; or disabling	
Musculoskeletal-Other
(Specify, __________)	none	mild	moderate	severe	life-threatening or disabling	
NEUROLOGY	
Aphasia, receptive and/or expressive, is graded under Speech impairment in the NEUROLOGY category.	
Arachnoiditis/meningismus/
radiculitis	absent	mild pain not interfering with function	moderate pain interfering with function, but not interfering with activities of daily living	severe pain interfering with activities of daily living	unable to function or perform activities of daily living; bedridden; paraplegia	
Also consider Headache, Vomiting, Fever.	
Ataxia (incoordination)	normal	asymptomatic but abnormal on physical exam, and not interfering with function	mild symptoms interfering with function, but not interfering with activities of daily living	moderate symptoms interfering with activities of daily living	bedridden or disabling	
CNS cerebrovascular ischemia	none	-	-	transient ischemic event or attack (TIA)	permanent event (e.g., cerebral vascular accident)	
CNS hemorrhage/bleeding is graded in the HEMORRHAGE category.	
Cognitive disturbance/ learning problems	none	cognitive disability; not interfering with work/school performance; preservation of intelligence	cognitive disability; interfering with work/school performance; decline of 1 SD (Standard Deviation) or loss of developmental milestones	cognitive disability; resulting in significant impairment of work/school performance; cognitive decline > 2 SD	inability to work/frank mental retardation	
Confusion	normal	confusion or disorientation or attention deficit of brief duration; resolves spontaneously with no sequelae	confusion or disorientation or attention deficit interfering with function, but not interfering with activities of daily living	confusion or delirium interfering with activities of daily living	harmful to others or self; requiring hospitalization	
Cranial neuropathy is graded in the NEUROLOGY category as Neuropathy-cranial.	
Delusions	normal	-	-	present	toxic psychosis	
Depressed level of consciousness	normal	somnolence or sedation not interfering with function	somnolence or sedation interfering with function, but not interfering with activities of daily living	obtundation or stupor; difficult to arouse; interfering with activities of daily living	coma	
Note: Syncope (fainting) is graded in the NEUROLOGY category.	
Dizziness/lightheadedness	none	not interfering with function	interfering with function, but not interfering with activities of daily living	interfering with activities of daily living	bedridden or disabling	
Dysphasia, receptive and/or expressive, is graded under Speech impairment in the NEUROLOGY category.	
Extrapyramidal/ 
involuntary movement/ restlessness	none	mild involuntary movements not interfering with function	moderate involuntary movements interfering with function, but not interfering with activities of daily living	severe involuntary movements or torticollis interfering with activities of daily living	bedridden or disabling	
Hallucinations	normal	-	-	present	toxic psychosis	
Headache is graded in the PAIN category.	
Insomnia	normal	occasional difficulty sleeping not interfering with function	difficulty sleeping interfering with function, but not interfering with activities of daily living	frequent difficulty sleeping, interfering with activities of daily living	-	
Note: This toxicity is graded when insomnia is related to treatment. If pain or other symptoms interfere with sleep do NOT grade as insomnia.	
Irritability
(children <3 years of age)	normal	mild; easily consolable	moderate; requiring increased attention	severe; inconsolable	-	
Leukoencephalopathy associated radiological findings	none	mild increase in SAS (subarachnoid space) and/or mild ventriculomegaly; and/or small (+/- multiple) focal T2 hyperintensities, involving periventricular white matter or < 1/3 of susceptible areas of cerebrum	moderate increase in SAS; and/or moderate ventriculomegaly; and/or focal T2 hyperintensities extending into centrum ovale; or involving 1/3 to 2/3 of susceptible areas of cerebrum	severe increase in SAS; severe ventriculomegaly; near total white matter T2 hyperintensities or diffuse low attenuation (CT); focal white matter necrosis (cystic)	severe increase in SAS; severe ventriculomegaly; diffuse low attenuation with calcification (CT); diffuse white matter necrosis (MRI)	
Memory loss	normal	memory loss not interfering with function	memory loss interfering with function, but not interfering with activities of daily living	memory loss interfering with activities of daily living	amnesia	
Mood alteration- anxiety agitation	normal	mild mood alteration not interfering with function	moderate mood alteration interfering with function, but not interfering with activities of daily living	severe mood alteration interfering with activities of daily living	suicidal ideation or danger to self	
Mood alteration- depression	normal	mild mood alteration not interfering with function	moderate mood alteration interfering with function, but not interfering with activities of daily living	severe mood alteration interfering with activities of daily living	suicidal ideation or danger to self	
Mood alteration- euphoria	normal	mild mood alteration not interfering with function	moderate mood alteration interfering with function, but not interfering with activities of daily living	severe mood alteration interfering with activities of daily living	danger to self	
Neuropathic pain is graded in the PAIN category.	
Neuropathy- cranial	absent	-	present, not interfering with activities of daily living	present, interfering with activities of daily living	life-threatening, disabling	
Neuropathy- motor	normal	subjective weakness but no objective findings	mild objective weakness interfering with function, but not interfering with activities of daily living	objective weakness interfering with activities of daily living	paralysis	
Neuropathy-sensory	normal	loss of deep tendon reflexes or paresthesia (including tingling) but not interfering with function	objective sensory loss or paresthesia (including tingling), interfering with function, but not interfering with activities of daily living	sensory loss or paresthesia interfering with activities of daily living	permanent sensory loss that interferes with function	
Nystagmus	absent	present	-	-	-	
Also consider Vision-double vision.	
Personality/behavioral	normal	change, but not disruptive to patient or family	disruptive to patient or family	disruptive to patient and family; requiring mental health intervention	harmful to others or self; requiring hospitalization	
Pyramidal tract dysfunction (e.g.,  tone, hyperreflexia, positive Babinski,  fine motor coordination)	normal	asymptomatic with abnormality on physical examination	symptomatic or interfering with function but not interfering with activities of daily living	interfering with activities of daily living	bedridden or disabling; paralysis	
Seizure(s)	none	-	seizure(s) self-limited and consciousness is preserved	seizure(s) in which consciousness is altered	seizures of any type which are prolonged, repetitive, or difficult to control (e.g., status epilepticus, intractable epilepsy)	
Speech impairment 
(e.g., dysphasia or aphasia)	normal	-	awareness of receptive or expressive dysphasia, not impairing ability to communicate	receptive or expressive dysphasia, impairing ability to communicate	inability to communicate	
Syncope (fainting)	absent	-	-	present	-	
Also consider CARDIOVASCULAR (ARRHYTHMIA), Vasovagal episode, CNS cerebrovascular ischemia.	
Tremor	none	mild and brief or intermittent but not interfering with function	moderate tremor interfering with function, but not interfering with activities of daily living	severe tremor interfering with activities of daily living	-	
Vertigo	none	not interfering with function	interfering with function, but not interfering with activities of daily living	interfering with activities of daily living	bedridden or disabling	
Neurology-Other
(Specify, __________)	none	mild	moderate	severe	life-threatening or disabling	
OCULAR/VISUAL	
Cataract	none	asymptomatic	symptomatic, partial visual loss	symptomatic, visual loss requiring treatment or interfering with function	-	
Conjunctivitis	none	abnormal ophthalmologic changes, but asymptomatic or symptomatic without visual impairment (i.e., pain and irritation)	symptomatic and interfering with function, but not interfering with activities of daily living	symptomatic and interfering with activities of daily living	-	
Dry eye	normal	mild, not requiring treatment	moderate or requiring artificial tears	-	-	
Glaucoma	none	increase in intraocular pressure but no visual loss	increase in intraocular pressure with retinal changes	visual impairment	unilateral or bilateral loss of vision (blindness)	
Keratitis 
(corneal inflammation/ corneal ulceration)	none	abnormal ophthalmologic changes but asymptomatic or symptomatic without visual impairment (i.e., pain and irritation)	symptomatic and interfering with function, but not interfering with activities of daily living	symptomatic and interfering with activities of daily living	unilateral or bilateral loss of vision (blindness)	
Tearing (watery eyes)	none	mild: not interfering with function	moderate: interfering with function, but not interfering with activities of daily living	interfering with activities of daily living	-	
Vision- blurred vision	normal	-	symptomatic and interfering with function, but not interfering with activities of daily living	symptomatic and interfering with activities of daily living	-	
Vision- double vision (diplopia)	normal	-	symptomatic and interfering with function, but not interfering with activities of daily living	symptomatic and interfering with activities of daily living	-	
Vision- flashing lights/floaters	normal	mild, not interfering with function	symptomatic and interfering with function, but not interfering with activities of daily living	symptomatic and interfering with activities of daily living	-	
Vision- night blindness (nyctalopia)	normal	abnormal electro-retinography but asymptomatic	symptomatic and interfering with function, but not interfering with activities of daily living	symptomatic and interfering with activities of daily living	-	
Vision- photophobia	normal	-	symptomatic and interfering with function, but not interfering with activities of daily living	symptomatic and interfering with activities of daily living	-	
Ocular/Visual-Other 
(Specify, __________)	normal	mild	moderate	severe	unilateral or bilateral loss of vision (blindness)	
PAIN	
Abdominal pain or cramping	none	mild pain not interfering with function	moderate pain: pain or analgesics interfering with function, but not interfering with activities of daily living	severe pain: pain or analgesics severely interfering with activities of daily living	disabling	
Arthralgia 
(joint pain)	none	mild pain not interfering with function	moderate pain: pain or analgesics interfering with function, but not interfering with activities of daily living	severe pain: pain or analgesics severely interfering with activities of daily living	disabling	
Arthritis (joint pain with clinical signs of inflammation) is graded in the MUSCULOSKELETAL category.	
Bone pain	none	mild pain not interfering with function	moderate pain: pain or analgesics interfering with function, but not interfering with activities of daily living	severe pain: pain or analgesics severely interfering with activities of daily living	disabling	
Chest pain 
(non-cardiac and non-pleuritic)	none	mild pain not interfering with function	moderate pain: pain or analgesics interfering with function, but not interfering with activities of daily living	severe pain: pain or analgesics severely interfering with activities of daily living	disabling	
Dysmenorrhea	none	mild pain not interfering with function	moderate pain: pain or analgesics interfering with function, but not interfering with activities of daily living	severe pain: pain or analgesics severely interfering with activities of daily living	disabling	
Dyspareunia	none	mild pain not interfering with function	moderate pain interfering with sexual activity	severe pain preventing sexual activity	-	
Dysuria is graded in the RENAL/GENITOURINARY category.	
Earache (otalgia)	none	mild pain not interfering with function	moderate pain: pain or analgesics interfering with function, but not interfering with activities of daily living	severe pain: pain or analgesics severely interfering with activities of daily living	disabling	
Headache	none	mild pain not interfering with function	moderate pain: pain or analgesics interfering with function, but not interfering with activities of daily living	severe pain: pain or analgesics severely interfering with activities of daily living	disabling	
Hepatic pain	none	mild pain not interfering with function	moderate pain: pain or analgesics interfering with function, but not interfering with activities of daily living	severe pain: pain or analgesics severely interfering with activities of daily living	disabling	
Myalgia 
(muscle pain)	none	mild pain not interfering with function	moderate pain: pain or analgesics interfering with function, but not interfering with activities of daily living	severe pain: pain or analgesics severely interfering with activities of daily living	disabling	
Neuropathic pain 
(e.g., jaw pain, neurologic pain, phantom limb pain, post-infectious neuralgia, or painful neuropathies)	none	mild pain not interfering with function	moderate pain: pain or analgesics interfering with function, but not interfering with activities of daily living	severe pain: pain or analgesics severely interfering with activities of daily living	disabling	
Pain due to radiation	none	mild pain not interfering with function	moderate pain: pain or analgesics interfering with function, but not interfering with activities of daily living	severe pain: pain or analgesics severely interfering with activities of daily living	disabling	
Pelvic pain	none	mild pain not interfering with function	moderate pain: pain or analgesics interfering with function, but not interfering with activities of daily living	severe pain: pain or analgesics severely interfering with activities of daily living	disabling	
Pleuritic pain	none	mild pain not interfering with function	moderate pain: pain or analgesics interfering with function, but not interfering with activities of daily living	severe pain: pain or analgesics severely interfering with activities of daily living	disabling	
Rectal or perirectal pain (proctalgia)	none	mild pain not interfering with function	moderate pain: pain or analgesics interfering with function, but not interfering with activities of daily living	severe pain: pain or analgesics severely interfering with activities of daily living	disabling	
Tumor pain 
(onset or exacerbation of tumor pain due to treatment)	none	mild pain not interfering with function	moderate pain: pain or analgesics interfering with function, but not interfering with activities of daily living	severe pain: pain or analgesics severely interfering with activities of daily living	disabling	
Tumor flair is graded in the SYNDROME category.	
Pain-Other
(Specify, __________)	none	mild	moderate	severe	disabling	
PULMONARY	
Adult Respiratory Distress Syndrome (ARDS)	absent	-	-	-	present	
Apnea	none	-	-	present	requiring intubation	
Carbon monoxide diffusion capacity (DLCO)	 90% of pretreatment or normal value	75 - <90% of pretreatment or normal value	50 - <75% of pretreatment or normal value	25 - <50% of pretreatment or normal value	< 25% of pretreatment or normal value	
Cough	absent	mild, relieved by non-prescription medication	requiring narcotic antitussive	severe cough or coughing spasms, poorly controlled or unresponsive to treatment	-	
Dyspnea 
(shortness of breath)	normal	-	dyspnea on exertion	dyspnea at normal level of activity	dyspnea at rest or requiring ventilator support	
FEV1	 90% of pretreatment or normal value	75 - <90% of pretreatment or normal value	50 - <75% of pretreatment or normal value	25 - <50% of pretreatment or normal value	< 25% of pretreatment or normal value	
Hiccoughs (hiccups, singultus)	none	mild, not requiring treatment	moderate, requiring treatment	severe, prolonged, and refractory to treatment	-	
Hypoxia	normal	-	decreased O2 saturation with exercise	decreased O2 saturation at rest, requiring supplemental oxygen	decreased O2 saturation, requiring pressure support (CPAP) or assisted ventilation	
Pleural effusion 
(non-malignant)	none	asymptomatic and not requiring treatment	symptomatic, requiring diuretics	symptomatic, requiring O2 or therapeutic thoracentesis	life-threatening (e.g., requiring intubation)	
Pleuritic pain is graded in the PAIN category.	
Pneumonitis/pulmonary infiltrates	none	radiographic changes but asymptomatic or symptoms not requiring steroids	radiographic changes and requiring steroids or diuretics	radiographic changes and requiring oxygen	radiographic changes and requiring assisted ventilation	
Pneumothorax	none	no intervention required	chest tube required	sclerosis or surgery required	life-threatening	
Pulmonary embolism is graded as Thrombosis/embolism in the CARDIOVASCULAR (GENERAL) category.	
Pulmonary fibrosis	none	radiographic changes, but asymptomatic or symptoms not requiring steroids	requiring steroids or diuretics	requiring oxygen	requiring assisted ventilation	
Note: Radiation-related pulmonary fibrosis is graded in the RTOG/EORTC Late Radiation Morbidity Scoring Scheme- Lung. (See Appendix IV)	
Voice changes/stridor/larynx (e.g., hoarseness, loss of voice, laryngitis)	normal	mild or intermittent hoarseness	persistent hoarseness, but able to vocalize; may have mild to moderate edema	whispered speech, not able to vocalize; may have marked edema	marked dyspnea/stridor requiring tracheostomy or intubation	
Note:	Cough from radiation is graded as cough in the PULMONARY category.	
Radiation-related hemoptysis from larynx/pharynx is graded as Grade 4 Mucositis due to radiation in the GASTROINTESTINAL category.  Radiation-related hemoptysis from the thoracic cavity is graded as Grade 4 Hemoptysis in the HEMORRHAGE category.	
Pulmonary-Other
(Specify, __________)	none	mild	moderate	severe	life-threatening or disabling	
RENAL/GENITOURINARY	
Bladder spasms	absent	mild symptoms, not requiring intervention	symptoms requiring antispasmotic	severe symptoms requiring narcotic	-	
Creatinine	WNL	> ULN - 1.5 x ULN	> 1.5 - 3.0 x ULN	> 3.0 - 6.0 x ULN	> 6.0 x ULN	
Note: Adjust to age-appropriate levels for pediatric patients.	
Dysuria 
(painful urination)	none	mild symptoms requiring no intervention	symptoms relieved with therapy	symptoms not relieved despite therapy	-	
Fistula or GU fistula 
(e.g., vaginal, vesicovaginal)	none	-	-	requiring intervention	requiring surgery	
Hemoglobinuria	-	present	-	-	-	
Hematuria (in the absence of vaginal bleeding) is graded in the HEMORRHAGE category.	
Incontinence	none	with coughing, sneezing, etc.	spontaneous, some control	no control (in the absence of fistula)	-	
Operative injury to bladder and/or ureter	none	-	injury of bladder with primary repair	sepsis, fistula, or obstruction requiring secondary surgery; loss of one kidney; injury requiring anastomosis or re-implantation	septic obstruction of both kidneys or vesicovaginal fistula requiring diversion	
Proteinuria	normal or < 0.15 g/24 hours	1+ or 0.15 - 1.0 g/24 hours	2+ to 3+ or 1.0 - 3.5 g/24 hours	4+ or > 3.5 g/24 hours	nephrotic syndrome	
Note: If there is an inconsistency between absolute value and uristix reading, use the absolute value for grading.	
Renal failure	none	-	-	requiring dialysis, but reversible	requiring dialysis and irreversible	
Ureteral obstruction	none	unilateral, not requiring surgery	-	bilateral, not requiring surgery	stent, nephrostomy tube, or surgery	
Urinary electrolyte wasting (e.g., Fanconi's syndrome, renal tubular acidosis)	none	asymptomatic, not requiring treatment	mild, reversible and manageable with oral replacement	reversible but requiring IV replacement	irreversible, requiring continued replacement	
Also consider Acidosis, Bicarbonate, Hypocalcemia, Hypophosphatemia.	
Urinary frequency/urgency	normal	increase in frequency or nocturia up to 2 x normal	increase > 2 x normal but < hourly	hourly or more with urgency, or requiring catheter	-	
Urinary retention	normal	hesitancy or dribbling, but no significant residual urine; retention occurring during the immediate postoperative period	hesitancy requiring medication or occasional in/out catheterization (<4 x per week), or operative bladder atony requiring indwelling catheter beyond immediate postoperative period but for < 6 weeks	requiring frequent in/out catheterization 
( 4 x per week) or urological intervention (e.g., TURP, suprapubic tube, urethrotomy)	bladder rupture	
Urine color change 
(not related to other dietary or physiologic cause e.g., bilirubin, concentrated urine, hematuria)	normal	asymptomatic, change in urine color	-	-	-	
Vaginal bleeding is graded in the HEMORRHAGE category.	
Vaginitis 
(not due to infection)	none	mild, not requiring treatment	moderate, relieved with treatment	severe, not relieved with treatment, or ulceration not requiring surgery	ulceration requiring surgery	
Renal/Genitourinary-Other (Specify, __________)	none	mild	moderate	severe	life-threatening or disabling	
SECONDARY MALIGNANCY	
Secondary Malignancy-Other 
(Specify type, __________) excludes metastastic tumors	none	-	-	-	present	
SEXUAL/REPRODUCTIVE FUNCTION	
Dyspareunia is graded in the PAIN category.	
Dysmenorrhea is graded in the PAIN category.	
Erectile impotence	normal	mild (erections impaired but satisfactory)	moderate (erections impaired, unsatisfactory for intercourse)	no erections	-	
Female sterility	normal	-	-	sterile	-	
Femininization of male is graded in the ENDOCRINE category.	
Irregular menses 
(change from baseline)	normal	occasionally irregular or lengthened interval, but continuing menstrual cycles	very irregular, but continuing menstrual cycles	persistent amenorrhea	-	
Libido	normal	decrease in interest	severe loss of interest	-	-	
Male infertility	-	-	Oligospermia 
(low sperm count)	Azoospermia 
(no sperm)	-	
Masculinization of female is graded in the ENDOCRINE category.	
Vaginal dryness	normal	mild	requiring treatment and/or interfering with sexual function, dyspareunia	-	-	
Sexual/Reproductive Function-Other
(Specify, __________)	none	mild	moderate	severe	disabling	
SYNDROMES (not included in previous categories)	
Acute vascular leak syndrome is graded in the CARDIOVASCULAR (GENERAL) category.	
ARDS (Adult Respiratory Distress Syndrome) is graded in the PULMONARY category.	
Autoimmune reactions are graded in the ALLERGY/IMMUNOLOGY category.	
DIC (disseminated intravascular coagulation) is graded in the COAGULATION category.	
Fanconi's syndrome is graded as Urinary electrolyte wasting in the RENAL/GENITOURINARY category.	
Renal tubular acidosis is graded as Urinary electrolyte wasting in the RENAL/GENITOURINARY category.	
Stevens-Johnson syndrome (erythema multiforme) is graded in the DERMATOLOGY/SKIN category.	
SIADH (syndrome of inappropriate antidiuretic hormone) is graded in the ENDOCRINE category.	
Thrombotic microangiopathy (e.g., thromboitic thrombocytopenic purpura/TTP or hemolytic uremic syndrom/HUS) is graded in the COAGULATION category.	
Tumor flare	none	mild pain not interfering with function	moderate pain; pain or analgesics interfering with function, but not interfering with activities of daily living	severe pain; pain or analgesics interfering with function and interfering with activities of daily living	Disabling	
Also consider Hypercalcemia.	
Note:	Tumor flare is characterized by a constellation of symptoms and signs in direct relation to initiation of therapy (e.g., anti-estrogens/androgens or additional hormones). The symptoms/signs include tumor pain, inflammation of visible tumor, hypercalcemia, diffuse bone pain, and other electrolyte disturbances.	
Tumor lysis syndrome	absent	-	-	present	-	
Also consider Hyperkalemia, Creatinine.	
Urinary electrolyte wasting (e.g., Fanconi's syndrome, renal tubular acidosis) is graded under the RENAL/GENITOURINARY category.	
Syndromes-Other
(Specify, __________)	none	mild	moderate	severe	life-threatening or disabling	

17.4.	APPENDIX VI: STUDY   INCLUSION FORM
Study INN 06 Inclusion FAX

Please fax this form to:
Univ. Prof. Dr. W. Hilbe
Fax:		0512-504-23431
Tel:		0512-504-23333
Institution:	Medical University Innsbruck
Address:	Anichstr. 35
		A-6020 Innsbruck

Name of investigator:
Center:
Patient's initials:  				Date of birth:
			
		


Patient meets all inclusion/exclucion criteria
Yes					No	

Written informed consent obtained
Yes					No	

Date informed consent signed:
			


Date of first planned induction chemotherapy:
			


Date:						Investigator's or designee's signature:
						


		
Patient No:


Date:						Designee's signiture:


 
17.5.	APPENDIX VII: SERIOUS ADVERSE EVENT REPORT FORM


           
    


Please fax attached form to Study Center, Fax No.: 0512 504 23431
	


17.6.	APPENDIX VIII: WORLD MEDICAL ASSOCIATION DECLARATION OF HELSINKI
WORLD MEDICAL ASSOCIATION DECLARATION OF HELSINKI	
Ethical Principles for Medical Research Involving Human Subjects
Adopted by the 18th WMA General Assembly
Helsinki, Finland, June 1964
and amended by the
29th WMA General Assembly, Tokyo, Japan, October 1975
35th WMA General Assembly, Venice, Italy, October 1983
41st WMA General Assembly, Hong Kong, September 1989
48th WMA General Assembly, Somerset West, Republic of South Africa, October 1996
and the 52nd WMA General Assembly, Edinburgh, Scotland, October 2000 	
A.	INTRODUCTION	
1. 	The World Medical Association has developed the Declaration of Helsinki as a statement of ethical principles to provide guidance to physicians and other participants in medical research involving human subjects. Medical research involving human subjects includes research on identifiable human material or identifiable data.	
		
2.	It is the duty of the physician to promote and safeguard the health of the people. The physician's knowledge and conscience are dedicated to the fulfillment of this duty.	
3.	The Declaration of Geneva of the World Medical Association binds the physician with the words, "The health of my patient will be my first consideration," and the International Code of Medical Ethics declares that, "A physician shall act only in the patient's interest when providing medical care which might have the effect of weakening the physical and mental condition of the patient." 	
4.	Medical progress is based on research which ultimately must rest in part on experimentation involving human subjects.	
5.	In medical research on human subjects, considerations related to the well-being of the human subject should take precedence over the interests of science and society.	
6.	The primary purpose of medical research involving human subjects is to improve prophylactic, diagnostic and therapeutic procedures and the understanding of the aetiology and pathogenesis of disease. Even the best proven prophylactic, diagnostic, and therapeutic methods must continuously be challenged through research for their effectiveness, efficiency, accessibility and quality. 	
7.	In current medical practice and in medical research, most prophylactic, diagnostic and therapeutic procedures involve risks and burdens. 	
8.	Medical research is subject to ethical standards that promote respect for all human beings and protect their health and rights. Some research populations are vulnerable and need special protection. The particular needs of the economically and medically disadvantaged must be recognized. Special attention is also required for those who cannot give or refuse consent for themselves, for those who may be subject to giving consent under duress, for those who will not benefit personally from the research and for those for whom the research is combined with care. 	
9.	Research Investigators should be aware of the ethical, legal and regulatory requirements for research on human subjects in their own countries as well as applicable international requirements. No national ethical, legal or regulatory requirement should be allowed to reduce or eliminate any of the protections for human subjects set forth in this Declaration.	
B.	BASIC PRINCIPLES FOR ALL MEDICAL RESEARCH	
10. 	It is the duty of the physician in medical research to protect the life, health, privacy, and dignity of the human subject. 	
11.	Medical research involving human subjects must conform to generally accepted scientific principles, be based on a thorough knowledge of the scientific literature, other relevant sources of information, and on adequate laboratory and, where appropriate, animal experimentation.	
12.	Appropriate caution must be exercised in the conduct of research which may affect the environment, and the welfare of animals used for research must be respected.
	
13.	The design and performance of each experimental procedure involving human subjects should be clearly formulated in an experimental protocol. This protocol should be submitted for consideration, comment, guidance, and where appropriate, approval to a specially appointed ethical review committee, which must be independent of the investigator, the sponsor or any other kind of undue influence. This independent committee should be in conformity with the laws and regulations of the country in which the research experiment is performed. The committee has the right to monitor ongoing trials. The researcher has the obligation to provide monitoring information to the committee, especially any serious adverse events. The researcher should also submit to the committee, for review, information regarding funding, sponsors, institutional affiliations, other potential conflicts of interest and incentives for subjects. 	
14.	The research protocol should always contain a statement of the ethical considerations involved and should indicate that there is compliance with the principles enunciated in this Declaration. 	
15.	Medical research involving human subjects should be conducted only by scientifically qualified persons and under the supervision of a clinically competent medical person. The responsibility for the human subject must always rest with a medically qualified person and never rest on the subject of the research, even though the subject has given consent.	
16.	Every medical research project involving human subjects should be preceded by careful assessment of predictable risks and burdens in comparison with foreseeable benefits to the subject or to others. This does not preclude the participation of healthy volunteers in medical research. The design of all studies should be publicly available.	
17.	Physicians should abstain from engaging in research projects involving human subjects unless they are confident that the risks involved have been adequately assessed and can be satisfactorily managed. Physicians should cease any investigation if the risks are found to outweigh the potential benefits or if there is conclusive proof of positive and beneficial results.	
18.	Medical research involving human subjects should only be conducted if the importance of the objective outweighs the inherent risks and burdens to the subject. This is especially important when the human subjects are healthy volunteers. 	
19.	Medical research is only justified if there is a reasonable likelihood that the populations in which the research is carried out stand to benefit from the results of the research. 	
20.	The subjects must be volunteers and informed participants in the research project.	
21.	The right of research subjects to safeguard their integrity must always be respected. Every precaution should be taken to respect the privacy of the subject, the confidentiality of the patient's information and to minimize the impact of the study on the subject's physical and mental integrity and on the personality of the subject.	
22.	In any research on human beings, each potential subject must be adequately informed of the aims, methods, sources of funding, any possible conflicts of interest, institutional affiliations of the researcher, the anticipated benefits and potential risks of the study and the discomfort it may entail. The subject should be informed of the right to abstain from participation in the study or to withdraw consent to participate at any time without reprisal. After ensuring that the subject has understood the information, the physician should then obtain the subject's freely-given informed consent, preferably in writing. If the consent cannot be obtained in writing, the non-written consent must be formally documented and witnessed. 	
23.	When obtaining informed consent for the research project the physician should be particularly cautious if the subject is in a dependent relationship with the physician or may consent under duress. In that case the informed consent should be obtained by a well-informed physician who is not engaged in the investigation and who is completely independent of this relationship. 	
24.	For a research subject who is legally incompetent, physically or mentally incapable of giving consent or is a legally incompetent minor, the investigator must obtain informed consent from the legally authorized representative in accordance with applicable law. These groups should not be included in research unless the research is necessary to promote the health of the population represented and this research cannot instead be performed on legally competent persons. 	
25.	When a subject deemed legally incompetent, such as a minor child, is able to give assent to decisions about participation in research, the investigator must obtain that assent in addition to the consent of the legally authorized representative.	
26.	Research on individuals from whom it is not possible to obtain consent, including proxy or advance consent, should be done only if the physical/mental condition that prevents obtaining informed consent is a necessary characteristic of the research population. The specific reasons for involving research subjects with a condition that renders them unable to give informed consent should be stated in the experimental protocol for consideration and approval of the review committee. The protocol should state that consent to remain in the research should be obtained as soon as possible from the individual or a legally authorized surrogate.	
27.	Both authors and publishers have ethical obligations. In publication of the results of research, the investigators are obliged to preserve the accuracy of the results. Negative as well as positive results should be published or otherwise publicly available. Sources of funding, institutional affiliations and any possible conflicts of interest should be declared in the publication. Reports of experimentation not in accordance with the principles laid down in this Declaration should not be accepted for publication. 	
C.	ADDITIONAL PRINCIPLES FOR MEDICAL RESEARCH COMBINED WITH MEDICAL CARE 	
		
28.	The physician may combine medical research with medical care, only to the extent that the research is justified by its potential prophylactic, diagnostic or therapeutic value. When medical research is combined with medical care, additional standards apply to protect the patients who are research subjects.	
29.	The benefits, risks, burdens and effectiveness of a new method should be tested against those of the best current prophylactic, diagnostic, and therapeutic methods. This does not exclude the use of placebo, or no treatment, in studies where no proven prophylactic, diagnostic or therapeutic method exists.	
30.	At the conclusion of the study, every patient entered into the study should be assured of access to the best proven prophylactic, diagnostic and therapeutic methods identified by the study.	
31.	The physician should fully inform the patient which aspects of the care are related to the research. The refusal of a patient to participate in a study must never interfere with the patient-physician relationship.	
32.	In the treatment of a patient, where proven prophylactic, diagnostic and therapeutic methods do not exist or have been ineffective, the physician, with informed consent from the patient, must be free to use unproven or new prophylactic, diagnostic and therapeutic measures, if in the physician's judgement it offers hope of saving life, re-establishing health or alleviating suffering. Where possible, these measures should be made the object of research, designed to evaluate their safety and efficacy. In all cases, new information should be recorded and, where appropriate, published. The other relevant guidelines of this Declaration should be followed.	
		
		
		
		
		
		


17.7.	APPENDIX IX: PATIENT INFORMATION AND INFORMED CONSENT 

PATIENTENINFORMATION UND EINVERSTÄNDNISERKLÄRUNG

Multizentrische Phase II-Studie zur Beurteilung von Docetaxel, Cisplatin und Cetuximab als Induktionschemotherapie vor Operation bei Patienten mit nicht-kleinzelligem Bronchialkarzinom (NSCLC) Stadium IB, II und IIIA  

S e h r  g e e h r t e  P a t i e n t i n,  s e h r  g e e h r t e r  P a t i e n t !

Ihr Arzt hat mit Ihnen besprochen, dass Sie an Lungenkrebs erkrankt sind und nun der Tumor operativ entfernt werden sollte.  
Die Standardtherapie besteht derzeit in einer Operation mit anschließender Strahlen- und/oder Chemotherapie. Die Frage, ob die Chemotherapie bereits vor der Operation durchgeführt werden soll, wird derzeit in klinischen Studien geprüft, wird jedoch von namhaften Ärzten als vorteilhaft angesehen. Für ein solches Vorgehen sprechen verschiedene Argumente:
-	Verminderung des Risikos von Fernabsiedelungen, 
-	Verkleinerung des Tumors und damit erhoffte Erleichterung des operativen Eingriffs,
-	Nachweis der Tumorempfindlichkeit auf die Behandlung mit Chemotherapie.
Im Rahmen dieser neoadjuvanten (vor der Operation) Chemotherapie kommt üblicherweise eine Kombinationsbehandlung zum Einsatz. Standard ist ein Platinpräparat (Cisplatin, Carboplatin) und eines der neuen Zytostatika (Navelbine, Gemcitabine, Docetaxel). Neuentwicklungen haben bei fortgeschrittenen Stadien gezeigt, dass eine verbesserte Wirksamkeit durch die zusätzliche Gabe einer Immuntherapie (Antikörper) erzielt werden kann.   
Wir laden Sie nun ein, an einer wissenschaftlichen Untersuchung teilzunehmen. Dabei ist geplant, vor der Operation eine kombinierte Chemotherapie (Cisplatin und Docetaxel) in Kombination mit einer Immuntherapie (Antikörper: Cetuximab) zu applizieren. Wir hoffen durch diese kombinierte Behandlung das Ansprechen des Tumors zu optimieren und damit die Chance auf Heilung zu verbessern. Die Informationen, die man aus dieser klinischen Studie gewinnt, können zur Verbesserung der Behandlung neuer Patienten beitragen. Gleichzeitig erfolgt mit diesem klinischen Projekt ein umfassendes wissenschaftliches  Programm, in dem die Reaktion des Blutes und des Tumors auf die Behandlung untersucht wird. 
Dieses Informationsblatt soll Ihnen zu einem Überblick über Bedeutung, Ablauf und mögliche Nebenwirkungen der Studie verhelfen. Außerdem werden Sie über Ihre Rechte und unsere Verantwortung Ihnen gegenüber informiert.

HINTERGRUND UND ZIELSETZUNG DER STUDIE
Das Ziel dieser Studie ist es, die Wirksamkeit und Verträglichkeit einer Induktionstherapie mit Docetaxel in Kombination mit Cisplatin und Cetuximab vor Operation zu überprüfen. Die zum Einsatz kommenden Medikamente sind in Österreich zugelassen und wurden bereits bei Tausenden von Patienten angewendet. Neu ist lediglich die Kombination dieser Medikamente vor der Operation bei Lungentumoren. 
 

STUDIENABLAUF
Behandlung
Falls Sie sich zur Teilnahme an dieser Studie entscheiden, erhalten Sie eine sogenannte Induktionstherapie mit Docetaxel als einstündige Infusion in eine Armvene am Tag 1 und am Tag 22, Cisplatin als einstündige Infusion in eine Armvene am Tag 1, am Tag 2, am Tag 22 und am Tag 23 und Cetuximab am Tag 1, am Tag 8, am Tag 15, am Tag 22, am Tag 29 und am Tag 36 verabreicht. Die erste Cetuximab Infusion wird Ihnen über 2 Stunden, die weiteren Cetuximab Infusionen  als einstündige Infusionen in eine Armvene verabreicht.    
Am Beginn und am Ende der Induktionstherapie wird durch bildgebende Verfahren (CT, PET) festgestellt, wie gut Sie auf die Therapie angesprochen haben und ob Ihr Tumor nun operativ entfernt werden kann. Wenn eine Operation möglich ist, wird diese durchgeführt.  
Vor jedem Behandlungszyklus wird eine begleitende Behandlung mit Kortikosteroid-Tabletten und mit einem Antihistaminikum (Dibondrin) durchgeführt, um möglichen allergischen Reaktionen und Flüssigkeitsansammlungen im Körper, vorzubeugen. Die Kortikosteroidbehandlung beginnt am Tag vor der Infusion und endet 2 Tage danach. 
Zur Vorbeugung bzw. Behandlung anderer Nebenwirkungen werden Sie von Ihrem betreuendem Arzt sorgfältig überwacht und entsprechend behandelt.

Untersuchungen
Bevor Sie in die Studie aufgenommen werden, werden folgende Untersuchungen durchgeführt, um festzustellen, ob Sie die Voraussetzung für eine Chemotherapie erfüllen:
Eine klinische Untersuchung, eine Blutuntersuchung (60 ml Blut aus einer Armvene), ein Lungenfunktionstest und verschiedene bildgebende Untersuchungen (Computertomographie und PET (Aktivitätsmessung)), wodurch das Ausmaß Ihrer Krankheit festgestellt werden kann.
Während der Behandlung werden im Rahmen der Routineblutabnahmen sieben Mal zusätzlich 20 ml Blut entnommen (dies gilt nur für Patienten, die in Innsbruck, Natters, Zams oder Kufstein behandelt werden), um den Einfluss der Chemotherapie/Immuntherapie auf das Immunsystem zu kontrollieren. Nach dem zweiten Zyklus der Induktionstherapie werden die bildgebenden Untersuchungen und Lungenfunktionstests wiederholt, um über das Ausmaß des Tumors und das Ansprechen auf die Therapie Auskunft zu geben. Diese Untersuchungen werden routinemäßig bei Ihrer Erkrankung auch dann durchgeführt, wenn Sie an der Studie nicht teilnehmen.
Auch nach Beendigung der Behandlung werden Sie gebeten, nach Anweisung Ihres Arztes zu Kontrollterminen in das Krankenhaus zu kommen. Anlässlich dieser Kontrollen werden die oben genannten Routineuntersuchungen je nach Notwendigkeit wiederholt.
Sollte unter der Antikörpertherapie ein Hautausschlag auftreten, ist eine zusätzliche kleine (wenige Millimeter) Hautbiopsie des betroffenen Areals und eines gesunden Areals vorgesehen. Durch die wissenschaftliche Aufarbeitung ist ein besseres Verständnis der immunologischen Effekte zu erwarten. Sie können jedoch diesen Teil der Untersuchungen unabhängig vom weiteren Ablauf der Studie ablehnen. 
Nebenwirkungen
Während der Studie besteht das Risiko von Nebenwirkungen, die Sie mit Ihrem Arzt besprechen sollten. Die häufigsten Nebenwirkungen sind in der Folge aufgelistet, es können aber auch andere, unvorhergesehene Nebenwirkungen auftreten. Um die bekannten Nebenwirkungen zu reduzieren, werden Begleitmedikamente verabreicht.
Unter Docetaxel (Taxotere) kann es zu Übelkeit und/oder Erbrechen, Irritationen der Mundschleimhaut, Durchfall, Haarausfall, Müdigkeit, Veränderungen des Blutbilds (wodurch die Anfälligkeit für Infektionen und lokale Blutungen steigen kann) kommen. Darüber hinaus kann Docetaxel zu vorübergehenden Empfindungsstörungen an Händen und Füßen, Nagelveränderungen (Verfärbung und/oder Ablösung), Hautreaktionen, Gewichtszunahme, Schwellungen der Extremitäten und/oder im Gesicht (mit oder ohne Flüssigkeitsansammlungen um die Lunge oder im Bauchraum) und Überempfindlichkeitsreaktionen führen.
Bei der Behandlung mit Cisplatin kann es zu Übelkeit, Erbrechen, vorübergehenden Empfindungsstörungen an Händen und Füßen, Hörstörungen mit Ohrensausen und Hörverlust (insbesondere hoher Frequenzen), Elektrolytstörungen (zu niedriger Kalium-, Natrium- oder Magnesiumspiegel) sowie zu Nierenfunktionsstörungen kommen.
Die häufigsten Nebenwirkungen von Cetuximab sind Hautausschlag und Müdigkeit, die in der Regel leichter Ausprägung sind. Leichte bis mittelschwere allergische Reaktionen sind sehr selten.   
Alle genannten Nebenwirkungen klingen in der Regel von selbst oder nach entsprechender Behandlung nach einiger Zeit wieder ab. Zur Vorbeugung bzw. Behandlung von Nebenwirkungen werden Sie von Ihrem betreuenden Arzt während der gesamten Studiendauer sorgfältig überwacht und entsprechend behandelt.
Bitte kontaktieren Sie Ihren behandelnden Arzt sofort im Falle von auftretendem Fieber oder Blutungen nach Erhalt einer Chemotherapie.
Wenn Sie Fieber haben, wird Ihr behandelnder Arzt eine Blutuntersuchung durchführen und Ihnen gegebenenfalls Antibiotika verschreiben. Ist die Anzahl Ihrer weißen Blutkörperchen (für die Bekämpfung von Infektionen) gering, kann Ihr Arzt Ihnen auch ein Medikament zur Anregung der Produktion von weißen Blutkörperchen verschreiben.

Informationen für gebärfähige Frauen und zeugungsfähige Männer - Schwangerschaftstest
Während einer Chemotherapie und bis zu einem Jahr danach ist es möglich, dass der Körper keine oder abnormale Spermien produziert, weshalb Männer während dieser Zeit empfängnisverhütende Maßnahmen (z.B. Gebrauch von Kondomen) setzen müssen. Als Frau im gebärfähigen Alter dürfen Sie an der klinischen Prüfung nur teilnehmen, wenn Sie sich verpflichten, während der Dauer dieser klinischen Prüfung eine zuverlässige Art der Empfängnisverhütung (Pille, Spirale) zu praktizieren und ein Arzt vor und einmal monatlich während der Studiendauer das Nichtvorliegen einer Schwangerschaft (Schwangerschaftstest) feststellt. Es wird Ihnen weiters die Durchführung eines Schwangerschaftstests nach Abschluss der Studie empfohlen.
Schwangere und stillende Frauen sind von der Teilnahme an der Studie ausgeschlossen.
Für weitere Informationen bezüglich Risiken und Nebenwirkungen fragen Sie bitte Ihren behandelnden Arzt.


INFORMATION ÜBER FREIWILLIGKEIT UND VORZEITIGE BEENDIGUNGSMÖGLICHKEITEN
Ihre Teilnahme an dieser Studie erfolgt freiwillig. Sie können jederzeit ohne Angabe von Gründen aus der Studie ausscheiden. Die Ablehnung der Teilnahme oder ein vorzeitiges Ausscheiden aus dieser Studie hat keine nachteiligen Folgen für Ihre weitere medizinische Betreuung. Ebenso kann von Seiten des Prüfarztes Ihre Teilnahme an der Studie vorzeitig beendet werden. Im Falle Ihres vorzeitigen Ausscheidens aus der Studie ist es erforderlich, dass Sie sich einer Schlussuntersuchung  unterziehen.  

INFORMATIONEN ÜBER PERSONENSCHADENVERSICHERUNG
Als Teilnehmer an dieser Studie besteht für Sie der gesetzlich vorgeschriebene Versicherungsschutz (Personenschadenversicherung nach § 32 des Arzneimittelgesetztes oder §47 des Medizinproduktegesetzes), der bei der HDI Hannover Versicherung (Adresse: Edelsinnstraße 7-11, 1120 Wien, Tel.: 0050905501-420) unter der Versicherungspolizze-Nr.: 23764 (vormals 64/16921/16) für diese Studie abgeschlossen wurde.  Im Schadensfall können Sie sich auch direkt an den Versicherer wenden und Ihre Ansprüche geltend machen. Für den Versicherungsvertrag ist österreichisches Recht anwendbar, die Versicherungsansprüche sind in Österreich einklagbar. 
Um den Versicherungsschutz nicht zu gefährden, dürfen Sie sich während der Dauer der klinischen Prüfung einer anderen medizinischen Behandlung nur im Einvernehmen mit dem Prüfarzt unterziehen (Notfälle ausgenommen); dies gilt auch für die Einnahme von Medikamenten.
Sie können aus der Studie herausgenommen werden, falls erkannt wird, dass Sie die Studienbedingungen nicht erfüllen, eine weitere Behandlung ein Gesundheitsrisiko für Sie darstellen würde, Sie die Behandlungsanweisungen nicht beachten oder die Studie abgebrochen wird.

HINWEIS AUF DATENSCHUTZ
Die im Rahmen der Studie erhobenen Daten werden in anonymisierter Form (d.h. ohne Angabe Ihres Namens, lediglich mit Ihren Initialen versehen) gesammelt und ausgewertet. Über Aufforderung werden die Daten den Gesundheits- bzw. Registrierungsbehörden zugänglich gemacht. Mit der Teilnahme an dieser Studie geben Sie Ihre Zustimmung zur Einsichtnahme von autorisierten Personen in Ihre Krankengeschichte, zur Weitergabe der anonymisierten Daten an Dritte, z.B. an das Statistikinstitut, sowie zur Speicherung und elektronischen Verarbeitung im Rahmen des Datenschutzes. In etwaigen Berichten oder Publikationen über die Ergebnisse dieser Studie werden Sie nicht namentlich genannt.
Sie werden über alle neuen wissenschaftlichen Erkenntnisse informiert, die im Verlaufe der Studie bekannt werden und Ihre Bereitschaft zur weiteren Teilnahme beeinflussen könnten. Diese Studie wurde dem Bundesministerium für soziale Sicherheit und Generationen gemeldet und von der zuständigen Ethikkommission genehmigt.  Für weitere Informationen steht Ihnen Ihr Arzt gerne zur Verfügung.
EINVERSTÄNDNISERKLÄRUNG

Meine Teilnahme an der klinischen Prüfung ist freiwillig. Es steht mir frei, meine Einwilligung zur Teilnahme jederzeit und ohne Angabe von Gründen zu widerrufen, ohne dass mir dadurch Nachteile für die weitere Behandlung meiner Erkrankung entstehen.
Ich bin einverstanden, dass die in der Studie erhobenen Daten in anonymisierter Form erfasst werden und gebe auch die Zustimmung, dass autorisierte Personen Einblick in meine Krankenakte nehmen dürfen.
Ich habe die Patienteninformation und Einverständniserklärung gelesen und verstanden. Alle meine Fragen wurden beantwortet, und ich habe zur Zeit keine weiteren Fragen mehr. Sollten sich während der Studie Fragen ergeben, kann ich mich jederzeit wenden an:

Frau/Herrn Prof./Dr. ........................................................................................................

Abteilung für   ............................................		Telefon   ........................................

Ich gebe hiermit freiwillig meine Zustimmung zur Teilnahme an der klinischen Studie. Eine Kopie dieser Einverständniserklärung wurde mir ausgehändigt. Ich bin/ bin nicht (Nichtzutreffendes streichen) einverstanden, bei Hautnebenwirkungen eine Hautbiopsie durchführen zu lassen.

_____________________________
                 Ort und Datum

_____________________________			_____________________________
Name des Patienten (Blockbuchstaben)	            Unterschrift des Patienten

_____________________________
                 Ort und Datum
_____________________________			_____________________________
  Name des verantwortlichen Arztes	                       Unterschrift des verantwortlichen Arztes
17.8.	
APPENDIX X: ECOG PERFORMANCE STATUS

 

Grade	ECOG	
0	Fully active, able to carry on all pre-disease performance without restriction	
1 	Restricted in physically strenuous activity but ambulatory and able to carry out work of a light or sedentary nature, e.g., light house work, office work	
 2	Ambulatory and capable of all self care but unable to carry out work activities. Up and about more than 50% of waking hours  	
 3	Capable of only limited self- care, confined to bed or chair more than 50% of waking hours	
 4	Completely disabled. Cannot carry on any self-care. Totally confined to bed or chair	
 5	Dead	


17.9.	APPENDIX XI: Scientific Project: Definition of chemoresistance by pharmacogenomic testing (This project only applies to centers in Tyrol: Innsbruck, Natters, Zams, Kufstein)
Laboratory Team:
	Dr. S. Pircher
	Dr. J. Löffler
	Dr. M. Dlaska
	Prof. Dr. W. Hilbe	
	Prof. Dr. H. Zwierzina	

17.9.1.	Proteomics and genetic alterations 
The global analysis of cellular proteins has recently been termed proteomics and is a key area of research that is developing in the post-genome era [46]. It involves the simultaneously separation, identification and characterisation of thousands of proteins present in a biological sample in a single procedure. Currently, fluorescent dyes technique such as two-dimensional differential in gel electrophoresis (2-D DIGE) is the core technology, separating proteins on the basis of charge in a first dimension and molecular mass in the second dimension. Changes in the expression of proteins can be identified by comparing the protein spots present in samples from healthy volunteers and cancer patients or before and after therapy with anti-cancer drugs. 
Resistance of cancer cells to chemotherapy is caused by multiple factors, such as MDR1 gene expression [47] or Bcl-2 and AKT overexpression [48] and precise mechanisms are not understood. Recently, it was demonstrated that cisplatin resistance of non-small-cell lung cancer was associated with increased expression of the levels of ERCC1 mRNA. While patients with lower levels of ERCC1 mRNA expression demonstrated better survival, difference in the response rate has been not significant [49]. Proteins are the main functional output and neither the genomic DNA code of an organism nor the amount of mRNA that is expressed for each gene product yields an accurate picture on the state of a living cell [50], which can be altered by many conditions. Proteome profiling complements the genome initiatives and is increasingly used to address biomedical questions, providing insights into new disease mechanisms, identification of novel diagnostic markers and therapeutic targets.
17.9.2.	Methods 
17.9.2.1.	RNA/Protein fractionation 
For sample preparation, tissue is homogenized in lysis solution and total RNA is purified using NucleSpin®RNA II kit (Macherey-Nagel GmbH, Dueren, Germany). Protein fraction is precipitated using methanol/chloroform according to protocol [51]
17.9.2.2.	Two-dimensional differential in gel electrophoresis (2-D DIGE)
Precipitated proteins are then resolubilized in lysis solution (7 M urea, 2 M thiourea, 40 mM Tris base, 1% C7BZO [Sigma-Aldrich Handels GmbH, Vienna, Austria]). Protein concentrations will be determined with a commercial protein assay (BioRad Laboratories, Hercules, CA, USA); 30 µg of proteins in 20 µL of lysis solution are labelled with 180 pmol of CyDye DIGE Fluor minimal dyes (Amersham Biosciences, Vienna, Austria) [Cy3, Cy5 for sample or Cy2 for internal control] for 30 minutes, resuspended in 280 µL of rehydration buffer (7 M urea, 2 M thiourea, 1% C7BZO, 0.5% IPG buffer, DTT 60 mM) and loaded on immobilized 18-cm pH 3-10 NL gradient strips. For the first-dimension active rehydration (50 V) was carried out at 20°C for 12 hours. Isoelectric focusing is performed at 250 V for 30 minutes, 500 V for one hour, 2000 V for one hour and finally at 8000 V until 35000 V/hour are reached in total. For the second dimension, samples are separated on 12.5% polyacrylamide gels with the Ettan Dalttwelve System following the standard procedure recommended by the manufacturer (Amersham Biosciences, Vienna, Austria). After electrophoresis, gels will be scanned using a TyphoonTM 94100 Imager at 100 dpi resolution (Amersham Biosciences, Vienna, Austria). Statistical analysis of changed protein between samples will be performed using DeCyder DIA (Difference In-gel Analysis) and DeCyder Biological Variation Analysis (BVA) software (Amersham Biosciences, Vienna, Austria), setting Student T Test to < 0.05.
17.9.2.3.	Western blot analysis
Precipitated proteins are dissolved in 1x loading buffer. Proteins concentrations were determined with a commercial protein assay (BioRad Laboratories, Hercules, CA, USA). 40 µg of protein per lane are separated by 12% SDS-polyacrylamide gel electrophoresis and electroblotted onto nitrocellulose membranes (Schleicher & Schuell, Dassel, Germany). Protein loading is controlled by Ponceau red staining of membranes. After blocking for one hour in Tris-buffered saline (TBS) supplemented with 5% nonfat milk and 0.1% Tween 20 (Sigma-Aldrich Handels GmbH, Vienna, Austria), membranes are incubated for one hour at room temperature in blocking buffer containing mAb or anti--tubulin (Oncogene Research, Cambridge, MA, USA) antibody as loading control. Membranes are washed three times in TBS¯Tween and incubated for one hour with horseradish peroxidase-conjugated secondary antibody (Amersham, Les Ulis, France). Immunodetection is performed with a chemoluminescence system (ECL, Amersham Biosciences, Vienna, Austria).
17.9.3.	Sample preparation
17.9.3.1.	Serum samples 0.5 mL per vial (four cryostate vials) will be taken at day 0, at week two and monthly thereafter until the end of study.
·	ELISA
·	Western blot to confirm protein expression after 2-D DIGE
·	Immunoprecipitation followed by Western blot, 2-D DIGE
Storage of vials: the vials of serum must be stored at -80°C
17.9.3.2.	Tissue probes will be taken out of the surgical specimens.
·	RT-PCR
·	2-D DIGE
·	immunohistochemistry
Storage of vials: the vials of biopsies must be stored in liquid nitrogen


Reference List Appendix 16.9.

	1. 	Wilkins MR, Sanchez JC, Gooley AA, Appel RD, Humphery-Smith I, Hochstrasser DF et al. Progress with proteome projects: why all proteins expressed by a genome should be identified and how to do it. Biotechnol Genet Eng Rev. 1996;1319-50.
	2. 	Yeh JJ, Hsu WH, Wang JJ, Ho ST, Kao A. Predicting chemotherapy response to paclitaxel-based therapy in advanced non-small-cell lung cancer with P-glycoprotein expression. Respiration. 2003 Jan;70(1):32-35.
	3. 	Janmaat ML, Kruyt FA, Rodriguez JA, Giaccone G. Response to epidermal growth factor receptor inhibitors in non-small cell lung cancer cells: limited antiproliferative effects and absence of apoptosis associated with persistent activity of extracellular signal-regulated kinase or Akt kinase pathways. Clin Cancer Res. 2003 Jun;9(6):2316-2326.
	4. 	Rosell R, Cuello M, Cecere F, Santarpia M, Reguart N, Felip E et al. Treatment of non-small-cell lung cancer and pharmacogenomics: where we are and where we are going. Curr Opin Oncol. 2006 Mar;18(2):135-143.
	5. 	Anderson L, Seilhamer J. A comparison of selected mRNA and protein abundances in human liver. Electrophoresis. 1997 Mar;18(3-4):533-537.
	6. 	Wessel D, Flugge UI. A method for the quantitative recovery of protein in dilute solution in the presence of detergents and lipids. Anal Biochem. 1984 Apr;138(1):141-143.
17.10.	
APPENDIX XII: Project Proposal: Immune activation after application of Erbitux in combination with chemotherapy in NSCLC patients – correlation with CD4+CD25+ regulatory T-cells number  and functional activity (This project only applies to centers in Tyrol: Innsbruck, Natters, Zams, Kufstein)

Applicants:	Dominik Wolf*
				Anna-Maria Wolf*
				Holger Rumpold*
				Wolfgang Hilbe**

Affiliation:	*   Dpt. Hematology and Oncology
		    	Medical University Innsbruck
	   	    	Anichstr. 35
		    	6020 Innsbruck
		    	Austria
		    	Tel.: ++43-512-504-81385
		    	E-mail: dominik.wolf@i-med.ac.at

			**  Dpt. Hematology and Oncology
		     	Internal Medicine
    	Medical University Innsbruck
	   	   	Anichstr. 35
		   	6020 Innsbruck
		   	Austria
		   	Tel:: ++43-512-504-81151
		   	E-mail: wolfgang.hilbe@i-med.ac.at

17.10.1.	Introduction:
The combination of antibodies directed against tumor antigens, such as Her2/neu, CD20 and EGFR has proven clinical efficacy. The mechanisms involved in the improvement of response rates by antibody therapies remains elusive so far. Direct effects on tumor cells (i.e. modulation cell growth and/or direct induction of apoptosis) as well as immune-modulatory effects (i.e. opsonization of tumor cells with subsequent ADCC by immune cells) might both be involved in the in vivo effects of antibody therapy. ADCC describes the antibody-dependent killing of tumor cells by NK-cells, monocytes and macrophages and might play a central role for the efficacy of antibody-treatment in vivo.
Recently, CD4+CD25+ regulatory T-cells (Treg) have been shown to play a critical role for maintenance of tolerance in rodents and men. Early data already suggested that depletion of Treg enhances anti-tumor immunity. It is noteworthy that Treg actively proliferate in patients suffering from epithelial malignancies, as well as the amount of intratumoral Treg is of prognostic relevance for example in patients suffering from ovarian cancer. The observed increase of regulatory T-cells in cancer patients might impede immune-mediated therapies, such as vaccination approaches or antibody-therapy. Preclinical data already provided evidence that depletion of Treg prior to tumor- or dendritic-cell vaccination profoundly improved the vaccine-induced anti-tumor immune response. This has led to early clinical trials in melanoma patients following the concept of Treg depletion and subsequent application of peptide-based vaccines. The aim of the current project is to determine the intensity of immune activation in lung cancer patients while and after treatment with Erbitux in combination with chemotherapy (taxanes together with cisplatin or carboplatin). Clinical response and the amount of immune activation will be correlated to the content of Treg in peripheral blood prior to therapy.

17.10.2.	Aim of the project:
17.10.2.1.	To determine absolute numbers of Treg in peripheral blood of lung cancer patients before and under therapy with Erbitx and chemotherapy. The amount of Treg will be correlated to clinical response. In addition, the immunsuppressive capacity of Treg will be determined prior and under therapy with Erbitux/chemotherapy.
17.10.2.2.	To isolate PBMC from cancer patients prior to therapy and determine their in vitro killing capacity against EGFR positive and negative lung cancer cell lines prior and after application of Erbitux and prior and after depletion of Treg.
17.10.2.3.	To determine immune activation markers (release of IL-2, soluble TRAIL, TNF, soluble TNF-R1 and TNF-R2, soluble IL-2 receptor, granzymeA and B, complement factors) before, under and after application of Erbitux together with chemotherapy and correlate these markers with clinical response parameters.
17.10.3.	Detailed Description of Methods:
Ad 17.10.2.1: Treg will be determined before therapy and weekly using a 3-colour FACS analysis (FACSCalibur, BD) with the following 3 antibodies:

1.)	CD4 APC
2.)	CD25 PE
3.)	FoxP3

Treg are determined by their expression of : CD4int, CD25high, FoxP3+

The total number of Treg/µl blood will determined by quantification of CD4intCD25high cells/µl whole blood using True-count beads and by the following equitation:

Total number of Treg/µl blood  = CD4intCD25highFoxP3+ T-cells x  CD4intCD25high/µl.

In parallel, mRNA will be isolated from whole blood of each individual patient for subsequent determination of FoxP3 transcripts by real-time PCR. We have set up a method for the quantification of total FoxP3 mRNA transcripts per cell number or per µg of transcribed RNA. This will be done by dilution series of a plasmid standard (each dilution containing known copy numbers of the plasmid), which enables the absolute quantification of FoxP3 transcripts.

Quantification of Treg will be done in a fixed schedule, i.e. prior to therapy, and every cycle thereafter (until therapy with Erbitux, Docetaxel and CDDP is finished). After neo-adjuvant therapy Treg will be monitored prior to surgery and at the end of treatment (30-45 days after surgery).

Treg are known to have characteristic properties, such as anergy to TCR-stimulation and immunosuppressive capacities when co-cultured with CD4+CD25- T-cells. Therefore, the functional properties of Treg will be determined before and under therapy with Erbitux/chemotherapy. Functional analyses will be performed every 4 weeks. Therefore, Treg will be iosolated using magnetic beads for selection (Milteny Biotech, Bergisch Gladbach, Germany). Purity will be controlled by FACS. The assays will be done as follows:

Proliferation-Assay:
·	1 x 10^6^ Treg or control cells (CD4+CD25-)/ml RPMI 10% FCS+1% GPS 
·	96-well plates (round-bottom) will be coated with anti-CD3 (5 µg OKT3 /ml in PBS): 1h, 37°C, 50 µl/well
·	at least 3 wells will be filled with either CD4+CD25+ or CD4+CD25- cells alone:
- CD4+CD25- (1,5x10^5^/well)
	- CD4+CD25+ (1,5x10^5^/well)
[= 1,5x10^5^/well final cell conc., final volume 150 µl/well]
·	[3 H]thymidine will be added after 5 days for further 16 hours


Suppressions-Assay:
·	at least 3 wells will be filled with irradiated CD4+CD25+ (1,5x10^5^) together with CD4+CD25- (1,5x 10^5^) cell (=ratio 1:1) and
at least 3 wells of irradiated CD4+CD25- (1,5x10^5^)  together with CD4+CD25- (1,5x   105) cells (=ratio 1:1)   
 	[= 3x10^5^/well final concentration, final volume 150 µl/well]
·	[3 H]thymidine will be added after 5 days for further 16 hours

Ad 17.10.2.2:To determine the functional impact of Treg expansion for antibody-mediated cellular cytotoxicity (ADCC), 20 ml whole blood will be drawn from each patient before start of therapy. To test the functional impact, the following in vitro model will be used.
Target cells for in vitro cytotoxicity testing:

1.	EGFR- cell line: control cell line, negative for EGFR proven by RT-PCR, immunohistochemistry and FACS.
2.	EGFR+ cell line: EGFR will be overexpressed in the EGFR-control cell line with the help of the sleeping beauty system, allowing the stable overexpression via transposase-dependent integration of the transposon into genomic DNA. We have profound experience with this vector system. The cDNA will be cloned from the EGFR positive SCLC cell line and subsequently sequenced before subcloning into the sleeping beauty expression vector (pT2-neoCIMS). Monoclonal cell lines overexpressing EGFR will be selected in semisolid medium (Clonacell). 

Target cells will be labelled with 51Cr to determined the cell-mediated lysis after co-culture with the immune cells.  Each cell line will be incubated with either whole PBMC and PBMC depleted of CD25+ cells (which are almost exclusively Treg, thereby giving the Treg-depleted PBMC fraction). Target cell lines will be either preincubated with Erbitux or an irrelevant antibody, to determine the effect of antibody-mediated cytotoxicity. The increase of cytotoxicity induced by prior CD25+ T-cell depletion in vitro will be determined and correlated to clinical response parameters. This will help to determine, whether prior depletion of Treg to antibody-therapies might be a suitable way to increase the efficacy of antibody-dependent cytotoxicity.
Ad 17.10.2.3: To study the activation of the immune system after application of Erbitux/together with chemotherapy, a variety of surrogate markers for T-cell mediated immune responses (release of IL-2, soluble TRAIL, TNF, soluble TNF-R1 and TNF-R2, soluble IL-2 receptor, granzymeA and B, complement factors) will be measured. Therefore, serum of each patient will be harvested prior and every 7 days thereafter throughout therapy, and after the end of therapy. The respective surrogate markers will be determined using commercially available ELISA kits. 

Literature Appendix 16.10.:


1.	Sakaguchi, S. Annu.Rev.Immunol. 2004, 22, 531.
2.	Onizuka, S.; Tawara, I.; Shimizu, J.; Sakaguchi, S.; Fujita, T.; Nakayama, E. Cancer Res. 1999, 59, 3128.
3.	Shimizu, J.; Yamazaki, S.; Sakaguchi, S. J.Immunol. 1999, 163, 5211.
4.	Wang, H. Y.; Lee, D. A.; Peng, G.; Guo, Z.; Li, Y.; Kiniwa, Y.; Shevach, E. M.; Wang, R. F. Immunity. 2004, 20, 107.
5.	Camara, N. O.; Sebille, F.; Lechler, R. I. Eur.J.Immunol. 2003, 33, 3473.
6.	Janssens, W.; Carlier, V.; Wu, B.; VanderElst, L.; Jacquemin, M. G.; Saint-Remy, J. M. J.Immunol. 2003, 171, 4604.
7.	Seo, S. J.; Fields, M. L.; Buckler, J. L.; Reed, A. J.; Mandik-Nayak, L.; Nish, S. A.; Noelle, R. J.; Turka, L. A.; Finkelman, F. D.; Caton, A. J.; Erikson, J. Immunity. 2002, 16, 535.
8.	Nakamura, K.; Kitani, A.; Strober, W. J.Exp.Med. 2001, 194, 629.
9.	Chen, M. L.; Pittet, M. J.; Gorelik, L.; Flavell, R. A.; Weissleder, R.; von Boehmer, H.; Khazaie, K. Proc.Natl.Acad.Sci.U.S.A 2005, 102, 419.
10.	Fontenot, J. D.; Gavin, M. A.; Rudensky, A. Y. Nat.Immunol. 2003, 4, 330.
11.	Hori, S.; Nomura, T.; Sakaguchi, S. Science 2003, 299, 1057.
12.	Woo, E. Y.; Chu, C. S.; Goletz, T. J.; Schlienger, K.; Yeh, H.; Coukos, G.; Rubin, S. C.; Kaiser, L. R.; June, C. H. Cancer Res. 2001, 61, 4766.
13.	Liyanage, U. K.; Moore, T. T.; Joo, H. G.; Tanaka, Y.; Herrmann, V.; Doherty, G.; Drebin, J. A.; Strasberg, S. M.; Eberlein, T. J.; Goedegebuure, P. S.; Linehan, D. C. J.Immunol. 2002, 169, 2756.
14.	Schaefer, C.; Kim, G. G.; Albers, A.; Hoermann, K.; Myers, E. N.; Whiteside, T. L. Br.J.Cancer 2005, 92, 913.
15.	Ormandy, L. A.; Hillemann, T.; Wedemeyer, H.; Manns, M. P.; Greten, T. F.; Korangy, F. Cancer Res. 2005, 65, 2457.
16.	Ichihara, F.; Kono, K.; Takahashi, A.; Kawaida, H.; Sugai, H.; Fujii, H. Clin.Cancer Res. 2003, 9, 4404.
17.	Sasada, T.; Kimura, M.; Yoshida, Y.; Kanai, M.; Takabayashi, A. Cancer 2003, 98, 1089.
18.	Viguier, M.; Lemaitre, F.; Verola, O.; Cho, M. S.; Gorochov, G.; Dubertret, L.; Bachelez, H.; Kourilsky, P.; Ferradini, L. J.Immunol. 2004, 173, 1444.
19.	Marshall, N. A.; Christie, L. E.; Munro, L. R.; Culligan, D. J.; Johnston, P. W.; Barker, R. N.; Vickers, M. A. Blood 2004, 103, 1755.
20.	Curiel, T. J.; Coukos, G.; Zou, L.; Alvarez, X.; Cheng, P.; Mottram, P.; Evdemon-Hogan, M.; Conejo-Garcia, J. R.; Zhang, L.; Burow, M.; Zhu, Y.; Wei, S.; Kryczek, I.; Daniel, B.; Gordon, A.; Myers, L.; Lackner, A.; Disis, M. L.; Knutson, K. L.; Chen, L.; Zou, W. Nat.Med. 2004, 10, 942.
21.	Wang, H. Y.; Peng, G.; Guo, Z.; Shevach, E. M.; Wang, R. F. J.Immunol. 2005, 174, 2661.
22.	Wolf D, Wolf AM, Rumpold H, Fiegl H, Zeimet AG, Muller-Holzner E, Deibl M, Gastl G, Gunsilius E, Marth C. The Expression of the Regulatory T-cell specific Forkhead Box Transcription Factor FoxP3 is Associated with Poor Prognosis in Ovarian Cancer. Clin. Cancer Res. In press.
23.	Wolf AM, Wolf D, Steurer M, Gastl G, Gunsilius E, Grubeck-Loebenstein B. Increase of regulatory T-cells in the peripheral blood of cancer patients. Clin Cancer Res. 2003 Feb;9(2):606-12.
24.	Wolf D, Wolf AM, Rumpold H, Koppelstätter C, Gastl G, Mayer G, Gunsilius E, and Tilg H. Telomerase activity and telomere length of CD4+CD25+ regulatory T-cells under conditions of in vitro and in vivo expansion. Canc Immunol Immunother. In press.


Reference List

[1] Boring, C. C.; Squires, T. S.; Tong, T. Cancer statistics, 1992. CA Cancer J. Clin 1992, 42, 19-38.
[2] Mattson, K. Docetaxel (Taxotere) and neoadjuvant chemotherapy for non-small cell lung cancer. Semin. Oncol 1999, 26, 25-28.
[3] Strauss, G. M.; Langer, M. P.; Elias, A. D.; Skarin, A. T.; Sugarbaker, D. J. Multimodality treatment of stage IIIA non-small-cell lung carcinoma: a critical review of the literature and strategies for future research. J. Clin Oncol 1992, 10, 829-838.
[4] Ihde, D. C. Neoadjuvant chemotherapy for non-small cell lung cancer: current North American experience. Semin. Oncol 1988, 15, 3-7.
[5] Einhorn, L. H. Neoadjuvant therapy of stage III non-small cell lung cancer. Ann. Thorac. Surg. 1988, 46, 362-365.
[6] Shepherd, F. A. Induction chemotherapy for locally advanced non-small cell lung cancer. Ann. Thorac. Surg. 1993, 55, 1585-1592.
[7] Rosell, R.; Gomez-Codina, J.; Camps, C.; Javier, S. J.; Maestre, J.; Padilla, J.; Canto, A.; Abad, A.; Roig, J. Preresectional chemotherapy in stage IIIA non-small-cell lung cancer: a 7-year assessment of a randomized controlled trial. Lung Cancer 1999, 26, 7-14.
[8] Roth, J. A.; Fossella, F.; Komaki, R.; Ryan, M. B.; Putnam, J. B., Jr.; Lee, J. S.; Dhingra, H.; De Caro, L.; Chasen, M.; McGavran, M.; . A randomized trial comparing perioperative chemotherapy and surgery with surgery alone in resectable stage IIIA non-small-cell lung cancer. J. Natl. Cancer Inst. 1994, 86, 673-680.
[9] Depierre, A.; Milleron, B.; Moro-Sibilot, D.; Chevret, S.; Quoix, E.; Lebeau, B.; Braun, D.; Breton, J. L.; Lemarie, E.; Gouva, S.; Paillot, N.; Brechot, J. M.; Janicot, H.; Lebas, F. X.; Terrioux, P.; Clavier, J.; Foucher, P.; Monchatre, M.; Coetmeur, D.; Level, M. C.; Leclerc, P.; Blanchon, F.; Rodier, J. M.; Thiberville, L.; Villeneuve, A.; Westeel, V.; Chastang, C. Preoperative chemotherapy followed by surgery compared with primary surgery in resectable stage I (except T1N0), II, and IIIa non-small-cell lung cancer. J. Clin. Oncol. 2002, 20, 247-253.
[10] Winton, T.; Livingston, R.; Johnson, D.; Rigas, J.; Johnston, M.; Butts, C.; Cormier, Y.; Goss, G.; Inculet, R.; Vallieres, E.; Fry, W.; Bethune, D.; Ayoub, J.; Ding, K.; Seymour, L.; Graham, B.; Tsao, M. S.; Gandara, D.; Kesler, K.; Demmy, T.; Shepherd, F. Vinorelbine plus cisplatin vs. observation in resected non-small-cell lung cancer. N. Engl. J. Med. 2005, 352, 2589-2597.
[11] Arriagada, R.; Bergman, B.; Dunant, A.; Le Chevalier, T.; Pignon, J. P.; Vansteenkiste, J. Cisplatin-based adjuvant chemotherapy in patients with completely resected non-small-cell lung cancer. N. Engl. J. Med. 2004, 350, 351-360.
[12] Dillman, R. O.; Herndon, J.; Seagren, S. L.; Eaton, W. L., Jr.; Green, M. R. Improved survival in stage III non-small-cell lung cancer: seven-year follow-up of cancer and leukemia group B (CALGB) 8433 trial. J. Natl. Cancer Inst. 1996, 88, 1210-1215.
[13] Sause, W. T.; Scott, C.; Taylor, S.; Johnson, D.; Livingston, R.; Komaki, R.; Emami, B.; Curran, W. J.; Byhardt, R. W.; Turrisi, A. T.; . Radiation Therapy Oncology Group (RTOG) 88-08 and Eastern Cooperative Oncology Group (ECOG) 4588: preliminary results of a phase III trial in regionally advanced, unresectable non-small-cell lung cancer. J. Natl. Cancer Inst. 1995, 87, 198-205.
[14] Sause, W.; Kolesar, P.; Taylor S IV; Johnson, D.; Livingston, R.; Komaki, R.; Emami, B.; Curran, W., Jr.; Byhardt, R.; Dar, A. R.; Turrisi, A., III Final results of phase III trial in regionally advanced unresectable non-small cell lung cancer: Radiation Therapy Oncology Group, Eastern Cooperative Oncology Group, and Southwest Oncology Group. Chest 2000, 117, 358-364.
[15] Marino, P.; Preatoni, A.; Cantoni, A. Randomized trials of radiotherapy alone versus combined chemotherapy and radiotherapy in stages IIIa and IIIb nonsmall cell lung cancer. A meta-analysis. Cancer 1995, 76, 593-601.
[16]  Chemotherapy in non-small cell lung cancer: a meta-analysis using updated data on individual patients from 52 randomised clinical trials. Non-small Cell Lung Cancer Collaborative Group. BMJ 1995, 311, 899-909.
[17] Furuse, K.; Fukuoka, M.; Kawahara, M.; Nishikawa, H.; Takada, Y.; Kudoh, S.; Katagami, N.; Ariyoshi, Y. Phase III study of concurrent versus sequential thoracic radiotherapy in combination with mitomycin, vindesine, and cisplatin in unresectable stage III non-small-cell lung cancer. J. Clin. Oncol. 1999, 17, 2692-2699.
[18] Furuse, K.;.Hosoe, S.;.Masuda, N.; et al. Impact of tumor control on survival in unresectable stage III non-small cell lung cancer (NSCLC) treated with concurrent thoracic radiotherapy and chemotherapy. Proc Am Soc Clin Oncol 2000, 484a.
[19] Curran, W.;.Scott, C.;.Langer, C.; et al. Long-term benefit is observed in a phase III comparison of sequential vs concurrent chemo-radiation for patients with unresected stage III non small cell lung cnacer: RTOG 9410. Proc Am Soc Clin Oncol 2003, 621a.
[20] Albain, K. S.; Crowley, J. J.; Turrisi, A. T., III; Gandara, D. R.; Farrar, W. B.; Clark, J. I.; Beasley, K. R.; Livingston, R. B. Concurrent cisplatin, etoposide, and chest radiotherapy in pathologic stage IIIB non-small-cell lung cancer: a Southwest Oncology Group phase II study, SWOG 9019. J. Clin Oncol 2002, 20, 3454-3460.
[21] Gandara, D. R.; Chansky, K.; Albain, K. S.; Leigh, B. R.; Gaspar, L. E.; Lara, P. N., Jr.; Burris, H.; Gumerlock, P.; Kuebler, J. P.; Bearden, J. D., III; Crowley, J.; Livingston, R. Consolidation docetaxel after concurrent chemoradiotherapy in stage IIIB non-small-cell lung cancer: phase II Southwest Oncology Group Study S9504. J. Clin Oncol 2003, 21, 2004-2010.
[22] de Perrot, M.; Fadel, E.; Mussot, S.; de Palma, A.; Chapelier, A.; Dartevelle, P. Resection of locally advanced (T4) non-small cell lung cancer with cardiopulmonary bypass. Ann. Thorac. Surg. 2005, 79, 1691-1696.
[23]  Taxotere Investigator's Brochure.1-9-1999,
[24] Fossela, F. V.; et al. Taxotere (docetaxel), an active agent for platinum-refractory non-small-cell lung cancer: preliminary report of a phase II study. Proc Am Soc Clin Oncol 1994, 336.
[25] Bizzari, J. P. Docetaxel RP56976, integrated summary, advanced or metastatic non-small-cell lung cancer.16-7-1994, Aventis.
[26] Fossela, F. V.; et al. Phase III trial of docetaxel 100mg/m2 or 75mg/m2 vs. Vinorelbine/Isfosfamide for non-small-cell lung cancer previously treated with platinum-based chemotherapy. Proc Am Soc Clin Oncol 1999, 460a.
[27] Fossella, F. V.; Lee, J. S.; Berille, J.; Hong, W. K. Summary of phase II data of docetaxel (Taxotere), an active agent in the first- and second-line treatment of advanced non-small cell lung cancer. Semin. Oncol 1995, 22, 22-29.
[28] Miller, V. A.; Rigas, J. R.; Francis, P. A.; Grant, S. C.; Pisters, K. M.; Venkatraman, E. S.; Woolley, K.; Heelan, R. T.; Kris, M. G. Phase II trial of a 75-mg/m2 dose of docetaxel with prednisone premedication for patients with advanced non-small cell lung cancer. Cancer 1995, 75, 968-972.
[29] Pronk, L. C.; Schellens, J. H.; Planting, A. S.; van den Bent, M. J.; Hilkens, P. H.; van der Burg, M. E.; Boer-Dennert, M.; Ma, J.; Blanc, C.; Harteveld, M.; Bruno, R.; Stoter, G.; Verweij, J. Phase I and pharmacologic study of docetaxel and cisplatin in patients with advanced solid tumors. J. Clin Oncol 1997, 15, 1071-1079.
[30] Berille, J.; et al. Phase I/II study of RP56976 and cisplatin combination chemotherapy in patients with unresectable metastatic and/or locally advanced non-small-cell lung carcinoma, Taxx018 study, final study report.1997,
[31] Millward, M. J.; Zalcberg, J.; Bishop, J. F.; Webster, L. K.; Zimet, A.; Rischin, D.; Toner, G. C.; Laird, J.; Cosolo, W.; Urch, M.; Bruno, R.; Loret, C.; James, R.; Blanc, C. Phase I trial of docetaxel and cisplatin in previously untreated patients with advanced non-small-cell lung cancer. J. Clin Oncol 1997, 15, 750-758.
[32] Belani, C. P.; et al. Docetaxel and cisplatin combination in patients with non-small-cell lung cancer(NSCLC): a multicenter phase II trial. Lung Cancer 1997, 37.
[33] Zalcberg, J.; Millward, M.; Bishop, J.; McKeage, M.; Zimet, A.; Toner, G.; Friedlander, M.; Barter, C.; Rischin, D.; Loret, C.; James, R.; Bougan, N.; Berille, J. Phase II study of docetaxel and cisplatin in advanced non-small-cell lung cancer. J. Clin Oncol 1998, 16, 1948-1953.
[34] Fossella, F.; Pereira, J. R.; von Pawel, J.; Pluzanska, A.; Gorbounova, V.; Kaukel, E.; Mattson, K. V.; Ramlau, R.; Szczesna, A.; Fidias, P.; Millward, M.; Belani, C. P. Randomized, multinational, phase III study of docetaxel plus platinum combinations versus vinorelbine plus cisplatin for advanced non-small-cell lung cancer: the TAX 326 study group. J. Clin Oncol 2003, 21, 3016-3024.
[35] Betticher, D. C.; et al. Neoadjuvant chemotherapy with docetaxel and cisplatin in patients with NSCLC, stage IIIA, N2 is highly active with few toxicities. ASCO 1999, 1824.
[36] Rosenberg, B.; VanCamp, L.; Trosko, J. E.; Mansour, V. H. Platinum compounds: a new class of potent antitumour agents. Nature 1969, 222, 385-386.
[37] Gullo, J. J.; Litterst, C. L.; Maguire, P. J.; Sikic, B. I.; Hoth, D. F.; Woolley, P. V. Pharmacokinetics and protein binding of cis-dichlorodiammine platinum (II) administered as a one hour or as a twenty hour infusion. Cancer Chemother. Pharmacol. 1980, 5, 21-26.
[38] Von Hoff, D. D.; Schilsky, R.; Reichert, C. M.; Reddick, R. L.; Rozencweig, M.; Young, R. C.; Muggia, F. M. Toxic effects of cis-dichlorodiammineplatinum(II) in man. Cancer Treat. Rep. 1979, 63, 1527-1531.
[39]  Cetuximab Investigator's Brochure. Version 11 2005,
[40] Kelly, K.; Hanna, N.; Rosenberg, P.; Bunn, P. A.; Needle, M. N. A multi-centered phase I/II study of cetuximab in combination with paclitaxel and carboplatin in untreated patients with stage IV non-small cell lung cancer. Proc Am Soc Clin Oncol 2003, 22, 644.
[41] Robert, F.; Blumenschein, G. R.; Dicke, K.; Tseng, J.; Saleh, M. N.; Needle, M. Phase Ib/IIa study of anti-epidermal growth factor receptor (EGFR) antibody, cetuximab, in combination with gemcitabine/carboplatin in patients with advanced non-small cell lung cancer (NSCLC). Proc Am Soc Clin Oncol 2003, 22, 643.
[42] Rosell, R.; Daniel, C.; Ramlau, R.; Szczesna, A.; Constenla, M.; Mennecier, B.; Pfeifer, W.; Mueser, M.; Montaner, I.; Gatzemeier, U. Randomized phase II study of cetuximab in combination with cisplatin (C) and vinorelbine (V) vs. CV alone in the first-line treatment of patients (pts) with epidermal growth factor receptor (EGFR)-expressing advanced non-small-cell lung cancer (NSCLC). Proc Am Soc Clin Oncol 2004, 23, 618.
[43] Lynch, T. J.; Lilenbaum, R.; Bonomi, P.; Ansari, R.; Govindan, R.; Janne, P. A.; Hanna, N. A phase II trial of cetuximab as therapy for recurrent non-small cell lung cancer (NSCLC). Proc Am Soc Clin Oncol 2004, 23, 634.
[44] Kim, E. S.; Mauer, A. M.; Tran, H. T.; Liu, D.; Gladish, G.; Dicke, K.; Needle, M. N.; Vokes, E. E.; Hong, W. K.; Herbst, R. S. A phase II study of cetuximab, an epidermal growth factor receptor (EGFR) blocking antibody, in combination with docetaxel in chemotherapy refractory/resistant patients with advanced non-small cell lung cancer: Final report. Proc Am Soc Clin Oncol 2003, 22, 642.
[45] Harding, J.; Burtness, B. Cetuximab: an epidermal growth factor receptor chemeric human-murine monoclonal antibody. Drugs Today (Barc. ) 2005, 41, 107-127.
[46] Wilkins, M. R.; Sanchez, J. C.; Gooley, A. A.; Appel, R. D.; Humphery-Smith, I.; Hochstrasser, D. F.; Williams, K. L. Progress with proteome projects: why all proteins expressed by a genome should be identified and how to do it. Biotechnol. Genet. Eng Rev. 1996, 13, 19-50.
[47] Yeh, J. J.; Hsu, W. H.; Wang, J. J.; Ho, S. T.; Kao, A. Predicting chemotherapy response to paclitaxel-based therapy in advanced non-small-cell lung cancer with P-glycoprotein expression. Respiration 2003, 70, 32-35.
[48] Janmaat, M. L.; Kruyt, F. A.; Rodriguez, J. A.; Giaccone, G. Response to epidermal growth factor receptor inhibitors in non-small cell lung cancer cells: limited antiproliferative effects and absence of apoptosis associated with persistent activity of extracellular signal-regulated kinase or Akt kinase pathways. Clin. Cancer Res. 2003, 9, 2316-2326.
[49] Rosell, R.; Cuello, M.; Cecere, F.; Santarpia, M.; Reguart, N.; Felip, E.; Taron, M. Treatment of non-small-cell lung cancer and pharmacogenomics: where we are and where we are going. Curr. Opin. Oncol 2006, 18, 135-143.
[50] Anderson, L.; Seilhamer, J. A comparison of selected mRNA and protein abundances in human liver. Electrophoresis 1997, 18, 533-537.
[51] Wessel, D.; Flugge, U. I. A method for the quantitative recovery of protein in dilute solution in the presence of detergents and lipids. Anal. Biochem. 1984, 138, 141-143.
